# Supplementary material for: Anaesthesia care providers employed in humanitarian settings by Médecins Sans Frontières: a retrospective observational study of 173 084 surgical cases over 10 years
Source: BMJ Open. 2020 Mar 4;10(3):e034891. doi: 10.1136/bmjopen-2019-034891 (PMC7059447; doi:10.1136/bmjopen-2019-034891)
Supplement: Supplementary data [file bmjopen-2019-034891supp001.pdf]

Anaesthesia care providers employed in humanitarian settings by Médecins Sans Frontières: A retrospective observational study of 173,084 surgical cases over 10 years

APPENDIX

Table of Contents

Appendix Table 1: Variables used in the study

Appendix Table 2: surgical projects in health care gap settings in the WHO SEA region (2 in total)

Appendix Table 3: Surgical projects in natural disaster settings (5 in total)

Appendix Table 4: Surgical groupings as used in main table 2 (case-level data)

Missing data Table 1: Number of missing values within each variable

Missing data Figure 2: Proportion of missing data in each surgical project

Missing data Table 2: overview of available data

Appendix Table 1: Variables used in the study

<table>
<tr><th>Key characteristic group</th><th>Name of variable</th><th>Type of data</th><th>Description of data</th><th>Source</th></tr>
<tr><td rowspan="5">Patient</td><td>Age</td><td>Continuous</td><td>For patients below the age of 2 (typed in original data collection sheet as days and months), the age has been converted for analysis to a fraction of a year.</td><td>Case-level routine data</td></tr>
<tr><td>Gender</td><td>Categorical (binary)</td><td>Either male or female</td><td>Case-level routine data</td></tr>
<tr><td>Date of procedure</td><td>Continuous</td><td>Date operation took place</td><td>Case-level routine data</td></tr>
<tr><td>ASA grade</td><td>Ordinal</td><td>American Society of Anaesthesiologists physical status classification system (ASA). Discrete numeric scale between 1 and 5 (1 = normal healthy patient, 5 = moribund patient expected not to survive without surgery) of the patient’s physical health prior to surgery.</td><td>Case-level routine data</td></tr>
<tr><td>Cause of hospitalisation</td><td>Categorical (nominal)</td><td>3 letter code used as defined by MSF-OCB operational departmental guideline. A total of 24 codes available, and grouped into 4 distinct categories:
- Accidental trauma
- Violent trauma
- Obstetric
- Other (including but not limited to tropical disease, tumours, obstruction)</td><td>Case-level routine data</td></tr>
<tr><td rowspan="4">Surgery</td><td>Urgency</td><td>Categorical (ordinal)</td><td>3 values available, relating to how soon the surgical procedure has to occur:
- “Urgent” (labelled “emergent” in manuscript) = requiring immediate surgery
- “Delayed” (labelled “urgent” in manuscript) = requiring surgery during current hospital admission
- “Planned” (labelled “elective” in manuscript) = elective surgery</td><td>Case-level routine data</td></tr>
<tr><td>Order</td><td>Categorical (ordinal)</td><td>3 codes available, relating to whether patient has had surgery before during admission:
- “First” = first time entering theatre
- “Unplanned” = unplanned return to theatre
- “Re-intervention” = planned return to theatre</td><td>Case-level routine data</td></tr>
<tr><td>Procedure – Main group</td><td>Categorical (nominal)</td><td>2 letter code used as defined by MSF-OCB operational department guidelines. A total of 36 procedures codes available, and grouped into the main categories of surgery. See appendix table 4 for full breakdown.</td><td>Case-level routine data</td></tr>
<tr><td>Lead surgical provider</td><td>Categorical (nominal)</td><td>A merged variable, based on the presence of local and expatriate providers as outlined below. MD signifies a physician without surgical qualifications but with surgical skills.
<table>
<tr><td></td><td>Local provider</td><td>Expatriate provider</td></tr>
<tr><td rowspan="4">General & specialist surgeon</td><td>None</td><td>General & Specialist</td></tr>
<tr><td>MD</td><td>General & Specialist</td></tr>
<tr><td>General</td><td>Specialist</td></tr>
<tr><td>General & Specialist</td><td>Any</td></tr>
<tr><td rowspan="2">Specialist only</td><td>None</td><td>Specialist</td></tr>
<tr><td>MD</td><td>Specialist</td></tr>
</table></td><td>Programme-level routine data (MT)</td></tr>
</table>

<table>
<tr><td rowspan="3"></td><td></td><td></td><td><table><tr><td></td><td>Specialist</td><td>Specialist or none</td></tr><tr><td rowspan="3">General only</td><td>None</td><td>General</td></tr><tr><td>MD</td><td>General</td></tr><tr><td>General</td><td>General or none</td></tr><tr><td>MD</td><td>MD</td><td>None</td></tr></table></td><td></td></tr>
<tr><td>Theatre time (min)</td><td>Continuous</td><td>Time in minutes the patient was occupying theatre. This included anaesthetic and surgical time, as well as any recovery of patient, which occurred in theatre.</td><td>Case-level routine data</td></tr>
<tr><td>Intra-operative mortality</td><td>Categorical (binary)</td><td>Whether the patient was dead or alive by the time they left recovery. For the purpose of the study, this is considered as intraoperative mortality.</td><td>Case-level routine data</td></tr>
<tr><td rowspan="2">Anaesthesia</td><td>Choice of anaesthesia</td><td>Categorical (nominal)</td><td>List of 7 codes of anaesthesia that can be provided. Only a single code can be used for a surgical procedure:
- Local anaesthesia
- Regional anaesthesia
- Spinal anaesthesia
- General anaesthesia without intubation or muscle relaxant
- General anaesthesia with intubation and/or muscle relaxant
- Combined anaesthesia (if more than one code need to be used, e.g. spinal anaesthesia + general anaesthesia)
- Other anaesthesia, e.g. sedation</td><td>Case-level routine data</td></tr>
<tr><td>Lead anaesthesia provider</td><td>Categorical (nominal)</td><td>A merged variable, based on the presence of local and expatriate providers as outlined below.<table><tr><td></td><td>Local provider</td><td>Expatriate provider</td></tr><tr><td rowspan="4">Physician anaesthesiologist (PA) led</td><td>PA</td><td>none</td></tr><tr><td>NA</td><td>PA</td></tr><tr><td>UA</td><td>PA</td></tr><tr><td>None</td><td>PA</td></tr><tr><td rowspan="3">Nurse anaesthetist (NA) led</td><td>NA</td><td>none</td></tr><tr><td>UA</td><td>NA</td></tr><tr><td>None</td><td>NA</td></tr><tr><td>Uncertified anaesthetic provider (UA) led</td><td>UA</td><td>None</td></tr></table></td><td>Programme-level routine data (MT)</td></tr>
<tr><td>Setting</td><td>Type of care provided by hospital</td><td>Categorical (nominal)</td><td>Overall type of provision provided during surgical project, based on MSF-OCB brief. 9 separate categories grouped into 5:
- Emergency only
- Capacity to perform both emergency and elective surgery
- Maternity care only
- Trauma care only
- Other specific care provision (wound care, trauma and surgical care, obstetric fistula care, and surgical care of typhoid related complications)</td><td>Programme-level routine data (MT)</td></tr>
</table>

	WHO region	Categorical (nominal)	The location of each mission was labelled according to the region codes used by the World Health Organisation:<br>- AFR<br>- EMR<br>- SEAR<br>- AMR<br>- WPR	WHO
	Setting	Categorical (binary)	3 variables:<br>- Conflict<br>- Natural disaster<br>- Health care gaps	Programme-level routine data (MT)
	Hospital level	Ordinal (Categorical)	4 distinct categories as per MSF-OCB surgical policy guidelines:<br>- Sole remit hospital = hospital that provides care for a specific purpose (i.e. not necessarily a quaternary referral hospital, but a surgical setup for a specific indication). Examples include: Trauma centre, Maternity centre, Fistula repair camp.<br>- Referral hospital = provincial hospital, considered tertiary referral hospital.<br>- District hospital = can manage most, but will refer complex cases on to referral hospitals.<br>- Health centres = small rural health centres with capacity to perform basic surgical operations	Programme-level routine data (MT)
	Site ID	Character	Anonymous unique code for each site	produced in R 3.6

Appendix Table 2: surgical projects in health care gap settings in the WHO SEA region (2 in total)

**Type of anaesthesia provider**	Physician anaesthetist only
**Type of hospital**	- District hospital<br>- Health centre
**Type of care provided by surgical project (No. of projects)**	- Capacity to perform both emergency and elective surgery<br>- Filiariasis-related care
**Total duration, months**	19
**Type of surgical provider for entire duration**	- General & specialty surgeons<br>- General surgeon only
**Total number of all cases, No.**	815
**Main cause for hospitalisation, No. (%)**	- Other, 482 (59)
**Main category of surgery, No. (%)**	- Minor Surgery, 507 (62)
**Intra-operative mortality, No. (%)**	2 (0.2)

Appendix Table 3: Surgical projects in natural disaster settings (5 in total)

**Type of anaesthesia provider**	Physician anaesthetist only
**Type of hospital**	- District<br>- Sole remit hospital
**Type of care provided by surgical project (No. of projects)**	- Capacity to perform both emergency and elective surgery (2)<br>- Trauma care only (1)<br>- Emergency only (2)
**Total duration, months**	40
**Presence of surgical provider, months**	
- **General and specialty surgeons**	23
- **General surgeon only**	16
- **Specialty surgeon only**	1
**Total number of all cases, No. (%)**	3108
**Main cause for hospitalisation, No. (%)**	- Other, 1144 (37)
**Main category of surgery, No. (%)**	- Minor surgery, 1608 (52)
**Intra-operative mortality, No. (%)**	9 (0.3)

Appendix Table 4: Surgical groupings as used in main table 2 (case-level data)

Surgical grouping	Examples of types of surgery included
**Minor surgery**	• Simple wound treatment<br>• Insertion/removal of drain<br>• Burns dressing change<br>• Wound debridement<br>• Removal of foreign body<br>• Amputation of digits or toes<br><br>Incl. procedure codes with median operative time < 45min within the dataset:<br><br>• Curettage post delivery (GP)<br>• Reduction of fractures (OR)<br>• Removal of osteosynthesis (OX)<br>• Ophthalmic surgery (SO)
**Caesarean section**	• Caesarean section only
**Visceral surgery**	• Exploratory laparotomy<br>• Hernia repair<br>• Resection/repair solid organs (e.g. spleen/liver) or gut
**Orthopaedic surgery**	• External or internal fixation of fracture<br>• Surgery to any joint<br>• Limb amputation (excluding digits or toes)<br>• Curettage for osteomyelitis
**Obstetric & gynaecological surgery (excl. Caesarean section)**	• Management of ectopic pregnancy<br>• Obstetric fistula repair<br>• Hysterectomy
**Specialties**	• Urology<br>• Vascular surgery<br>• Plastic and reconstructive surgery<br>• Ear, nose and throat surgery<br>• Neurosurgery<br>• Thoracic surgery<br>• Maxillofacial surgery<br>• Other specialized surgery

Modified from the original surgical groupings outlined in the "MSF-OCB Operating Department Data Collection Guidelines (2015)".

Missing data Table 1: Number of missing values within each variable

Variable	Number missing
**ASA**	3232
**Intra-operative mortality**	2154
**Time in theatre**	1922
**Age**	47
**Main procedure**	5
**Choice of anaesthesia**	2
**Gender**	0
**Date of procedure**	0
**Cause of hospitalisation**	0
**Urgency**	0
**Order**	0
**Surgical provider**	0
**Anaesthesia provider**	0
**Who region**	0
**Setting**	0
**Type of hospital**	0
**Type of care provided**	0
**Site ID**	0

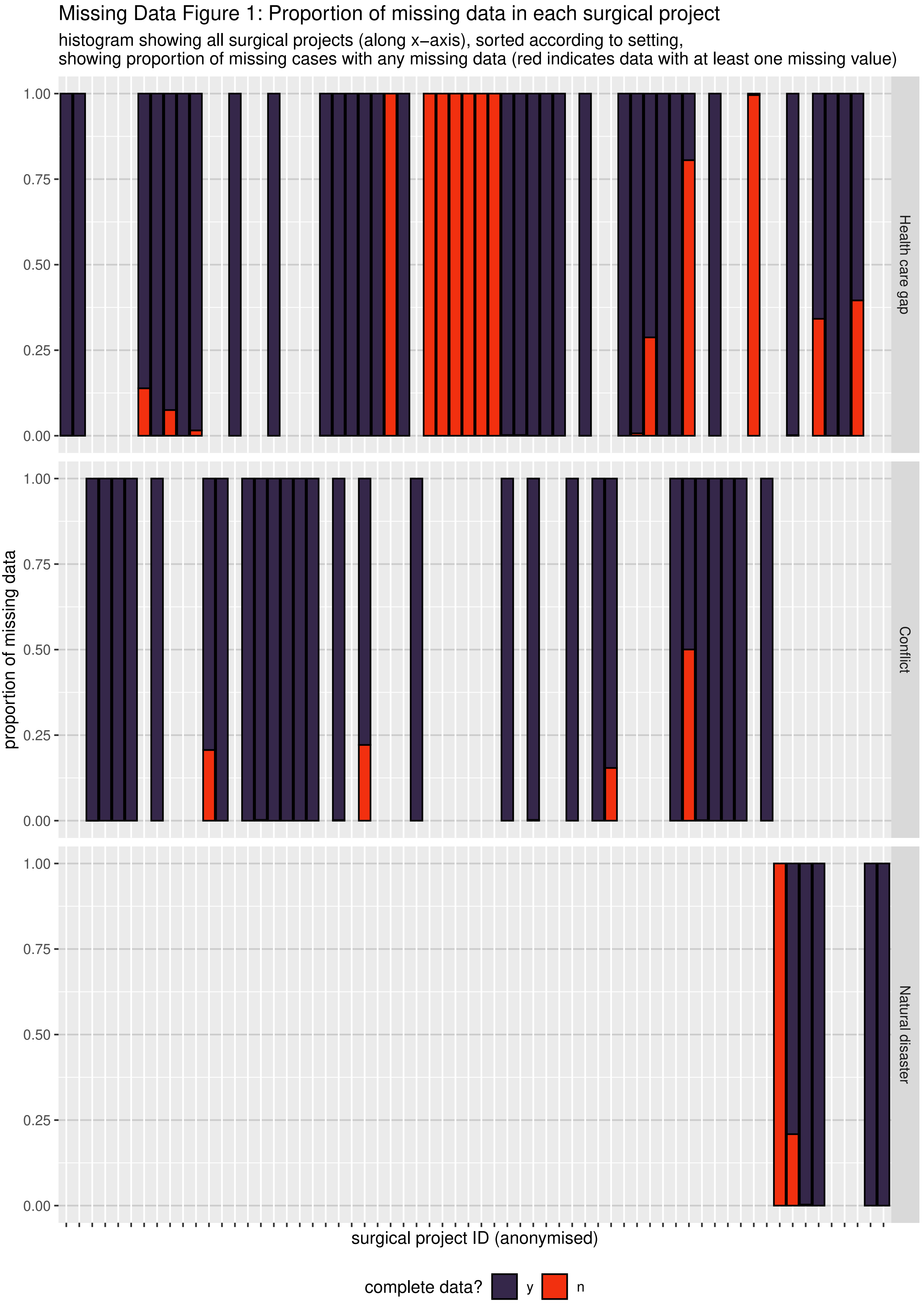
Missing Data Figure 1: Proportion of missing data in each surgical project
histogram showing all surgical projects (along x−axis), sorted according to setting,
showing proportion of missing cases with any missing data (red indicates data with at least one missing value)
proportion of missing data
1.00
0.75
0.50
0.25
0.00
Health care gap
Conflict
Natural disaster
surgical project ID (anonymised)
complete data?
y
n

Missing data Table 2: overview of available data

Variable	Value
**Median age, years (IQR)**	26 (18-36)
**Female, no. (%)**	2963 (52)
**Median ASA (IQR)**	1 (1-2)
**Emergent surgery, no. (%)**	2277 (40)
**Cause for hospitalisation, no. (%)**	
- **Obstetric**	1188
- **Any trauma**	2074
- **Other**	2468
**Main surgical procedure**	
- **Minor surgery**	2854
- **Caesarean section**	746
- **Visceral surgery**	1408
- **Obstetric and gynaecology**	503
- **Orthopaedics**	177
- **Other specialty surgery**	37
**Intraoperative mortality**	
- **Alive**	3563
- **Died**	13
**Median theatre time, minutes (IQR)**	45 (35-60)
**Setting**	
- **Health care gap**	3359
- **Conflict**	1443
- **Natural disaster**	928
