# Supplementary material for: Anaesthesia care providers employed in humanitarian settings by Médecins Sans Frontières: a retrospective observational study of 173 084 surgical cases over 10 years
Source: BMJ Open. 2020 Mar 4;10(3):e034891. doi: 10.1136/bmjopen-2019-034891 (PMC7059447; doi:10.1136/bmjopen-2019-034891)
Supplement: Author's manuscript [file bmjopen-2019-034891.draft_revisions.pdf]

BMJ Open

BMJ Open is committed to open peer review. As part of this commitment we make the peer review history of every article we publish publicly available.

When an article is published we post the peer reviewers' comments and the authors' responses online. We also post the versions of the paper that were used during peer review. These are the versions that the peer review comments apply to.

The versions of the paper that follow are the versions that were submitted during the peer review process. They are not the versions of record or the final published versions. They should not be cited or distributed as the published version of this manuscript.

BMJ Open is an open access journal and the full, final, typeset and author-corrected version of record of the manuscript is available on our site with no access controls, subscription charges or pay-per-view fees (http://bmjopen.bmj.com).

If you have any questions on BMJ Open's open peer review process please email info.bmjopen@bmj.com

BMJ Open

# The use of different anaesthesia providers in humanitarian settings: Descriptive study of 173,084 episodes of surgical care provided by Médecins Sans Frontières over 10 years

| | |
|---|---|
| Journal: | *BMJ Open* |
| Manuscript ID | bmjopen-2019-034891 |
| Article Type: | Original research |
| Date Submitted by the Author: | 10-Oct-2019 |
| Complete List of Authors: | Kudsk-Iversen, Soren; Oxford University Hospitals NHS Foundation Trust, Nuffield Department of Anaesthetics<br>Trelles, Miguel; Medecins Sans Frontieres<br>Ngowa Bakebaanitsa, Elie; Medecins Sans Frontieres; Masisi referral hospital, Masisi – MSF Democratic Republic of the Congo mission<br>Hagabimana, Longin; Medecins Sans Frontieres; Arche trauma hospital, Bujumbura – MSF Burundi mission<br>Momen, Abdul; Medecins Sans Frontieres; Khost maternity, Khost – MSF Afghanistan mission<br>Helmand, Rahmatullah; Medecins Sans Frontieres; Ahmad Shah Baba hospital, Kabul – MSF Afghanistan mission<br>Saint Victor, Carline; Medecins Sans Frontieres; Tabarre trauma hospital, Port-au-Prince – MSF Haiti mission<br>Shah, Khalid; Medecins Sans Frontieres; Timurgara district headquarter hospital – MSF Pakistan mission<br>Masu, Adolphe; Medecins Sans Frontieres; Castors maternity, Bangui – MSF Central African Republic mission<br>Kendell, Judith; Medecins Sans Frontieres<br>Edgcombe, Hilary; Oxford University Hospitals NHS Foundation Trust, Nuffield Department of Anaesthetics<br>English, Mike; KEMRI - Wellcome Trust Research Programme, Health Services Unit; University of Oxford Centre for Tropical Medicine and Global Health |
| Keywords: | ANAESTHETICS, Human resource management < HEALTH SERVICES ADMINISTRATION & MANAGEMENT, Organisation of health services < HEALTH SERVICES ADMINISTRATION & MANAGEMENT |
| | |

SCHOLARONE™
Manuscripts

BMJ

*I, the Submitting Author has the right to grant and does grant on behalf of all authors of the Work (as defined in the below author licence), an exclusive licence and/or a non-exclusive licence for contributions from authors who are: i) UK Crown employees; ii) where BMJ has agreed a CC-BY licence shall apply, and/or iii) in accordance with the terms applicable for US Federal Government officers or employees acting as part of their official duties; on a worldwide, perpetual, irrevocable, royalty-free basis to BMJ Publishing Group Ltd ("BMJ") its licensees and where the relevant Journal is co-owned by BMJ to the co-owners of the Journal, to publish the Work in this journal and any other BMJ products and to exploit all rights, as set out in our licence.*

*The Submitting Author accepts and understands that any supply made under these terms is made by BMJ to the Submitting Author unless you are acting as an employee on behalf of your employer or a postgraduate student of an affiliated institution which is paying any applicable article publishing charge ("APC") for Open Access articles. Where the Submitting Author wishes to make the Work available on an Open Access basis (and intends to pay the relevant APC), the terms of reuse of such Open Access shall be governed by a Creative Commons licence – details of these licences and which Creative Commons licence will apply to this Work are set out in our licence referred to above.*

*Other than as permitted in any relevant BMJ Author's Self Archiving Policies, I confirm this Work has not been accepted for publication elsewhere, is not being considered for publication elsewhere and does not duplicate material already published. I confirm all authors consent to publication of this Work and authorise the granting of this licence.*

For peer review only

# The use of different anaesthesia providers in humanitarian settings: Descriptive study of 173,084 episodes of surgical care provided by Médecins Sans Frontières over 10 years

Søren Kudsk-Iversen, MBChB DTM&H FRCA
Address:
Stoke Mandeville Hospital, Mandeville Rd, Aylesbury HP21 8AL, UK

Affiliation:
(1) Nuffield Department of Anaesthetics, Oxford University Hospitals NHS Trust, Oxford, UK

*Corresponding author: sorenki@gmail.com, +44(0)7707838060

Miguel Trelles, PhD
Address:
Rue de l'Arbre Benit 46, 1050 Bruxelles, Belgique

Affiliation:
(1) Médecins sans Frontières
(2) Medical Department – Operational Centre Brussels

Elie Ngowa Bakebaanitsa, Diplôme de Anesthésie et Réanimation
Address:
l'Hôpital général de reference de masisi, zone de santé de masisi, division provinciale de santé du nord-kivu, Republique Democratique du Congo

Affiliation:
(1) Médecins sans Frontières
(2) Masisi referral hospital, Masisi – MSF Democratic Republic of the Congo mission

Longin Hagabimana, Diplôme de Licence en Anesthésie-Réanimation
Address:
L'Hôpital Arche Médical de Kigobe, Bujumbura, Burundi

Affiliation:
(1) Médecins sans Frontières
(2) Arche trauma hospital, Bujumbura – MSF Burundi mission

Abdul Momen, MD
Address:
Khost Maternity Hospital, Khost, Afghanistan

Affiliation:
(1) Médecins sans Frontières
(2) Khost maternity, Khost – MSF Afghanistan mission


For peer review only - http://bmjopen.bmj.com/site/about/guidelines.xhtml

Rahmatullah Helmand, MD
Address:
Jamhoriat Hospital, Chackin Street, New City, Kabul, Afganistan

Affiliation:
(1) Médecins sans Frontières
(2) Ahmad Shah Baba hospital, Kabul – MSF Afghanistan mission

Carline Saint Victor, Certificat en soins anesthésiques et réanimation
Address:
Maternité Isai Jeanty (Hopital Chancerelles), Boulevard Jean-Jacques Dessalines, Port-au-Prince, Haiti

Affiliation:
(1) Médecins sans Frontières
(2) Tabarre trauma hospital, Port-au-Prince – MSF Haiti mission

Khalid Shah, Post RN Dip (Anesthesia)
Address:
DHQ Hospital Timergara, Lower Dir, Khyber Pakhtunkhwa, Pakistan

Affiliations:
(1) Médecins sans Frontières
(2) Timurgara district headquarter hospital – MSF Pakistan mission

Adolphe Masu, MMed
Address:
Avenue Tilipiers number 30, Quartier les Volcans, Ville de Goma, North Kivu province, Republique Democratique du Congo

Affiliations:
(1) Médecins sans Frontières
(2) Arche trauma hospital, Bujumbura – MSF Burundi mission
(3) Khost maternity, Khost – MSF Afghanistan mission
(4) Castors maternity, Bangui – MSF Central African Republic mission

Judith Kendell, MBBS
Address:
Médecins sans Frontières, Rue de l'Arbre Bénit 46, 1050 Bruxelles, Belgique

Affiliation:
(1) Médecins sans Frontières
(2) Medical Department – Operational Centre Brussels

For peer review only

Hilary Edgcombe, BM BCh FRCA
Address:
Nuffield Department of Anaesthetics, Level 2, John Radcliffe Hospital, Headley Way OX3 9DU

Affiliation:
(1) Nuffield Department of Anaesthetics, Oxford University Hospitals NHS Trust, Oxford, UK

Mike English, FMedSci
Address:
Centre for Tropical Medicine and Global Health, University of Oxford, Old Road Campus, Roosevelt Drive, Oxford OX3 7FZ, UK

Affiliation:
(1) KEMRI-Wellcome Trust Research Programme, Nairobi, Kenya
(2) Centre for Tropical Medicine and Global Health, University of Oxford, Oxford, UK

**Key words:**

Global health
Humanitarian aid
Anaesthesia
Task-shifting and sharing

Word Count (excluding title page, abstract, references, tables, figures): 3106 words

For peer review only

## Abstract

**Objective:**

To characterize the volume and nature of the surgical workload and, specifically, to explore the nature and extent of the use of different categories of anaesthesia providers.

**Design:**

This is a descriptive analysis, using 10 years (2008-2017) of routinely collected case-level data linked with routine program-level data from surgical projects exclusively run by MSF-Operational Centre Brussels (MSF-OCB).

**Setting:**

Surgical projects were situated in contexts of natural disaster (ND, where MSF-OCB deploy an entire expatriate surgical team), active conflict (AC) and stable health care gaps (HG). In both AC and HG settings, MSF-OCB support pre-existing local facilities. The hospital facilities ranged from basic health centres with surgical capabilities to tertiary referral settings.

**Participants:**

Anaesthesia providers in MSF-OCB structures can be categorised according to their level of training: Physician anaesthetists (PAs), qualified nurse anaesthetists (NAs), and unqualified anaesthesia providers (UAs).

**Primary and Secondary Outcome Measures:**

Surgical volume and nature of surgical cases.

**Results:**

Across the three distinct settings a total of 173,084 surgical cases (2,518 in ND, 42,225 in AC, 126,936 in HG) had full routine data collected (96.8% of all cases). Anaesthesia was predominantly led by PAs (100% in ND, 66% in AC and HG), then by NAs (19% in AC and HG), or UAs (15% in AC and HG). Across all settings and provider groups, patients were mostly healthy young adults (median 24-27 years) with predominantly female patients (including those undergoing caesarean sections) in HG contexts, and male cases in AC contexts. Intra-operative mortality was low.

**Conclusion:**

We demonstrate the value of collecting high quality, routine data at scale in the humanitarian sector, a sector with considerable experience in task sharing and shifting, which can inform global debates on provision of anaesthesia. Further work is needed to evaluate the outcomes of task-shifted anaesthesia within the humanitarian space.




For peer review only - http://bmjopen.bmj.com/site/about/guidelines.xhtml

**Article Summary**

**Strengths and limitations of this study**

- This is the largest study detailing how anaesthetic task sharing and shifting is employed in the humanitarian sector
- Additionally, we believe this is the first study to describe the extent of the presence and caseload of unqualified anaesthetic providers in humanitarian surgical projects
- As the study makes use of linked routinely collected surgical surveillance data, we have tried to limit the risk of selection bias through missingness analysis
- Due to the nature of the linked data, we were unable to connect anaesthetic provider with individual operations. Therefore, to limit the misclassification bias, we do not ascribe a provider to each case, but rather describe the most senior provider present in the surgical project (the 'anaesthetic lead')

For peer review only


For peer review only - http://bmjopen.bmj.com/site/about/guidelines.xhtml

## INTRODUCTION

Globally there is a large unmet surgical need. Low and middle-income countries (LMIC) are disproportionately affected by gaps in health care provision, with an estimated 90% of patients in these countries unable to access basic surgical care.[1] The burden is increased and access further reduced in crisis situations, caused by conflict or natural disasters.[2] To address these imbalances, Médecins Sans Frontières (MSF, also known as Doctors without Borders) provide humanitarian surgical assistance based on the needs of affected populations through one or more of their five operational centres, one of which is Operational Centre Brussels (MSF-OCB).

There is an increasing body of literature outlining the surgical needs of populations in humanitarian settings;[3–6] further, the recognition that the humanitarian sector is not immune from the need to demonstrate safe surgical care has led to calls for more robust outcome data and clearer accountability.[7–9] However, only few studies, limited by small study size and limited external validity, have addressed the composition of the surgical workforce employed by humanitarian organizations.[10,11] As such, there is inadequate published data on whether different anaesthesia providers (e.g. physician, nurse, or other health care provider) are employed in different settings, and to what extent there is a physician expatriate presence within the team. In order to comment on outcomes and identify areas where practice can be improved, it is essential to know who provides the care and if there is any learning that can be derived from their practice.

Therefore, the objective of this study is to characterise the volume and nature of the surgical workload and, specifically, to explore the nature and extent of the use of different categories of anaesthesia providers.

## METHODS

The study protocol was submitted to the Oxford Tropical Research Ethics Committee who granted ethical exemption. The study also fulfilled the exemption criteria set by the MSF Ethics Review Board for *a posteriori* analyses of routinely collected clinical data and thus did not require MSF ERB review. It was conducted with permission from Medical Director, MSF-OCB. This exemption did not allow country/site specific detail to be included, therefore we aggregate data within the World Health Organization (WHO) regional groupings.[12] The findings are reported in accordance with RECORD, the extended STROBE statement on routinely collected data.[13]

### Study design

This was a descriptive study of routine data collected between January 2008 and December 2017. We excluded any incomplete data and data from surgical projects where MSF-OCB were collaborating with other MSF operational centres or local governments, as we were unable to account for workforce or resources made available by others than MSF-OCB.

We linked three sources of data (see figure 1). 1) Case-level routine surgical surveillance data were recorded by theatre staff in logbooks on-site, then transcribed onto an Excel spreadsheet, and finally transferred to Brussels on a monthly basis where they were reviewed and any missing or extraneous data was queried with the local teams. 2) Program-level data, available from MT (head of the Surgical, Anaesthesia, Gynaecology, and Emergency Medicine unit during this period) were reviewed. 3) End of deployment reports written by expatriate physician anaesthetists were reviewed to fill gaps in data from the case-level data.



For peer review only - http://bmjopen.bmj.com/site/about/guidelines.xhtml

Data were de-identified at point of data collection, and were only accessed by SK, MT and JK. Any data shared with the remaining co-authors were fully anonymized.

**Data sharing statement**

All data used in the study are presented in the tables and appendices. No additional data are available.

**Setting and anaesthesia providers**

Three different project setting types were identified: 1) regions recently affected by sudden onset natural disasters (ND) where MSF deployed an entire expatriate surgical team in accordance with WHO minimum requirements,[14] 2) active armed conflict (AC) situations and 3) stable situations where MSF supported a pre-existing local facility to address health-care gaps (HG), which existed for a variety of reasons, including the aftermath of natural disasters or armed conflict.

The setup and duration of surgical projects varied. Some projects were intended to operate only for a short period, either within existing local infrastructure or through fully self-contained surgical platforms. Other projects were set up to serve for a longer period or evolved over time into a fully functioning hospital with ability to provide complex care provision. The different hospital types are described in detail in the appendix (appendix 1). The setup was not dictated by the setting, and could change over the course of a project. During the 10 years studied, anaesthesia provision was led by one of the following: a) physician anaesthetists (PAs), either local or expatriate doctors with specialist qualifications in anaesthesia, b) nurse anaesthetists (NAs), either local or expatriate nurses or other non-physician clinical cadres with formal training and qualification in anaesthesia in their country of origin, or c) unqualified anaesthesia providers (UAs), local nurses or allied health care professionals with a broad range of different levels of experience in anaesthesia provision but without a formal qualification who received on-the-job training only. The MSF-OCB anaesthesia referent assesses the provider requirement for each location based on suspected workload, job description and staff availability. For example, if a project is suspected to have a low workload, NAs are recruited locally (or sent as expatriates if they are senior providers). However, in situations where MSF-OCB are unable to source qualified staff for a surgical project, they may hire the existing local UAs, who will all receive on-the-job training by MSF and supervision by expatriate PAs for a trial period. These situations should result in UAs working in settings with a low workload and with distant supervision available from a nearby hospital with MSF-OCB involvement where anaesthesia is led by an expatriate PA. All MSF surgical projects have standardized anaesthetic equipment and medications, as described elsewhere.[5]

**Variables and bias**

Different variables were retrieved from the three different data sources. From the routine case-level data (and end of deployment reports) we identified patient variables (including age and sex), surgical and anaesthetic variables (including type of surgery, type of anaesthesia), and geographic location of the cases done. From the program-level data we obtained additional surgical and anaesthetic variables (including provider level of training, presence of expatriate), and location variables (including project setting, type of hospital). A detailed description of all variables used is available in the appendix, table 1.


For peer review only - http://bmjopen.bmj.com/site/about/guidelines.xhtml

The use of routine surveillance data puts the study at risk of selection bias, which may risk under-reporting by some providers (e.g. expatriates visiting for short periods who may be unfamiliar with the data collection tool, or staff who for whatever reason choose not to document cases) or in busy settings (e.g. high workload or strained workforce). While we cannot account for surgical cases not recorded in the first place, we therefore explored data excluded due to incompleteness to assess similarity to the included data.
Furthermore, it should be noted that provider data were available showing the most senior provider present for each project, not per case (and for expatriates, was updated monthly during a project). This puts the study at risk of misclassification bias regarding the anaesthesia providers in favour of the most senior team member regardless of their presence in theatre. Additionally, it would be easy to overrepresent the case-level involvement of PAs (especially when expatriate as they might be more restricted in their movement and have additional non-clinical commitments). We therefore present data according to the most senior provider present on the project in a given month (the anaesthetic 'lead'). We also note which projects had a visiting expatriate PA present (figure 3B).

**Statistical analysis**
Data were collected and linked in Excel (2016) and data cleaning and analysis was performed in R 3.6. Continuous data were assessed for normality, and no parametric data were identified. For non-parametric continuous and numeric ordinal data, median, interquartile (IQR) and full range were reported. For categorical variables, the raw counts were reported. We stratified our analysis according to the settings identified, as they might influence the extent and pattern by which different anaesthetic providers were deployed.
However, data from surgical projects in the WHO South East Asia region and in ND settings were described separately due to their small numbers and being separate from the dominant regions (see appendix, tables 2 and 3).

**Patient and public involvement**
There was no involvement of patients or the public in the development or execution of this study.

**Funding**
This research received no specific grant from any funding agency in the public, commercial or not-for-profit sectors.

## RESULTS

**General findings**
Over the 10 years a total of 173,084 cases had full routine data collected (96.8% of all cases) across 23 countries and 52 different locations (see figure 1). The majority of cases occurred in HG settings, and in the WHO Africa region (see figure 2B). Surgical projects in settings of ND represented 2,518 cases (less than 2% of the total number of operations over the time period) and a total duration of 40 project-months over 5 sites; anaesthesia care in the ND setting was exclusively led by PAs (see appendix, table 3).


For peer review only - http://bmjopen.bmj.com/site/about/guidelines.xhtml

Overall the shortest surgical project lasted a month, and the longest lasted beyond the 10 years covered by this study (see figure 3). Surgical projects in HG settings stayed open for longer (median 866 days, IQR 360.25-1900 days) than projects in AC and ND settings (287.5, 173-498.25 days and 210, 122-308 days, respectively). The workload within each project varied widely, with 31 projects accounting for 5.1% of all cases, and four projects accounting for 47.6% (see figure 3A).

Of the four biggest projects, anaesthesia for two projects was exclusively PA-led (one in the WHO Eastern Mediterranean region in an AC setting, the other in the WHO Americas region in a HG setting). The third project was predominantly PA-led (in the WHO Eastern Mediterranean region) progressing from an initial AC to become a stable HG setting. The last was predominantly UA-led with a periodic presence of expatriate PAs (in the WHO Africa region, starting in AC and then becoming a stable HG setting). Data for these four major projects followed a similar pattern of distribution (in terms of case and program-level data) to the remaining dataset of all other projects, and have therefore been included in the findings below.

**Program-level provider findings**

Most surgical projects (23/28 in AC, 25/32 in HG, and all 5 in ND) included a period of anaesthesia provision led by PAs (see figure 3B and table 1A). Anaesthesia in any setting with sole trauma care was mostly led by PAs (see table 1A). If anaesthesia provision in a project was not fully PA-led, the pattern of PA presence in most cases involved short periods (usually around 3 months) over the course of the surgical project, mostly towards the start of the project (see figure 3B).  Overall, a PA was identified as present for 737 (49%) project-months in AC and HG (see table 1B). However, in these settings more than 66% of cases overall were conducted during PA-led anaesthesia periods (80% of cases in AC and 60% of cases in HG).

When there was not a PA attached to a project, anaesthesia was most commonly led by NAs in the HG setting and most commonly led by UAs in the AC setting.


For peer review only - http://bmjopen.bmj.com/site/about/guidelines.xhtml

**Table 1A: Programme-level descriptive table according to number of surgical projects in different settings**

| | Physician anaesthetist led | Nurse anaesthetist led | Unqualified anaesthetic provider led | Physician anaesthetist led | Nurse anaesthetist led | Unqualified anaesthetic provider led |
|---|---|---|---|---|---|---|
| | CONFLICT (SURGICAL PROJECTS = 28) | | | HEALTH CARE GAP (SURGICAL PROJECTS = 32)[a] | | |
| **Number of surgical projects involved in at any point[b]** | 23 | 14 | 12 | 25 | 17 | 12 |
| **Type of hospital[c] in surgical project, No. surgical projects involved in at any point[d]** | | | | | | |
| **Sole remit hospital** | 5 | 2 | 1 | 13 | 7 | 1 |
| **Referral hospital** | 6 | 5 | 2 | 7 | 5 | 2 |
| **District hospital** | 11 | 4 | 8 | 7 | 4 | 7 |
| **Health centre** | 1 | 3 | 1 | 0 | 1 | 2 |
| **Type of surgical care performed in project, No. surgical projects involved in at any point[d]** | | | | | | |
| **Emergency only** | 9 | 8 | 5 | 3 | 4 | 4 |
| **Capacity to perform both emergency and elective surgery** | 8 | 4 | 6 | 11 | 5 | 6 |
| **Maternity care only** | 1 | 1 | 1 | 9 | 6 | 1 |
| **Trauma care only** | 4 | 1 | 0 | 2 | 0 | 0 |
| **Other specific care provision[e]** | 1[1] | 0 | 0 | 2[2,3] | 1[3] | 1[4] |



For peer review only - http://bmjopen.bmj.com/site/about/guidelines.xhtml

Table 1B: Programme-level descriptive table according to months of activity in different settings

| | Physician anaesthetist led | Nurse anaesthetist led | Unqualified anaesthetic provider led | All conflict missions | Physician anaesthetist led | Nurse anaesthetist led | Unqualified anaesthetic provider led | All health care gap missions |
|---|---|---|---|---|---|---|---|---|
| | CONFLICT (SURGICAL PROJECTS = 28) | | | | HEALTH CARE GAP (SURGICAL PROJECTS = 32)[a] | | | |
| **Number of months active in any mission** | 235 | 75 | 94 | **404** | 502 | 429 | 160 | **1091** |
| **Surgical provider, No. months present (% of cohort)** | | | | | | | | |
| **General and specialty surgeon** | 100 (43) | 13 (17) | 0 (0) | **113 (28)** | 260 (52) | 105 (24) | 3 (2) | **368 (34)** |
| **General surgeon only** | 96 (41) | 58 (77) | 20 (20) | **174 (43)** | 115 (23) | 133 (31) | 38 (24) | **286 (26)** |
| **Specialty surgeon only** | 31 (13) | 2 (3) | 4 (4) | **37 (9)** | 103 (21) | 162 (38) | 7 (4) | **272 (25)** |
| **MD[f]** | 8 (3) | 2 (3) | 70 (74) | **80 (20)** | 24 (5) | 29 (7) | 112 (70) | **165 (15)** |

[a] South East Asia Region contributed such a small proportion to missions in "health care gap" settings (2 missions, 815 cases or <1%), that they have been excluded and instead described in appendix, table 2.

[b] Surgical projects can have anaesthesia provision by multiple different providers during the period they are open. Therefore, the rows might add up to more than the total number of projects in each setting.

[c] Definitions of hospitals found in appendix, table 1.

[d] Two surgical projects changed from being able to provide both emergency and elective surgery, to providing solely maternity care. As such, they are counted twice under "type of hospital" and "type of surgical care".

[e] Specific care provision are surgical projects with a specific care remit. This includes [1]wound care, [2]trauma and surgical care, [3]obstetric fistula care, and [4]surgical care of typhoid related complications.

[f] MD = local physician with surgical skills but without a formal surgical qualification.



For peer review only - http://bmjopen.bmj.com/site/about/guidelines.xhtml

**Case-level provider findings**

Case-mix was similar across all lead providers with respect to age (mostly young adults) and underlying health (mostly ASA 1) (see table 2). All providers did predominantly non-elective work, although cases done during NA-led project-months had a higher proportion of emergency surgery. During NA-led project-months there was a predominance of caesarean sections in HG settings, though outside this setting the most common type of surgery was minor surgery for all lead providers. The intra-operative mortality was 0.3% and 0.3% in PA-led project-months, 0.2% and 0.1% in NA-led project-months, and 0.3% and 0.2% in UA-led project-months in AC and HG settings, respectively.

All lead providers made use of the two most common types of anaesthesia: spinal injection alone and ketamine-based general anaesthesia (GA) without a protected airway. Furthermore, this was done in broadly similar proportions when comparing surgical categories in different settings (spinal injection and GA without protected airway for caesarean section was 61-70% and 22-36%, respectively in AC, and 78-86% and 6-14%, respectively in HG).

For peer review only


For peer review only - http://bmjopen.bmj.com/site/about/guidelines.xhtml

**Table 2: Case-level descriptive table grouped according to setting[a]**

| | Physician anaesthetist led | Nurse anaesthetist led | Unqualified anaesthetic provider led | Physician anaesthetist led | Nurse anaesthetist led | Unqualified anaesthetic provider led |
|---|---|---|---|---|---|---|
| | **CONFLICT (Surgical Projects = 28, N = 42,225)** | | | **HEALTH CARE GAP (Surgical Projects = 32, N = 126,936)[b]** | | |
| **Number of all surgical episodes, No.** | 33763 | 3798 | 4664 | 78126 | 28559 | 20251 |
| **Patient demographics** | | | | | | |
| **Female, No. (%)** | 12424 (37) | 1888 (50) | 2237 (48) | 38919 (50) | 22439 (79) | 12834 (63) |
| **Median age, years (IQR, [range])** | 23 (15-33, [1 day old-105]) | 25 (16-34, [2 day old-90]) | 23 (18-30, [3 day old-94]) | 28 (19-37, [1 day old-102]) | 26 (20-34, [1 day old-98]) | 25 (16-35, [1 day old-96]) |
| **ASA, value (IQR, [range])** | 1 (1-2, [1-5]) | 2 (1-2, [1-5]) | 1 (1-2, [1-5]) | 1 (1-2, [1-5]) | 1 (1-2, [1-5]) | 1 (1-2, [1-5]) |
| **Cause of hospitalisation, No. %:** | | | | | | |
| • **Trauma (intentional or unintentional)** | 21968 (65) | 1384 (36) | 2366 (51) | 42454 (54) | 2850 (10) | 6303 (31) |
| • **Obstetric** | 6642 (20) | 1073 (28) | 1295 (28) | 20387 (26) | 18270 (64) | 7211 (36) |
| • **Other[c]** | 5153 (15) | 1341 (35) | 1003 (22) | 15285 (20) | 7439 (26) | 6737 (33) |
| **Surgical demographics** | | | | | | |
| **Urgency, No. (%)** | | | | | | |
| • **Emergent** | 14344 (42) | 2203 (58) | 2581 (55) | 37234 (48) | 19922 (70) | 9221 (46) |
| • **Urgent** | 18091 (54) | 1115 (29) | 1961 (42) | 34771 (45) | 4781 (17) | 8699 (43) |
| • **Elective** | 1328 (4) | 480 (13) | 122 (3) | 6121 (8) | 3856 (14) | 2331 (12) |
| **Proportion of cases from initial presentation, n (%)** | 20079 (59) | 3053 (80) | 3588 (77) | 51493 (66) | 25597 (90) | 14492 (72) |
| **Median time in theatre, minutes (IQR, [range])** | 50 (30-70, [7-710]) | 50 (35-70, [15-356]) | 45 (35-65, [10-360]) | 60 (35-90, [10-870]) | 60 (50-80, [10-1140]) | 50 (30-70, [5-460]) |
| **Main categories of surgery, No. (%)[d]** | | | | | | |
| **Minor surgery** | 20670 (61) | 1688 (44) | 3019 (65) | 37419 (48) | 6798 (24) | 9906 (49) |
| **Caesarean section** | 4758 (14) | 891 (23) | 884 (19) | 16138 (21) | 13336 (47) | 6259 (31) |

| | | | | | | |
|---|---|---|---|---|---|---|
| **Visceral surgery** | 3709 (11) | 949 (25) | 555 (12) | 11109 (14) | 4856 (17) | 2922 (14) |
| **Orthopaedic surgery** | 3372 (10) | 90 (2) | 48 (1) | 9408 (12) | 151 (1) | 328 (2) |
| **Obstetric & gynaecological surgery (excl. caesarean section)** | 802 (2) | 122 (3) | 139 (3) | 3226 (4) | 3147 (11) | 756 (4) |
| **Specialties[e]** | 452 (1) | 58 (2) | 19 (0) | 826 (1) | 271 (1) | 80 (0) |
| **Intra-operative mortality, No. (%)** | | | | | | |
| **For all cases** | 102 (0·3) | 7 (0·2) | 16 (0·3) | 204 (0·3) | 31 (0·1) | 31 (0·2) |

[a] Percentages have been rounded to nearest full digit, and might not add up to 100%.

[b] South East Asia Region contributed such a small proportion to missions in "health care gap" settings (2 missions, 815 cases or <1%), that they have been excluded and instead described in the appendix, table 2.

[c] "Other" causes of hospitalisation include: tropical disease related, tumours, non-tumour related obstruction, and complications from traditional medical practices.

[d] The surgical procedures included in each grouping can be found in the appendix, table 1.

[e] Specialties encompass (total number of cases across whole dataset): Urology (726), vascular surgery (355), plastic and reconstructive surgery (144), ENT surgery (116), neurosurgery (115), surgery within thoracic cavity (108), maxillofacial surgery (61), and other forms of specialised surgical care that does not fall into the aforementioned categories (109).



For peer review only - http://bmjopen.bmj.com/site/about/guidelines.xhtml

**Missingness analysis**
The cases excluded (5730, 3.3%) due to missing variables (see appendix, missingness analysis table 1) are predominantly from the early years (see appendix, missingness analysis figure 1). Eight surgical projects were completely excluded (7 in health care gap settings, 1 that was in both natural disaster settings and health care gap settings, see appendix, missingness analysis figure 2), and were predominantly in projects with UA-led or PA expatriate led provision. This suggest the data were not missing completely at random and may risk introducing bias, although they comprised a small overall proportion of cases and available variables suggest the excluded cases were similar to the analysed dataset (see appendix, missingness analysis table 2).

## DISCUSSION

This is the largest observational study published from a humanitarian organization describing the types of anaesthesia providers employed and the pattern of their work in a number of different settings. While not all humanitarian organizations (and MSF operational centres) operate in the same way as MSF-OCB, this study provides useful insights that may contribute towards their operational strategies.

Over 10 years of surgical activity by MSF-OCB, we found that anaesthesia provision was led by PAs during 66% of all cases in HG and AC settings (bearing in mind PA-led does not mean PAs administered the anaesthesia) with NA-led provision accounting for 19% and UA-led provision accounting for 15% of cases. While there was variation in the surgical caseload between provider types (PAs led more commonly in trauma-related surgery, NAs led more commonly in obstetric surgery), all providers led during surgery on both very sick (ASA grade 5) and very young patients (aged only a few days). In locations with UA-led anaesthesia, which was predominantly in the WHO Africa region, there was also a reduced presence of specialized surgical providers and expatriate involvement, despite the patient profile and surgical caseload being largely similar to that encountered in PA-led surgical projects in similar settings.

MSF tries to avoid employing unqualified anaesthesia providers, and they continue to evaluate means of mitigating this risk. However, a set of unique circumstances makes it unavoidable on occasion: 1) MSF, like many humanitarian organizations, operate predominantly in locations where there is a pre-existing anaesthesia workforce shortage,[15] and often in situations where this shortage may be exacerbated due to armed conflict or population displacement. 2) Expatriate staff are not always available, as MSF only deploy senior qualified anaesthetists as expatriate PAs, and it may not be possible for them to take time away from work at short notice. 3) Even if expatriate staff are available, in many contexts they have become deliberate targets. This has led to more cautious deployment of expatriate personnel into volatile settings.[16]

In this study we report briefly on intra-operative mortality. Rates are comparable across the different lead providers and similar to other observational data from LMICs[17–21] and some humanitarian organisations (including other MSF operational centres),[4,22,23] while higher than other humanitarian organisations.[24,25] However, such data must be interpreted cautiously as they should ideally be adjusted more fully for case-mix and severity. Further, most mortality related to surgery occurs in the days following surgery, and not in


For peer review only - http://bmjopen.bmj.com/site/about/guidelines.xhtml

theatre,[23,26,27] and these data are not available as part of the routine data we analysed. Therefore, while a more appropriate and widely recognized measure of surgical outcomes is perioperative mortality, which is advocated by both the Lancet Commission on Global Surgery and the World Health Organisation.[28,29] we were unable to report this. Further research into surgical outcomes in the humanitarian setting, which includes perioperative mortality and the incidence of post-operative complications and how they might differ between different anaesthesia providers, would be useful to assist organizations in providing safe and efficient anaesthesia in resource limited situations.

**Limitations**

Data quality is a known issue when using surveillance data, and considering the at times unpredictable nature of working in humanitarian settings, there is a risk of further decline in quality. However, due to the rigor in data monitoring centrally by MSF-OCB on a regular basis as described in the methods section, much has been done to minimize both missing data and improve the quality of the collected dataset. Our approach does have a particular risk of misclassification related to expatriate physician presence. Cases or projects could have been identified as 'PA-led', but the PA may not actually have been in the operating room for a variety of reasons including overseeing multiple theatres, or curfew and security concerns. Such misclassification could underrepresent the proportion of work where non-physicians were effectively sole providers. Our results therefore likely present a conservative estimate of the care provided by NA and UA. Finally, it is important to note that some projects had started before the start of routine data collection in 2008. Therefore, projects with expatriate PAs providing on-the-job training for UAs in the period before 2008 will not be reflected in our dataset.

## CONCLUSION

The majority of MSF anaesthesia care is led by PAs. However in conflict and healthcare gap settings, NAs and UAs are also major providers, and all providers encounter both the extremely young and the extremely sick in these challenging contexts. The humanitarian sector has considerable experience with task sharing and shifting, and lessons may be learned for more stable settings that could contribute to system strengthening. Despite their limitations routine data are key to monitoring the effectiveness of health systems, including humanitarian care, at scale and the MSF-OCB dataset is an important resource demonstrating that valuable data can be collected even in difficult circumstances. There is a need for wider engagement by the humanitarian community to continue to improve the collection and use of valid surgical outcome data. This would promote learning on how to optimize the surgical and anaesthetic workforce and help to ensure safe surgical and anaesthetic care, learning that could be valuable in many LMIC routine health settings.

## COMPETING INTERESTS

SK received funding from NIHR through their academic clinical fellowship scheme. ME received funding from a Wellcome Trust Senior Fellowship (#207522) as part of an unrelated research grant. All authors except from SK, ME, and HE are employed by MSF-OCB.


For peer review only - http://bmjopen.bmj.com/site/about/guidelines.xhtml

## AUTHOR CONTRIBUTIONS

SK helped conceive the study design, analyse the data, interpret the data, write the initial draft of the manuscript, and edit the manuscript. MT helped conceive the study design, collect the data, interpret the data, and edit the manuscript. ENB, LH, AM, RH, CStV, KS, AM, and SG helped collect the data and edit the manuscript. JK helped collect the data, interpret the data and edit the manuscript. HE and ME helped conceive the study design, interpret the data, and edit the manuscript.

## LEGEND OF FIGURES

- Figure 1: Flow diagram showing inclusion/exclusion of data and points of data linkage
- Figure 2: World maps showing number of (A) Surgical projects and (B) Surgical cases in each WHO region in settings of (1) conflict, (2) health care gaps, and (3) natural disasters
- Figure 3: Timelines showing duration and point in time all included surgical projects were active

## REFERENCES

1 Meara JG, Leather AJM, Hagander L, *et al.* Global Surgery 2030: Evidence and solutions for achieving health, welfare, and economic development. *Lancet* 2015;**386**:569–624. doi:10.1016/S0140-6736(15)60160-X
2 Craven RM. Managing anaesthetic provision for global disasters. *Br J Anaesth* 2017;**119**:i126–34. doi:10.1093/bja/aex353
3 White MC, Randall K, Avara E, *et al.* Clinical Outcome, Social Impact and Patient Expectation: a Purposive Sampling Pilot Evaluation of Patients in Benin Seven Years After Surgery. *World J Surg* 2018;**42**:1254–61. doi:10.1007/s00268-017-4296-9
4 Trudeau MON, Baron E, Hérard P, *et al.* Surgical care of pediatric patients in the humanitarian setting: The médecins sans frontières experience, 2012-2013. *JAMA Surg* 2015;**150**:1080–5. doi:10.1001/jamasurg.2015.1928
5 Ariyo P, Trelles M, Helmand R, *et al.* Providing Anesthesia Care in Resource-limited Settings: A 6-year Analysis of Anesthesia Services Provided at Médecins Sans Frontières Facilities. *Anesthesiology* 2016;**124**:561–9.
6 Chu KM, Ford N, Trelles M. Operative Mortality in Resource-Limited Settings: The Experience of Medecins Sans Frontières in 13 Countries. *Arch Surg* 2010;**145**:721–5.
7 Zitzman E, Berkley H, Jindal RM. Accountability in global surgery missions. *BMJ Glob Heal* 2018;**3**:e001025. doi:10.1136/bmjgh-2018-001025
8 Shrime MG, Sleemi A, Ravilla TD. Charitable platforms in global surgery:A systematic review of their effectiveness, cost-effectiveness, sustainability, and role training. *World J Surg* 2015;**39**:10–20. doi:10.1007/s00268-014-2516-0
9 Hendriks TCC, Botman M, Rahmee CNS, *et al.* Impact of short-term reconstructive surgical missions: A systematic review. *BMJ Glob Heal* 2019;**4**. doi:10.1136/bmjgh-2018-001176
10 Chu KM, Ford NP, Trelles M. Providing surgical care in Somalia: A model of task shifting. *Confl Health* 2011;**5**:12. doi:10.1186/1752-1505-5-12
11 Plecker E De, Zachariah R, Kumar AM V., *et al.* Emergency obstetric care in a rural district of Burundi: What are the surgical needs? *PLoS One* 2017;**12**:e0170882. doi:10.1371/journal.pone.0170882
12 World Health Organisation. Regional offices. https://www.who.int/about/who-we-are/regional-offices (accessed 4 Jul 2019).
13 Benchimol EI, Smeeth L, Guttmann A, *et al.* The REporting of studies Conducted using Observational Routinely-collected health Data (RECORD) statement. *PLoS Med*


For peer review only - http://bmjopen.bmj.com/site/about/guidelines.xhtml

2015;**12**:e1001885. doi:10.1371/journal.pmed.1001885
14 Foreign Medical Team Working Group. Classification and Minimum Standards for Foreign Medical Teams in Sudden Onset. Geneva: 2013.
15 Kempthorne P, Morriss WW, Mellin-Olsen J, *et al.* The WFSA Global Anesthesia Workforce Survey. *Anesth Analg* 2017;**125**:981–90. doi:10.1213/ANE.0000000000002258
16 ICRC. Health Care in Danger: A sixteen-country study. Geneva: 2011.
17 Kwari YD, Bello MR, Eni UE. Pattern of perioperative cardiac arrests at University of Maiduguri Teaching Hospital. *Niger J Med J Natl Assoc Resid Dr Niger* 2010;**19**:173–6.
18 Zoumenou E, Gbenou S, Assouto P, *et al.* Pediatric anesthesia in developing countries: experience in the two main university hospitals of Benin in West Africa. *Paediatr Anaesth* 2010;**20**:741–7.
19 Walker IA, Obua AD, Mouton F, *et al.* Paediatric surgery and anaesthesia in south-western Uganda: a cross-sectional survey. *Bull World Health Organ* 2010;**88**:897–906.
20 Rukewe A, Fatiregun A, Adebayo K. Anaesthesia for caesarean deliveries and maternal complications in a Nigerian teaching hospital. *African J Med Med Sci* 2014;**43**:5–10.
21 Rukewe A, Fatiregun A, Osunlaja TO. Cardiac arrest during anesthesia at a university Hospital in Nigeria. *Niger J Clin Pract* 2014;**17**:28–31.
22 Wichlas F, Tsitsilonis S, Guanti MD, *et al.* Orthopedic trauma surgeon in Sierra Leone: how to keep one's head over water. *Arch Orthop Trauma Surg* 2019;**0**:0. doi:10.1007/s00402-019-03171-1
23 Davies JF, Lenglet A, Van Wijhe M, *et al.* Perioperative mortality: Analysis of 3 years of operative data across 7 general surgical projects of Medecins Sans Frontieres in Democratic Republic of Congo, Central African Republic, and South Sudan. *Surgery* 2016;**159**:1269–78. doi:10.1016/j.surg.2015.12.022
24 Fisher QA, Nichols D, Stewart FC, *et al.* Assessing Pediatric Anesthesia Practices for Volunteer Medical Services Abroad. *Anesthesiol J Am Soc Anesthesiol* 2001;**95**:1315–22.
25 White MC, Barki BJ, Lerma SA, *et al.* A prospective observational study of anesthesia- related adverse events and postoperative complications occurring during a surgical mission in Madagascar. *Anesth Analg* 2018;**127**:506–12.
26 Biccard BM, Madiba TE, Kluyts H-L, *et al.* Perioperative patient outcomes in the African Surgical Outcomes Study: a 7-day prospective observational cohort study. *Lancet* 2018;**391**:1589–98. doi:10.1016/S0140-6736(18)30001-1
27 Ng-Kamstra JS, Arya S, Greenberg SLM, *et al.* Perioperative mortality rates in low-income and middle-income countries: a systematic review and meta-analysis. *BMJ Glob Heal* 2018;**3**:e000810. doi:10.1136/bmjgh-2018-000810
28 Holmer H, Bekele A, Hagander L, *et al.* Evaluating the collection, comparability and findings of six global surgery indicators. *Br J Surg* 2019;:e138–50. doi:10.1002/bjs.11061
29 World Health Organisation. 2018 Global reference list of 100 core health indicators (plus health-related SDGs). Geneva: 2018.

For peer review only

**Figure 1: Flow diagram showing inclusion/exclusion of data and points of data linkage**

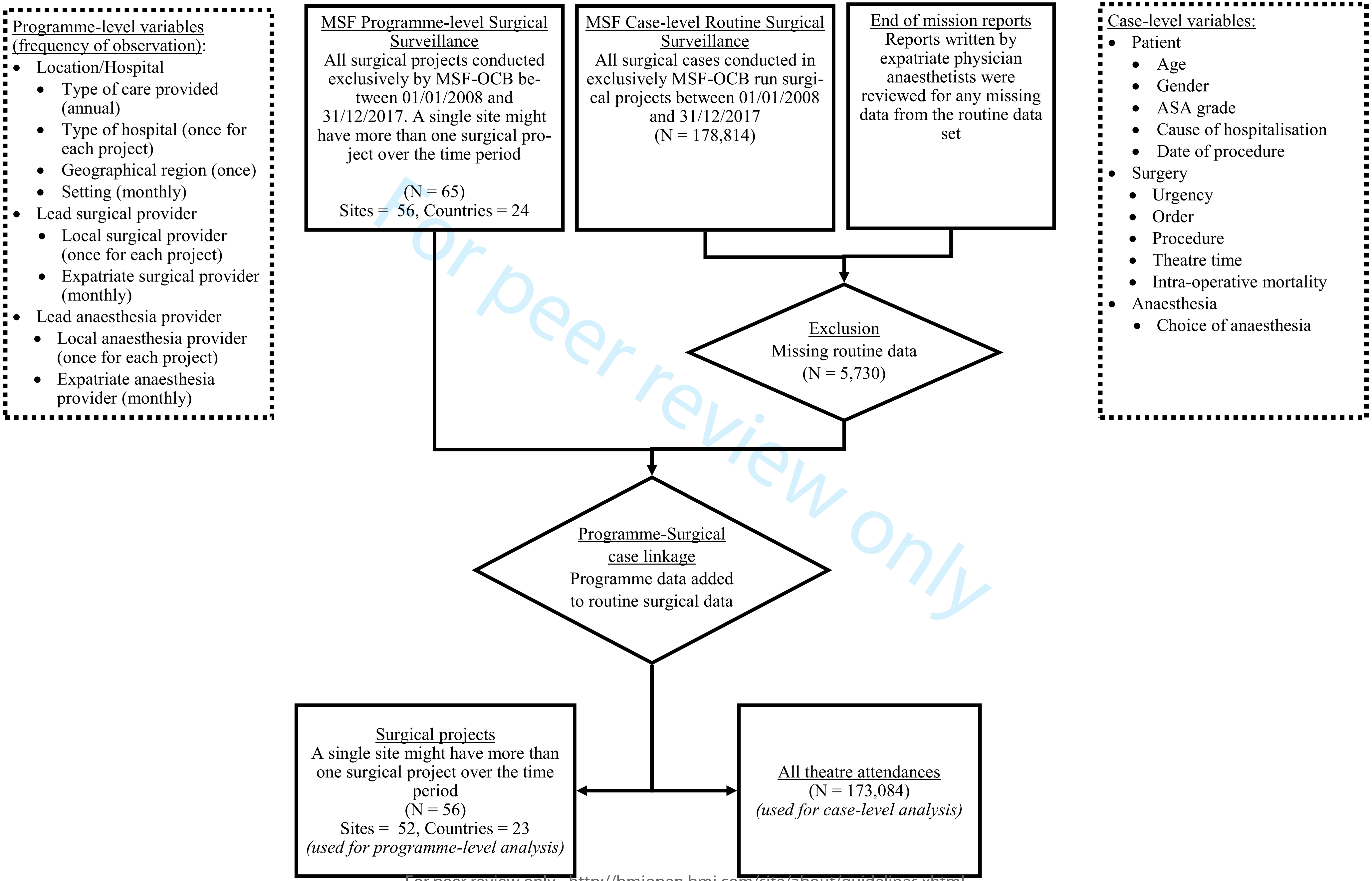


For peer review only

Figure 2: World maps showing number of (A) Surgical projects and (B) Surgical cases in each WHO region in settings of (1) conflict, (2) health care gaps, and (3) natural disasters

**A**

| WHO regions | Conflict setting | Health care gap setting | Natural disaster setting |
|---|---|---|---|
| Africa region | 21 | 21 | 0 |
| Americas region | 0 | 3 | 3 |
| Eastern Mediterranean region | 7 | 8 | 0 |
| South−East Asia region | 0 | 2 | 1 |
| Western Pacific region | 0 | 0 | 1 |

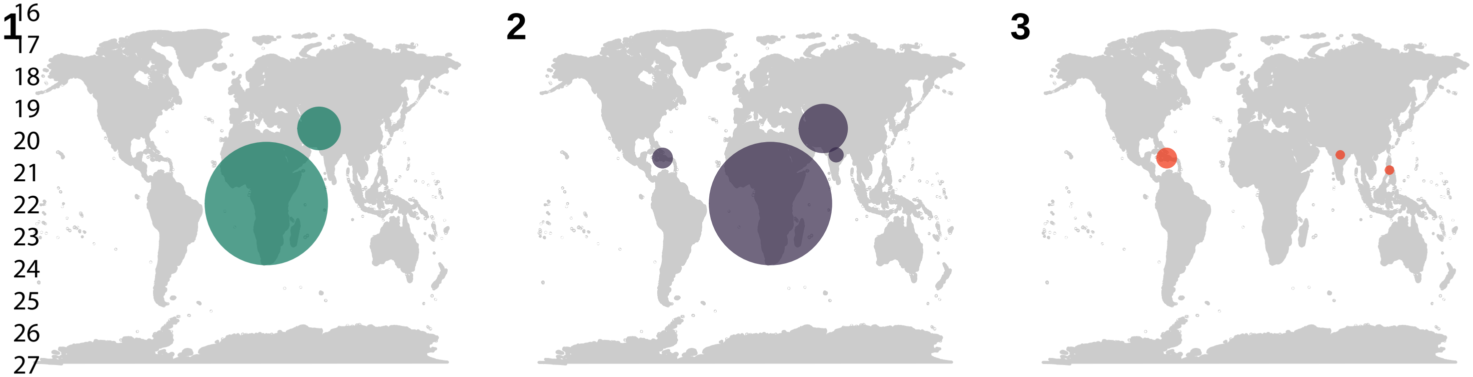


**B**

| WHO regions | Conflict setting | Health care gap setting | Natural disaster setting |
|---|---|---|---|
| Africa region | 16087 | 65551 | 0 |
| Americas region | 0 | 33319 | 2730 |
| Eastern Mediterranean region | 26138 | 28066 | 0 |
| South−East Asia region | 0 | 815 | 89 |
| Western Pacific region | 0 | 0 | 289 |

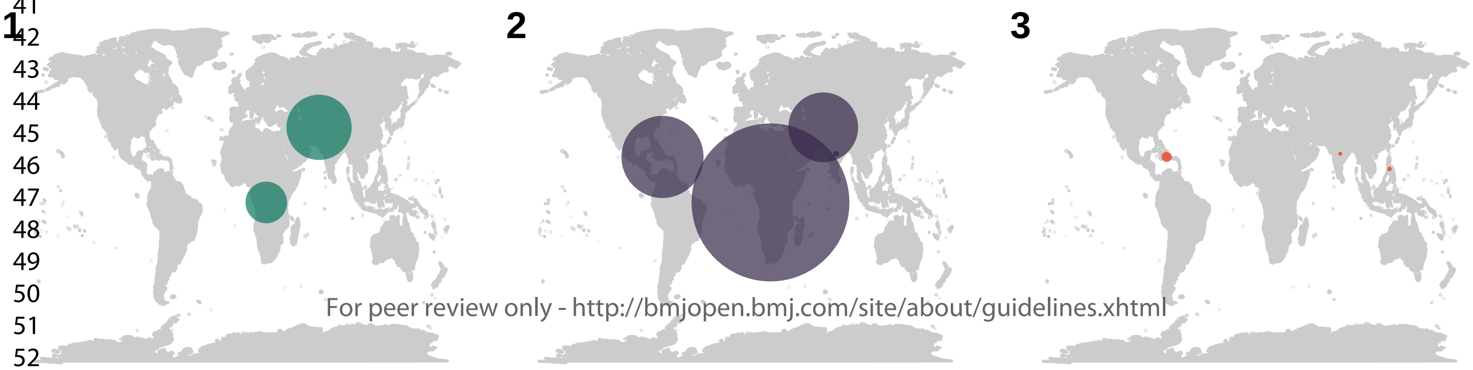


For peer review only - http://bmjopen.bmj.com/site/about/guidelines.xhtml

Figure 3: Timelines showing duration and point in time all included surgical projects were active

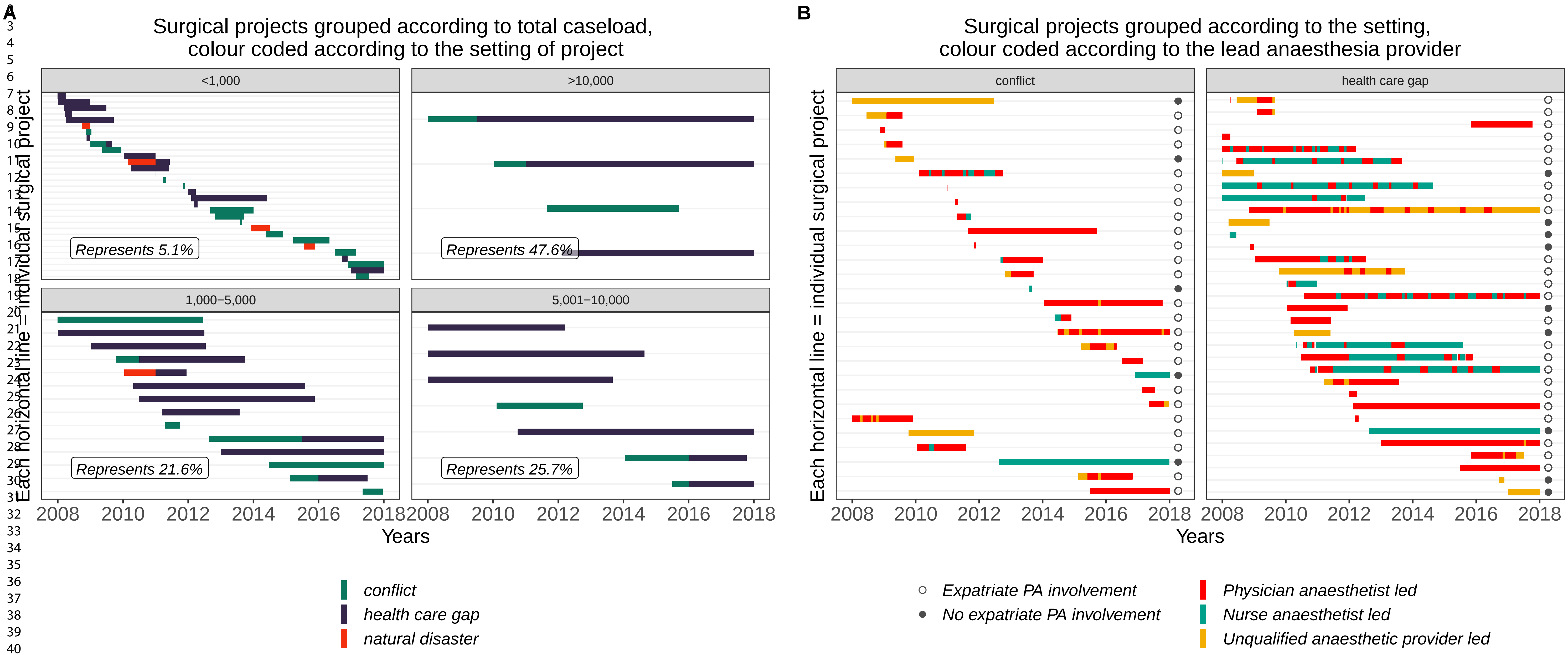




*Excludes periods where projects are run in collaboration with other organisations or local government. Additionally, only data from 2008 till 2017 are included. Therefore, periods with expatriate physician anaesthetist (PA) involvement before then are not reflected here.

# Appendix

**Table of Contents**

1. Table 1: Variables used in the study
2. Table 2: surgical projects in health care gap settings in the WHO SEA region (2 in total)
3. Table 3: Surgical projects in natural disaster settings (5 in total)
4. Table 4: Surgical groupings as used in main table 2 (case-level data)
5. Missingness analysis

   Table 1: Number of missing values within each variable

   Figure 1: matrixplot of all variables (along the x-axis), sorted according to year (the darker the color the higher the number; red indicates missing value)

   Figure 2: histogram showing all surgical projects (along x-axis), sorted according to setting, showing proportion of missing cases with any missing data (red indicates data with at least one missing value)

   Table 2: overview of available data

For peer review only

# 1. Table 1: Variables used in the study

<table>
<tr><th>Key characteristic group</th><th>Name of variable</th><th>Type of data</th><th>Description of data</th><th>Source</th></tr>
<tr><td rowspan="5">Patient</td><td>Age</td><td>Continuous</td><td>For patients below the age of 2 (typed in original data collection sheet as days and months), the age has been converted for analysis to a fraction of a year.</td><td>Case-level routine data</td></tr>
<tr><td>Gender</td><td>Categorical (binary)</td><td>Either male or female</td><td>Case-level routine data</td></tr>
<tr><td>Date of procedure</td><td>Continuous</td><td>Date operation took place</td><td>Case-level routine data</td></tr>
<tr><td>ASA grade</td><td>Ordinal</td><td>American Society of Anaesthesiologists physical status classification system (ASA). Discrete numeric scale between 1 and 5 (1 = normal healthy patient, 5 = moribund patient expected not to survive without surgery) of the patient's physical health prior to surgery.</td><td>Case-level routine data</td></tr>
<tr><td>Cause of hospitalisation</td><td>Categorical (nominal)</td><td>3 letter code used as defined by MSF-OCB operational departmental guideline. A total of 24 codes available, and grouped into 4 distinct categories:<br>- Accidental trauma<br>- Violent trauma<br>- Obstetric<br>- Other (including but not limited to tropical disease, tumours, obstruction)</td><td>Case-level routine data</td></tr>
<tr><td rowspan="4">Surgery</td><td>Urgency</td><td>Categorical (ordinal)</td><td>3 values available, relating to how soon the surgical procedure has to occur:<br>- "Urgent" = requiring immediate surgery<br>- "Delayed" = requiring surgery during current hospital admission<br>- "Planned" = elective surgery</td><td>Case-level routine data</td></tr>
<tr><td>Order</td><td>Categorical (ordinal)</td><td>3 codes available, relating to whether patient has had surgery before during admission:<br>- "First" = first time entering theatre<br>- "Unplanned" = unplanned return to theatre<br>- "Re-intervention" = planned return to theatre</td><td>Case-level routine data</td></tr>
<tr><td>Procedure – Main group</td><td>Categorical (nominal)</td><td>2 letter code used as defined by MSF-OCB operational department guidelines. A total of 36 procedures codes available, and grouped into the main categories of surgery. See appendix table 4 for full breakdown.</td><td>Case-level routine data</td></tr>
<tr><td>Lead surgical provider</td><td>Categorical (nominal)</td><td>A merged variable, based on the presence of local and expatriate providers as outlined below. MD signifies a physician without surgical qualifications but with surgical skills.
<table>
<tr><th></th><th>Local provider</th><th>Expatriate provider</th></tr>
<tr><td rowspan="4">General & specialist surgeon</td><td>None</td><td>General & Specialist</td></tr>
<tr><td>MD</td><td>General & Specialist</td></tr>
<tr><td>General</td><td>Specialist</td></tr>
<tr><td>General & Specialist</td><td>Any</td></tr>
<tr><td rowspan="3">Specialist only</td><td>None</td><td>Specialist</td></tr>
<tr><td>MD</td><td>Specialist</td></tr>
<tr><td>Specialist</td><td>Specialist or none</td></tr>
</table></td><td>Programme-level routine data (MT)</td></tr>
</table>

For peer review only - http://bmjopen.bmj.com/site/about/guidelines.xhtml

<table>
<tr><td></td><td></td><td></td><td><table><tr><td>General only</td><td>None</td><td>General</td></tr><tr><td></td><td>MD</td><td>General</td></tr><tr><td></td><td>General</td><td>General or none</td></tr><tr><td>MD</td><td>MD</td><td>None</td></tr></table></td><td></td></tr>
<tr><td></td><td>Theatre time (min)</td><td>Continuous</td><td>Time in minutes the patient was occupying theatre. This included anaesthetic and surgical time, as well as any recovery of patient, which occurred in theatre.</td><td>Case-level routine data</td></tr>
<tr><td></td><td>Intra-operative mortality</td><td>Categorical (binary)</td><td>Whether the patient was dead or alive by the time they left recovery. For the purpose of the study, this is considered as intraoperative mortality.</td><td>Case-level routine data</td></tr>
<tr><td rowspan="2">Anaesthesia</td><td>Choice of anaesthesia</td><td>Categorical (nominal)</td><td>List of 7 codes of anaesthesia that can be provided. Only a single code can be used for a surgical procedure:<br>- Local anaesthesia<br>- Regional anaesthesia<br>- Spinal anaesthesia<br>- General anaesthesia without intubation or muscle relaxant<br>- General anaesthesia with intubation and/or muscle relaxant<br>- Combined anaesthesia (if more than one code need to be used, e.g. spinal anaesthesia + general anaesthesia)<br>- Other anaesthesia, e.g. sedation</td><td>Case-level routine data</td></tr>
<tr><td>Lead anaesthesia provider</td><td>Categorical (nominal)</td><td>A merged variable, based on the presence of local and expatriate providers as outlined below.<table><tr><td></td><td>Local provider</td><td>Expatriate provider</td></tr><tr><td rowspan="4">Physician anaesthetist (PA) led</td><td>PA</td><td>none</td></tr><tr><td>NA</td><td>PA</td></tr><tr><td>UA</td><td>PA</td></tr><tr><td>None</td><td>PA</td></tr><tr><td rowspan="3">Nurse anaesthetist (NA) led</td><td>NA</td><td>none</td></tr><tr><td>UA</td><td>NA</td></tr><tr><td>None</td><td>NA</td></tr><tr><td>Unqualified anaesthetic provider (UA) led</td><td>UA</td><td>None</td></tr></table></td><td>Programme-level routine data (MT)</td></tr>
<tr><td>Setting</td><td>Type of care provided by hospital</td><td>Categorical (nominal)</td><td>Overall type of provision provided during surgical project, based on MSF-OCB brief. 9 separate categories grouped into 5:<br>- Emergency only<br>- Capacity to perform both emergency and elective surgery<br>- Maternity care only<br>- Trauma care only<br>- Other specific care provision (wound care, trauma and surgical care, obstetric fistula care, and surgical care of typhoid related complications)</td><td>Programme-level routine data (MT)</td></tr>
</table>

| | | | | |
|---|---|---|---|---|
| | WHO region | Categorical (nominal) | The location of each mission was labelled according to the region codes used by the World Health Organisation:<br>- AFR<br>- EMR<br>- SEAR<br>- AMR<br>- WPR | WHO |
| | Setting | Categorical (binary) | 3 variables:<br>- Conflict<br>- Natural disaster<br>- Health care gaps | Programme-level routine data (MT) |
| | Hospital level | Ordinal (Categorical) | 4 distinct categories as per MSF-OCB surgical policy guidelines:<br>- Sole remit hospital = hospital that provides care for a specific purpose (i.e. not necessarily a quaternary referral hospital, but a surgical setup for a specific indication). Examples include: Trauma centre, Maternity centre, Fistula repair camp.<br>- Referral hospital = provincial hospital, considered tertiary referral hospital.<br>- District hospital = can manage most, but will refer complex cases on to referral hospitals.<br>- Health centres = small rural health centres with capacity to perform basic surgical operations | Programme-level routine data (MT) |
| | Site ID | Character | Anonymous unique code for each site | produced in R 3.6 |

For peer review only

## 2. Table 2: surgical projects in health care gap settings in the WHO SEA region (2 in total)

| | |
|---|---|
| **Type of anaesthesia provider** | Physician anaesthetist only |
| **Type of hospital** | - District hospital<br>- Health centre |
| **Type of care provided by surgical project (No. of projects)** | - Capacity to perform both emergency and elective surgery<br>- Filiariasis-related care |
| **Total duration, months** | 19 |
| **Type of surgical provider for entire duration** | - General & specialty surgeons<br>- General surgeon only |
| **Total number of all cases, No.** | 815 |
| **Main cause for hospitalisation, No. (%)** | - Other, 482 (59) |
| **Main category of surgery, No. (%)** | - Minor Surgery, 507 (62) |
| **Intra-operative mortality, No. (%)** | 2 (0.2) |

For peer review only

# 3. Table 3: Surgical projects in natural disaster settings (5 in total)

| | |
|---|---|
| **Type of anaesthesia provider** | Physician anaesthetist only |
| **Type of hospital** | - District<br>- Sole remit hospital |
| **Type of care provided by surgical project (No. of projects)** | - Capacity to perform both emergency and elective surgery (2)<br>- Trauma care only (1)<br>- Emergency only (2) |
| **Total duration, months** | 40 |
| **Presence of surgical provider, months** | |
| - **General and specialty surgeons** | 23 |
| - **General surgeon only** | 16 |
| - **Specialty surgeon only** | 1 |
| **Total number of all cases, No. (%)** | 3108 |
| **Main cause for hospitalisation, No. (%)** | - Other, 1144 (37) |
| **Main category of surgery, No. (%)** | - Minor surgery, 1608 (52) |
| **Intra-operative mortality, No. (%)** | 9 (0.3) |

For peer review only

## 4. Table 4: Surgical groupings as used in main table 2 (case-level data)

| Surgical grouping | Examples of types of surgery included |
|---|---|
| **Minor surgery** | • Simple wound treatment<br>• Insertion/removal of drain<br>• Burns dressing change<br>• Wound debridement<br>• Removal of foreign body<br>• Amputation of digits or toes<br><br>Incl. procedure codes with median operative time < 45min within the dataset:<br><br>• Curettage post delivery (GP)<br>• Reduction of fractures (OR)<br>• Removal of osteosynthesis (OX)<br>• Ophthalmic surgery (SO) |
| **Caesarean section** | • Caesarean section only |
| **Visceral surgery** | • Exploratory laparotomy<br>• Hernia repair<br>• Resection/repair solid organs (e.g. spleen/liver) or gut |
| **Orthopaedic surgery** | • External or internal fixation of fracture<br>• Surgery to any joint<br>• Limb amputation (excluding digits or toes)<br>• Curettage for osteomyelitis |
| **Obstetric & gynaecological surgery (excl. Caesarean section)** | • Management of ectopic pregnancy<br>• Obstetric fistula repair<br>• Hysterectomy |
| **Specialties** | • Urology<br>• Vascular surgery<br>• Plastic and reconstructive surgery<br>• Ear, nose and throat surgery<br>• Neurosurgery<br>• Thoracic surgery<br>• Maxillofacial surgery<br>• Other specialized surgery |

Modified from the original surgical groupings outlined in the "MSF-OCB Operating Department Data Collection Guidelines (2015)".

For peer review only

## 5. Missingness analysis

**Table 1: Number of missing values within each variable**

| Variable | Number missing |
|---|---|
| ASA | 3232 |
| Intra-operative mortality | 2154 |
| Time in theatre | 1922 |
| Age | 47 |
| Main procedure | 5 |
| Choice of anaesthesia | 2 |
| Gender | 0 |
| Date of procedure | 0 |
| Cause of hospitalisation | 0 |
| Urgency | 0 |
| Order | 0 |
| Surgical provider | 0 |
| Anaesthesia provider | 0 |
| Who region | 0 |
| Setting | 0 |
| Type of hospital | 0 |
| Type of care provided | 0 |
| Site ID | 0 |

For peer review only

**Figure 1: matrixplot of all variables (along the x-axis), sorted according to year (the darker the colour the higher the number; red indicates missing value)**

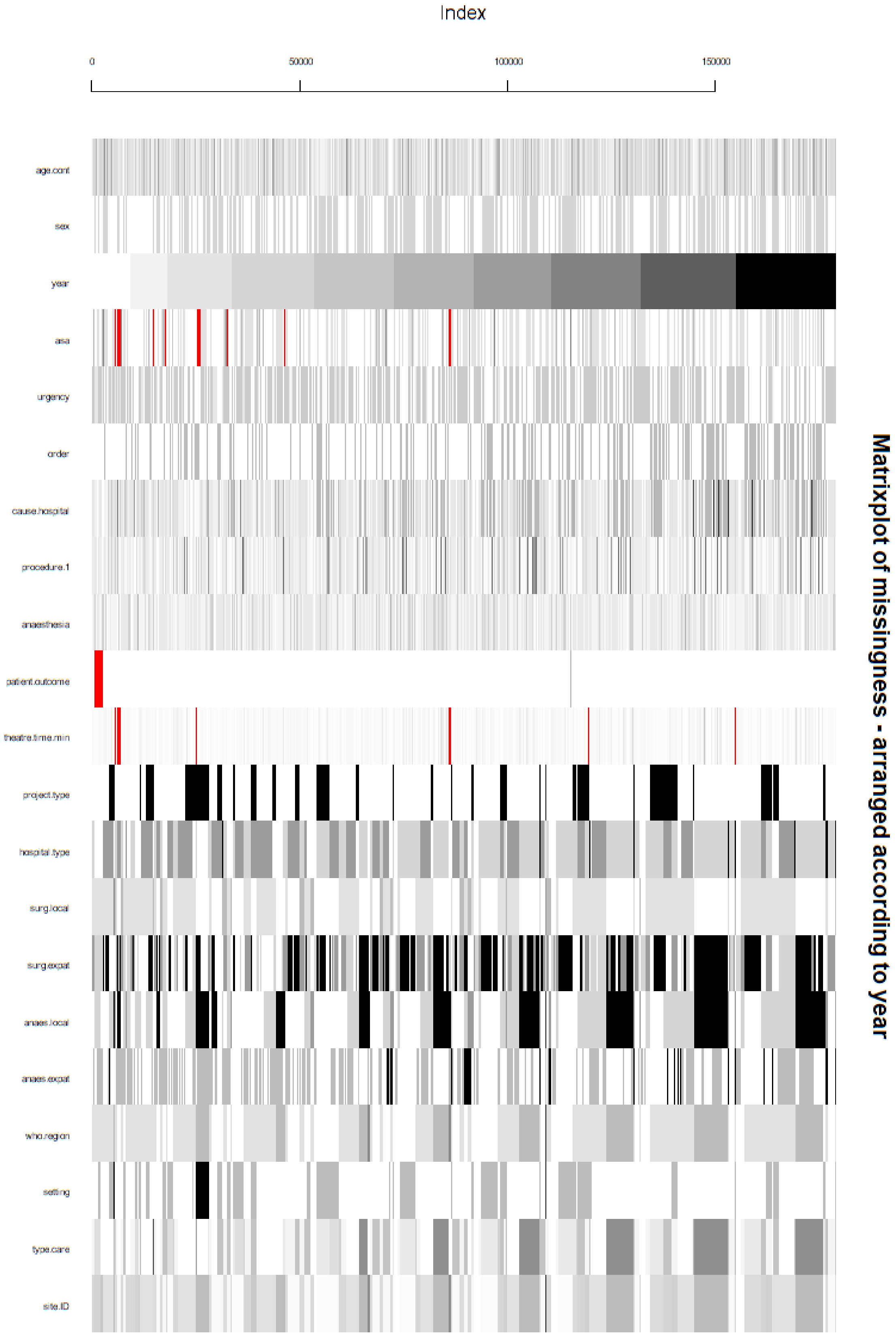

**Figure 2: histogram showing all surgical projects (along x-axis), sorted according to setting, showing proportion of missing cases with any missing data (red indicates data with at least one missing value)**

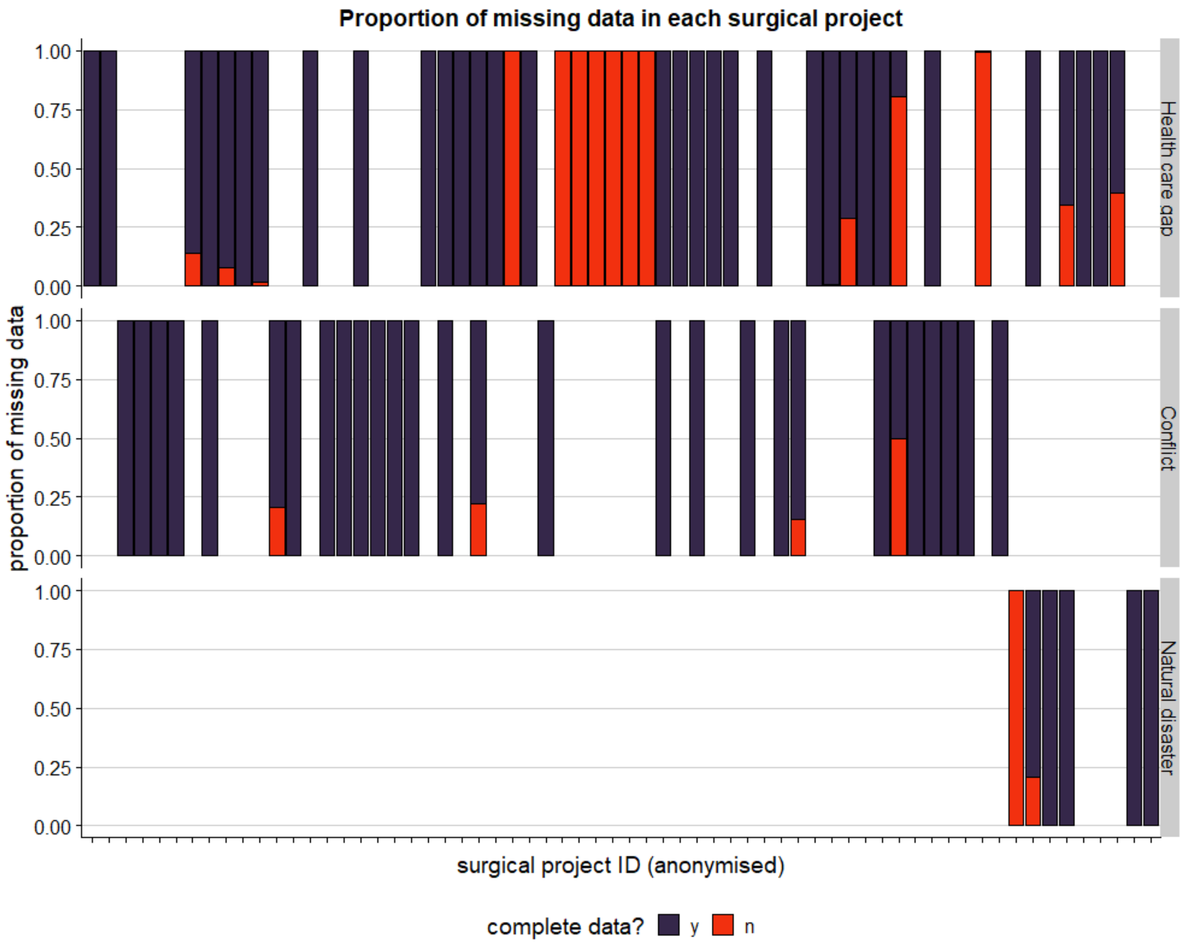


For peer review only

# Table 2: overview of available data

| Variable | Value |
|---|---|
| **Median age, years (IQR)** | 26 (18-36) |
| **Female, no. (%)** | 2963 (52) |
| **Median ASA (IQR)** | 1 (1-2) |
| **Emergent surgery, no. (%)** | 2277 (40) |
| **Cause for hospitalisation, no. (%)** | |
| - **Obstetric** | 1188 |
| - **Any trauma** | 2074 |
| - **Other** | 2468 |
| **Main surgical procedure** | |
| - **Minor surgery** | 2854 |
| - **Caesarean section** | 746 |
| - **Visceral surgery** | 1408 |
| - **Obstetric and gynaecology** | 503 |
| - **Orthopaedics** | 177 |
| - **Other specialty surgery** | 37 |
| **Intraoperative mortality** | |
| - **Alive** | 3563 |
| - **Died** | 13 |
| **Median theatre time, minutes (IQR)** | 45 (35-60) |
| **Setting** | |
| - **Health care gap** | 3359 |
| - **Conflict** | 1443 |
| - **Natural disaster** | 928 |

For peer review only

**The use of different anaesthesia providers in humanitarian settings: Descriptive study of 173,084 episodes of surgical care provided by Médecins Sans Frontières over 10 years**

The RECORD statement – checklist of items, extended from the STROBE statement, that should be reported in observational studies using routinely collected health data.

| | Item No. | STROBE items | Location in manuscript where items are reported | RECORD items | Location in manuscript where items are reported |
|---|---|---|---|---|---|
| **Title and abstract** | | | | | |
| | 1 | (a) Indicate the study's design with a commonly used term in the title or the abstract | Title & Abstract | RECORD 1.1: The type of data used should be specified in the title or abstract. When possible, the name of the databases used should be included. | Abstract |
| | | (b) Provide in the abstract an informative and balanced summary of what was done and what was found | Abstract | RECORD 1.2: If applicable, the geographic region and timeframe within which the study took place should be reported in the title or abstract. | Abstract |
| | | | | RECORD 1.3: If linkage between databases was conducted for the study, this should be clearly stated in the title or abstract. | Abstract |
| **Introduction** | | | | | |
| Background rationale | 2 | Explain the scientific background and rationale for the investigation being reported | Introduction | | |
| Objectives | 3 | State specific objectives, including any prespecified hypotheses | Introduction | | |
| **Methods** | | | | | |
| Study Design | 4 | Present key elements of study design early in the paper | Methods | | |
| Setting | 5 | Describe the setting, locations, and relevant dates, including | Methods | | |

For peer review only

| | | | | | |
|---|---|---|---|---|---|
| | | periods of recruitment, exposure, follow-up, and data collection | | | |
| Participants | 6 | *(a) Cohort study* - Give the eligibility criteria, and the sources and methods of selection of participants. Describe methods of follow-up<br>*Case-control study* - Give the eligibility criteria, and the sources and methods of case ascertainment and control selection. Give the rationale for the choice of cases and controls<br>*Cross-sectional study* - Give the eligibility criteria, and the sources and methods of selection of participants<br><br>*(b) Cohort study* - For matched studies, give matching criteria and number of exposed and unexposed<br>*Case-control study* - For matched studies, give matching criteria and the number of controls per case | N/A<br><br>N/A<br><br>N/A<br><br>N/A<br><br>N/A | RECORD 6.1: The methods of study population selection (such as codes or algorithms used to identify subjects) should be listed in detail. If this is not possible, an explanation should be provided.<br><br>RECORD 6.2: Any validation studies of the codes or algorithms used to select the population should be referenced. If validation was conducted for this study and not published elsewhere, detailed methods and results should be provided.<br><br>RECORD 6.3: If the study involved linkage of databases, consider use of a flow diagram or other graphical display to demonstrate the data linkage process, including the number of individuals with linked data at each stage. | Methods<br><br>N/A<br><br>Inclusion flow diagram |
| Variables | 7 | Clearly define all outcomes, exposures, predictors, potential confounders, and effect modifiers. Give diagnostic criteria, if applicable. | Appendix | RECORD 7.1: A complete list of codes and algorithms used to classify exposures, outcomes, confounders, and effect modifiers should be provided. If these cannot be reported, an explanation should be provided. | Appendix |
| Data sources/ measurement | 8 | For each variable of interest, give sources of data and details of methods of assessment (measurement). | Appendix | | |

For peer review only

| | | | | | |
|---|---|---|---|---|---|
| | | Describe comparability of assessment methods if there is more than one group | N/A | | |
| Bias | 9 | Describe any efforts to address potential sources of bias | Methods - bias | | |
| Study size | 10 | Explain how the study size was arrived at | Descriptive study – not performed | | |
| Quantitative variables | 11 | Explain how quantitative variables were handled in the analyses. If applicable, describe which groupings were chosen, and why | Appendix | | |
| Statistical methods | 12 | (a) Describe all statistical methods, including those used to control for confounding<br>(b) Describe any methods used to examine subgroups and interactions<br>(c) Explain how missing data were addressed<br>(d) *Cohort study* - If applicable, explain how loss to follow-up was addressed<br>*Case-control study* - If applicable, explain how matching of cases and controls was addressed<br>*Cross-sectional study* - If applicable, describe analytical methods taking account of sampling strategy<br>(e) Describe any sensitivity analyses | Descriptive study – not performed<br>Methods – bias<br>Methods – appendix<br>n/a<br>n/a<br>n/a<br>results/appendix | | |
| Data access and cleaning methods | | .. | | RECORD 12.1: Authors should describe the extent to which the investigators had access to the database population used to create the study population. | Methods |

For peer review only

| | | | | | |
|---|---|---|---|---|---|
| | | | | RECORD 12.2: Authors should provide information on the data cleaning methods used in the study. | Methods |
| Linkage | | .. | | RECORD 12.3: State whether the study included person-level, institutional-level, or other data linkage across two or more databases. The methods of linkage and methods of linkage quality evaluation should be provided. | Methods & inclusion flow diagram |
| **Results** | | | | | |
| Participants | 13 | (a) Report the numbers of individuals at each stage of the study (*e.g.*, numbers potentially eligible, examined for eligibility, confirmed eligible, included in the study, completing follow-up, and analysed)<br>(b) Give reasons for non-participation at each stage.<br>(c) Consider use of a flow diagram | inclusion/exclusion diagram<br>N/A<br>inclusion/exclusion diagram | RECORD 13.1: Describe in detail the selection of the persons included in the study (*i.e.,* study population selection) including filtering based on data quality, data availability and linkage. The selection of included persons can be described in the text and/or by means of the study flow diagram. | Methods & inclusion/exclusio n diagram |
| Descriptive data | 14 | (a) Give characteristics of study participants (*e.g.*, demographic, clinical, social) and information on exposures and potential confounders<br>(b) Indicate the number of participants with missing data for each variable of interest<br>(c) *Cohort study* - summarise follow-up time (*e.g.*, average and total amount) | Results – table 1<br>Appendix<br>N/A | | |
| Outcome data | 15 | *Cohort study* - Report numbers of outcome events or summary measures over time | N/A<br>N/A | | |

For peer review only

| | | | | | |
|---|---|---|---|---|---|
| | | *Case-control study* - Report numbers in each exposure category, or summary measures of exposure<br>*Cross-sectional study* - Report numbers of outcome events or summary measures | Results | | |
| Main results | 16 | (a) Give unadjusted estimates and, if applicable, confounder-adjusted estimates and their precision (e.g., 95% confidence interval). Make clear which confounders were adjusted for and why they were included<br>(b) Report category boundaries when continuous variables were categorized<br>(c) If relevant, consider translating estimates of relative risk into absolute risk for a meaningful time period | Results<br><br>Results<br><br>N/A | | |
| Other analyses | 17 | Report other analyses done—e.g., analyses of subgroups and interactions, and sensitivity analyses | Results | | |
| **Discussion** | | | | | |
| Key results | 18 | Summarise key results with reference to study objectives | Discussion | | |
| Limitations | 19 | Discuss limitations of the study, taking into account sources of potential bias or imprecision. Discuss both direction and magnitude of any potential bias | Discussion | RECORD 19.1: Discuss the implications of using data that were not created or collected to answer the specific research question(s). Include discussion of misclassification bias, unmeasured confounding, missing data, and changing eligibility over time, as they pertain to the study being reported. | Discussion |

For peer review only

| | | | | | |
|---|---|---|---|---|---|
| Interpretation | 20 | Give a cautious overall interpretation of results considering objectives, limitations, multiplicity of analyses, results from similar studies, and other relevant evidence | Discussion | | |
| Generalisability | 21 | Discuss the generalisability (external validity) of the study results | Discussion | | |
| **Other Information** | | | | | |
| Funding | 22 | Give the source of funding and the role of the funders for the present study and, if applicable, for the original study on which the present article is based | Methods & competing interests | | |
| Accessibility of protocol, raw data, and programming code | | .. | | RECORD 22.1: Authors should provide information on how to access any supplemental information such as the study protocol, raw data, or programming code. | Methods |

*Reference: Benchimol EI, Smeeth L, Guttmann A, Harron K, Moher D, Petersen I, Sørensen HT, von Elm E, Langan SM, the RECORD Working Committee. The REporting of studies Conducted using Observational Routinely-collected health Data (RECORD) Statement. *PLoS Medicine* 2015; in press.

*Checklist is protected under Creative Commons Attribution (CC BY) license.

For peer review only

BMJ Open

# Anaesthesia care providers employed in humanitarian settings by Médecins Sans Frontières: A retrospective observational study of 173,084 surgical cases over 10 years

| | |
|---|---|
| Journal: | *BMJ Open* |
| Manuscript ID | bmjopen-2019-034891.R1 |
| Article Type: | Original research |
| Date Submitted by the Author: | 10-Dec-2019 |
| Complete List of Authors: | Kudsk-Iversen, Soren; Oxford University Hospitals NHS Foundation Trust, Nuffield Department of Anaesthetics<br>Trelles, Miguel; Medecins Sans Frontieres<br>Ngowa Bakebaanitsa, Elie; Medecins Sans Frontieres; Masisi referral hospital, Masisi – MSF Democratic Republic of the Congo mission<br>Hagabimana, Longin; Medecins Sans Frontieres; Arche trauma hospital, Bujumbura – MSF Burundi mission<br>Momen, Abdul; Medecins Sans Frontieres; Khost maternity, Khost – MSF Afghanistan mission<br>Helmand, Rahmatullah; Medecins Sans Frontieres; Ahmad Shah Baba hospital, Kabul – MSF Afghanistan mission<br>Saint Victor, Carline; Medecins Sans Frontieres; Tabarre trauma hospital, Port-au-Prince – MSF Haiti mission<br>Shah, Khalid; Medecins Sans Frontieres; Timurgara district headquarter hospital – MSF Pakistan mission<br>Masu, Adolphe; Medecins Sans Frontieres; Castors maternity, Bangui – MSF Central African Republic mission<br>Kendell, Judith; Medecins Sans Frontieres<br>Edgcombe, Hilary; Oxford University Hospitals NHS Foundation Trust, Nuffield Department of Anaesthetics<br>English, Mike; KEMRI - Wellcome Trust Research Programme, Health Services Unit; University of Oxford Centre for Tropical Medicine and Global Health |
| <b>Primary Subject Heading</b>: | Anaesthesia |
| Secondary Subject Heading: | Global health |
| Keywords: | ANAESTHETICS, Human resource management < HEALTH SERVICES ADMINISTRATION & MANAGEMENT, Organisation of health services < HEALTH SERVICES ADMINISTRATION & MANAGEMENT |
| | |

SCHOLARONE™
Manuscripts

*I, the Submitting Author has the right to grant and does grant on behalf of all authors of the Work (as defined in the below author licence), an exclusive licence and/or a non-exclusive licence for contributions from authors who are: i) UK Crown employees; ii) where BMJ has agreed a CC-BY licence shall apply, and/or iii) in accordance with the terms applicable for US Federal Government officers or employees acting as part of their official duties; on a worldwide, perpetual, irrevocable, royalty-free basis to BMJ Publishing Group Ltd ("BMJ") its licensees and where the relevant Journal is co-owned by BMJ to the co-owners of the Journal, to publish the Work in this journal and any other BMJ products and to exploit all rights, as set out in our licence.*

*The Submitting Author accepts and understands that any supply made under these terms is made by BMJ to the Submitting Author unless you are acting as an employee on behalf of your employer or a postgraduate student of an affiliated institution which is paying any applicable article publishing charge ("APC") for Open Access articles. Where the Submitting Author wishes to make the Work available on an Open Access basis (and intends to pay the relevant APC), the terms of reuse of such Open Access shall be governed by a Creative Commons licence – details of these licences and which Creative Commons licence will apply to this Work are set out in our licence referred to above.*

*Other than as permitted in any relevant BMJ Author's Self Archiving Policies, I confirm this Work has not been accepted for publication elsewhere, is not being considered for publication elsewhere and does not duplicate material already published. I confirm all authors consent to publication of this Work and authorise the granting of this licence.*

For peer review only

# Anaesthesia care providers employed in humanitarian settings by Médecins Sans Frontières: A retrospective observational study of 173,084 surgical cases over 10 years

Søren Kudsk-Iversen, MBChB DTM&H FRCA
Address:
Stoke Mandeville Hospital, Mandeville Rd, Aylesbury HP21 8AL, UK

Affiliation:
(1) Nuffield Department of Anaesthetics, Oxford University Hospitals NHS Trust, Oxford, UK

*Corresponding author: sorenki@gmail.com, +44(0)7707838060

Miguel Trelles, PhD
Address:
Rue de l'Arbre Benit 46, 1050 Bruxelles, Belgique

Affiliation:
(1) Médecins sans Frontières
(2) Medical Department – Operational Centre Brussels

Elie Ngowa Bakebaanitsa, Diplôme de Anesthésie et Réanimation
Address:
l'Hôpital général de reference de masisi, zone de santé de masisi, division provinciale de santé du nord-kivu, Republique Democratique du Congo

Affiliation:
(1) Médecins sans Frontières
(2) Masisi referral hospital, Masisi – MSF Democratic Republic of the Congo mission

Longin Hagabimana, Diplôme de Licence en Anesthésie-Réanimation
Address:
L'Hôpital Arche Médical de Kigobe, Bujumbura, Burundi

Affiliation:
(1) Médecins sans Frontières
(2) Arche trauma hospital, Bujumbura – MSF Burundi mission

Abdul Momen, MD
Address:
Khost Maternity Hospital, Khost, Afghanistan

Affiliation:
(1) Médecins sans Frontières
(2) Khost maternity, Khost – MSF Afghanistan mission



For peer review only - http://bmjopen.bmj.com/site/about/guidelines.xhtml

Rahmatullah Helmand, MD
Address:
Jamhoriat Hospital, Chackin Street, New City, Kabul, Afganistan

Affiliation:
(1) Médecins sans Frontières
(2) Ahmad Shah Baba hospital, Kabul – MSF Afghanistan mission

Carline Saint Victor, Certificat en soins anesthésiques et réanimation
Address:
Maternité Isai Jeanty (Hopital Chancerelles), Boulevard Jean-Jacques Dessalines, Port-au-Prince, Haiti

Affiliation:
(1) Médecins sans Frontières
(2) Tabarre trauma hospital, Port-au-Prince – MSF Haiti mission

Khalid Shah, Post RN Dip (Anesthesia)
Address:
DHQ Hospital Timergara, Lower Dir, Khyber Pakhtunkhwa, Pakistan

Affiliations:
(1) Médecins sans Frontières
(2) Timurgara district headquarter hospital – MSF Pakistan mission

Adolphe Masu, MMed
Address:
Avenue Tilipiers number 30, Quartier les Volcans, Ville de Goma, North Kivu province, Republique Democratique du Congo

Affiliations:
(1) Médecins sans Frontières
(2) Arche trauma hospital, Bujumbura – MSF Burundi mission
(3) Khost maternity, Khost – MSF Afghanistan mission
(4) Castors maternity, Bangui – MSF Central African Republic mission

Judith Kendell, MBBS
Address:
Médecins sans Frontières, Rue de l'Arbre Bénit 46, 1050 Bruxelles, Belgique

Affiliation:
(1) Médecins sans Frontières
(2) Medical Department – Operational Centre Brussels


For peer review only - http://bmjopen.bmj.com/site/about/guidelines.xhtml
For peer review only

Hilary Edgcombe, BM BCh FRCA
Address:
Nuffield Department of Anaesthetics, Level 2, John Radcliffe Hospital, Headley Way OX3 9DU

Affiliation:
(1) Nuffield Department of Anaesthetics, Oxford University Hospitals NHS Trust, Oxford, UK

Mike English, FMedSci
Address:
Centre for Tropical Medicine and Global Health, University of Oxford, Old Road Campus, Roosevelt Drive, Oxford OX3 7FZ, UK

Affiliation:
(1) KEMRI-Wellcome Trust Research Programme, Nairobi, Kenya
(2) Centre for Tropical Medicine and Global Health, University of Oxford, Oxford, UK

**Key words:**

Global health
Humanitarian aid
Anaesthesia
Task-shifting and sharing

Word Count (excluding title page, abstract, references, tables, figures): 3371 words

For peer review only

**Abstract**

**Objective:**

To describe the extent to which different categories of anaesthesia provider are used in humanitarian surgical projects and to explore the volume and nature of their surgical workload.

**Design:**

Descriptive analysis using 10 years (2008-2017) of routine case-level data linked with routine program-level data from surgical projects run exclusively by MSF-Operational Centre Brussels (MSF-OCB).

**Setting:**

Projects were in contexts of natural disaster (ND, entire expatriate team deployed by MSF-OCB), active conflict (AC), and stable health care gaps (HG). In AC and HG settings MSF-OCB support pre-existing local facilities. Hospital facilities ranged from basic health centres with surgical capabilities to tertiary referral centres.

**Participants:**

The full dataset included 178,814 surgical cases. These were categorised by most senior anaesthetic provider for the project, according to qualification: Specialist physician anaesthesiologists, qualified nurse anaesthetists, and uncertified anaesthesia providers.

**Primary Outcome Measure:**

Volume and nature of surgical workload of different anaesthesia providers.

**Results:**

Full routine data were available for 173,084 cases (96.8%): 2,518 in ND, 42,225 in AC, 126,936 in HG. Anaesthesia was predominantly led by physician anaesthesiologists (100% in ND, 66% in AC and HG), then nurse anaesthetists (19% in AC and HG) or uncertified anaesthesia providers (15% in AC and HG). Across all settings and provider groups, patients were mostly healthy young adults (median age range 24-27 years), with predominantly females in HG contexts, and males in AC contexts. Intra-operative mortality was 0.3% for physician anaesthesiologists, 0.1-0.2% for nurse anaesthetists, and 0.2-0.3% for uncertified anaesthetic providers.

**Conclusion**:

Our findings contribute to existing knowledge of the nature of anaesthetic provision in humanitarian settings, whilst demonstrating the value of high quality, routine data collection at scale in this sector. These data offer a strong foundation to further evaluation of perioperative outcomes associated with different models of humanitarian anaesthetic provision.



For peer review only - http://bmjopen.bmj.com/site/about/guidelines.xhtml

**Article Summary**

**Strengths and limitations of this study**

- This is the largest study detailing how anaesthetic task sharing and shifting is employed in the humanitarian sector
- Additionally, we believe this is the first study to describe the extent of the presence and caseload of uncertified anaesthetic providers in humanitarian surgical projects
- Due to the nature of the linked data, we were unable to connect anaesthetic provider with individual operations. Therefore, to limit the misclassification bias, we do not ascribe a provider to each case, but rather describe the most senior provider available in the surgical project (the 'anaesthetic lead')

For peer review only


For peer review only - http://bmjopen.bmj.com/site/about/guidelines.xhtml

## INTRODUCTION

Globally there is a large unmet surgical need. Low and middle-income countries (LMIC) are disproportionately affected by gaps in health care provision, with an estimated 90% of patients in these countries unable to access basic surgical care.[1] The burden is increased and access further reduced in crisis situations, caused by conflict or natural disasters.[2] To address these imbalances, Médecins Sans Frontières (MSF, also known as Doctors without Borders) provide humanitarian surgical assistance based on the needs of affected populations through one or more of their five operational centres, one of which is Operational Centre Brussels (MSF-OCB).

There is an increasing body of literature outlining the surgical needs of populations in humanitarian settings;[3–6] further, the recognition that the humanitarian sector is not immune from the need to demonstrate safe surgical care has led to calls for more robust outcome data and clearer accountability.[7–9] Only few studies, limited by small study size and limited external validity, have addressed the composition of the surgical workforce employed by humanitarian organisations.[10,11] Therefore, there is inadequate published data on whether different anaesthesia providers (e.g. physician, nurse, or other health care provider) are employed in different settings, and to what extent there is a physician expatriate presence within the team. In order to comment on outcomes and identify areas where practice can be improved, it is essential to know who provides the care and if there is any learning that can be derived from their practice.

The objective of this study is to describe the extent to which different categories of anaesthesia provider are used in humanitarian surgical projects and to explore the volume and nature of their surgical workload.

## METHODS

The study protocol was submitted to the Oxford Tropical Research Ethics Committee who granted ethical exemption. The study also fulfilled the exemption criteria set by the MSF Ethics Review Board for *a posteriori* analyses of routinely collected clinical data and thus did not require MSF ERB review. It was conducted with permission from Medical Director, MSF-OCB. This exemption did not allow country/site specific detail to be included, therefore we aggregate data within the World Health Organisation (WHO) regional groupings.[12] The findings are reported in accordance with RECORD, the extended STROBE statement on routinely collected data.[13]

### Study design

This was a descriptive study of routine data collected between January 2008 and December 2017. We excluded any incomplete data and data from surgical projects where MSF-OCB were collaborating with other MSF operational centres or local governments, as we were unable to account for workforce or resources made available by others than MSF-OCB.

We linked three sources of data (see figure 1). 1) Case-level routine surgical surveillance data were recorded by theatre staff in logbooks on-site, then transcribed onto an Excel spreadsheet, and finally transferred to Brussels on a monthly basis where they were reviewed and any missing or extraneous data was queried with the local teams. 2) Program-level data, available from MT (head of the Surgical, Anaesthesia, Gynaecology, and Emergency Medicine unit during this period) were reviewed. 3) End of deployment reports written by expatriate physician anaesthesiologists were reviewed to fill gaps in data from the case-level



For peer review only - http://bmjopen.bmj.com/site/about/guidelines.xhtml

data. Data were de-identified at point of data collection, and were only accessed by SK, MT and JK. Any data shared with the remaining co-authors were fully anonymized.

**Setting and anaesthesia providers**

Three different project setting types were identified: 1) regions recently affected by sudden onset natural disasters (ND) where MSF deployed an entire expatriate surgical team in accordance with WHO minimum requirements,[14] 2) active armed conflict (AC) situations and 3) stable situations where MSF supported a pre-existing local facility to address health-care gaps (HG), which existed for a variety of reasons, including the aftermath of natural disasters or armed conflict.

The setup and duration of surgical projects varied. Some projects were intended to operate only for a short period, either within existing local infrastructure or through fully self-contained surgical platforms. Other projects were set up to serve for a longer period or evolved over time into a fully functioning hospital with ability to provide complex care provision. The different hospital types are described in detail in the appendix (appendix, table 1). The setup was not dictated by the setting, and could change over the course of a project. During the 10 years studied, anaesthesia provision was led by one of the following: a) specialist physician anaesthesiologists, either local or expatriate (from both high and low income settings) doctors with qualifications in anaesthesia, b) nurse anaesthetists, either local or expatriate (predominantly from low income settings) nurses or other non-physician clinical cadres with formal training and qualification in anaesthesia in their country of origin, or c) uncertified anaesthesia providers, local nurses or allied health care professionals with a broad range of different levels of experience in anaesthesia provision but without a formal qualification who received on-the-job training only. The MSF-OCB anaesthesia referent assesses the provider requirement for each location based on expected workload, job description and staff availability. For example, if a project is expected to have a low workload, nurse anaesthetists are either recruited locally or, if they are senior providers, sent over as expatriates from MSF-OCB surgical projects in other countries. In situations where MSF-OCB are unable to source qualified staff for a surgical project, they may hire the existing local uncertified anaesthesia providers, who will all receive on-the-job training by MSF and supervision by expatriate physician anaesthesiologists for a trial period. These situations should result in uncertified anaesthesia providers working in settings with a low workload and with distant supervision available from a nearby hospital with MSF-OCB involvement where anaesthesia is led by an expatriate physician anaesthesiologist. All MSF surgical projects have standardised anaesthetic equipment and medications, as described elsewhere.[5]

**Variables and bias**

Different variables were retrieved from the three different data sources. From the routine case-level data (and end of deployment reports) we identified patient variables (including age and sex), surgical and anaesthetic variables (including type of surgery, type of anaesthesia), and geographic location of the cases done. From the program-level data we obtained additional surgical and anaesthetic variables (including provider level of training, presence of expatriate), and location variables (including project setting, type of hospital). A detailed description of all variables used is available in the appendix, table 1.



For peer review only - http://bmjopen.bmj.com/site/about/guidelines.xhtml

The use of routine surveillance data puts the study at risk of selection bias, which may risk under-reporting by some providers (e.g. expatriates visiting for short periods who may be unfamiliar with the data collection tool, or staff who for whatever reason choose not to document cases) or in busy settings (e.g. high workload or strained workforce). While we cannot account for surgical cases not recorded in the first place, we therefore explored data excluded due to incompleteness to assess similarity to the included data.
Furthermore, it should be noted that provider data were available showing the most senior provider present for each project, not per case (and for expatriates, was updated monthly during a project). This puts the study at risk of misclassification bias regarding the anaesthesia providers in favour of the most senior team member regardless of their presence in theatre. Additionally, it would be easy to overrepresent the case-level involvement of physician anaesthesiologists (especially when they are present as expatriates, as they might be more restricted in their movement and have additional non-clinical commitments). We therefore present data according to the most senior provider present on the project in a given month (the anaesthetic 'lead'). We also note which projects had a visiting expatriate physician anaesthesiologist present.

**Statistical analysis**
Data were collected and linked in Excel (2016) and data cleaning and analysis was performed in R 3.6. Continuous data were assessed for normality, and no parametric data were identified. For non-parametric continuous and numeric ordinal data, median, interquartile (IQR) and full range were reported. For categorical variables, the raw counts were reported. We stratified our analysis according to the settings identified, as they might influence the extent and pattern by which different anaesthetic providers were deployed.
However, data from surgical projects in the WHO South East Asia region and in ND settings were described separately due to their small numbers and being separate from the dominant regions (see appendix, tables 2 and 3).

**Patient and public involvement**
There was no involvement of patients or the public in the development or execution of this study.

**RESULTS**

**General findings**
Over the 10 years a total of 173,084 cases had full routine data collected (96.8% of all cases) across 23 countries and 52 different locations (see figure 1). The majority of cases occurred in HG settings, and in the WHO Africa region (see figure 2). Surgical projects in settings of ND represented 3,108 cases (less than 2% of the total number of operations over the time period) and a total duration of 40 project-months over 5 sites; anaesthesia care in the ND setting was exclusively led by physician anaesthesiologists (see appendix, table 3).

Overall the shortest surgical project lasted a month, and the longest lasted beyond the 10 years covered by this study (see figure 3). Surgical projects in HG settings stayed open for longer (median 866 days, IQR 360.25-1900 days) than projects in AC and ND settings (287.5, 173-498.25 days and 210, 122-308 days, respectively). The workload within each


For peer review only - http://bmjopen.bmj.com/site/about/guidelines.xhtml

project varied widely, with 31 projects accounting for 5.1% of all cases, and four projects accounting for 47.6% (see figure 3A).

Of the four biggest projects, anaesthesia for two projects was exclusively physician anaesthesiologist-led (one in the WHO Eastern Mediterranean region in an AC setting, the other in the WHO Americas region in a HG setting). The third project was predominantly physician anaesthesiologist-led (in the WHO Eastern Mediterranean region) progressing from an initial AC to become a stable HG setting. The last was predominantly uncertified anaesthesia provider-led with a periodic presence of expatriate physician anaesthesiologists (in the WHO Africa region, starting in AC and then becoming a stable HG setting). Data for these four major projects followed a similar pattern of distribution (in terms of case and program-level data) to the remaining dataset of all other projects, and have therefore been included in the findings below.

**Program-level provider findings**

Most surgical projects (23/28 in AC, 25/32 in HG, and all 5 in ND) included a period of anaesthesia provision led by physician anaesthesiologists (see figure 3B and table 1A). Anaesthesia in any setting with sole trauma care was mostly led by physician anaesthesiologists (see table 1A). If anaesthesia provision in a project was not fully physician anaesthesiologist-led, the pattern of their presence in most cases involved short periods (usually around 3 months) over the course of the surgical project, mostly towards the start of the project (see figure 3B). Overall, a physician anaesthesiologist was identified as present for 737 (49%) project-months in AC and HG (see table 1B). However, in these settings more than 66% of cases overall were conducted during periods where physician anaesthesiologist were present in the projects (80% of cases in AC and 60% of cases in HG).
When there was not a physician anaesthesiologist attached to a project, anaesthesia was most commonly led by nurse anaesthetists in the HG setting and most commonly led by uncertified anaesthesia providers in the AC setting.

For peer review only


For peer review only - http://bmjopen.bmj.com/site/about/guidelines.xhtml

**Table 1A: Programme-level descriptive table according to number of surgical projects in different settings**

| | Physician anaesthesiologist led | Nurse anaesthetist led | Unqualified anaesthetic provider led | Physician anaesthesiologist led | Nurse anaesthetist led | Unqualified anaesthetic provider led |
|---|---|---|---|---|---|---|
| | CONFLICT (SURGICAL PROJECTS = 28) | | | HEALTH CARE GAP (SURGICAL PROJECTS = 32)[a] | | |
| **Number of surgical projects involved in at any point[b]** | 23 | 14 | 12 | 25 | 17 | 12 |
| **Type of hospital[c] in surgical project, No. surgical projects involved in at any point[d]** | | | | | | |
| **Sole remit hospital** | 5 | 2 | 1 | 13 | 7 | 1 |
| **Referral hospital** | 6 | 5 | 2 | 7 | 5 | 2 |
| **District hospital** | 11 | 4 | 8 | 7 | 4 | 7 |
| **Health centre** | 1 | 3 | 1 | 0 | 1 | 2 |
| **Type of surgical care performed in project, No. surgical projects involved in at any point[d]** | | | | | | |
| **Emergency only** | 9 | 8 | 5 | 3 | 4 | 4 |
| **Capacity to perform both emergency and elective surgery** | 8 | 4 | 6 | 11 | 5 | 6 |
| **Maternity care only** | 1 | 1 | 1 | 9 | 6 | 1 |
| **Trauma care only** | 4 | 1 | 0 | 2 | 0 | 0 |

| | | | | | | | | |
|---|---|---|---|---|---|---|---|---|
| **Other specific care provision**[e] | 1[1] | 0 | 0 | | 2[2,3] | 1[3] | 1[4] | |

**Table 1B: Programme-level descriptive table according to months of activity in different settings**

| | **Physician anaesthesiologist led** | **Nurse anaesthetist led** | **Unqualified anaesthetic provider led** | **All conflict missions** | **Physician anaesthesiologist led** | **Nurse anaesthetist led** | **Unqualified anaesthetic provider led** | **All health care gap missions** |
|---|---|---|---|---|---|---|---|---|
| | **CONFLICT (SURGICAL PROJECTS = 28)** | | | | **HEALTH CARE GAP (SURGICAL PROJECTS = 32)**[a] | | | |
| **Number of months active in any mission** | 235 | 75 | 94 | **404** | 502 | 429 | 160 | **1091** |
| **Surgical provider, No. months present (% of cohort)** | | | | | | | | |
| **General and specialty surgeon** | 100 (43) | 13 (17) | 0 (0) | **113 (28)** | 260 (52) | 105 (24) | 3 (2) | **368 (34)** |
| **General surgeon only** | 96 (41) | 58 (77) | 20 (20) | **174 (43)** | 115 (23) | 133 (31) | 38 (24) | **286 (26)** |
| **Specialty surgeon only** | 31 (13) | 2 (3) | 4 (4) | **37 (9)** | 103 (21) | 162 (38) | 7 (4) | **272 (25)** |
| **MD**[f] | 8 (3) | 2 (3) | 70 (74) | **80 (20)** | 24 (5) | 29 (7) | 112 (70) | **165 (15)** |

[a] South East Asia Region contributed such a small proportion to missions in "health care gap" settings (2 missions, 815 cases or <1%), that they have been excluded and instead described in appendix, table 2.

[b] Surgical projects can have anaesthesia provision by multiple different providers during the period they are open. Therefore, the rows might add up to more than the total number of projects in each setting.

[c] Definitions of hospitals found in appendix, table 1.

[d] Two surgical projects changed from being able to provide both emergency and elective surgery, to providing solely maternity care. As such, they are counted twice under "type of hospital" and "type of surgical care".

[e] Specific care provision are surgical projects with a specific care remit. This includes [1]wound care, [2]trauma and surgical care, [3]obstetric fistula care, and [4]surgical care of typhoid related complications.

[f] MD = local physician with surgical skills but without a formal surgical qualification.


For peer review only - http://bmjopen.bmj.com/site/about/guidelines.xhtml

**Case-level provider findings**

Case-mix was similar across all lead providers with respect to age (mostly young adults) and underlying health (mostly ASA 1) (see table 2). All providers did predominantly non-elective work, although cases done during nurse anaesthetist-led project-months had a higher proportion of emergency surgery. During these months there was a predominance of caesarean sections in HG settings, though outside this setting the most common type of surgery was minor surgery for all lead providers. The intra-operative mortality was 0.3% and 0.3% in physician anaesthesiologist-led project-months, 0.2% and 0.1% in nurse anaesthetist-led project-months, and 0.3% and 0.2% in uncertified anaesthesia provider-led project-months in AC and HG settings, respectively.

All lead providers made use of the two most common types of anaesthesia: spinal injection alone and general anaesthesia (GA) without intubation or muscle relaxant, which for the most part was ketamine-based. This was done in broadly similar proportions when comparing surgical categories in different settings (as an example, spinal injection and GA without protected airway for caesarean section was 61-70% and 22-36%, respectively in AC, and 78-86% and 6-14%, respectively in HG).

For peer review only

**Table 2: Case-level descriptive table grouped according to setting[a]**

| | Physician anaesthesiologist led | Nurse anaesthetist led | Unqualified anaesthetic provider led | Physician anaesthesiologist led | Nurse anaesthetist led | Unqualified anaesthetic provider led |
|---|---|---|---|---|---|---|
| | CONFLICT (SURGICAL PROJECTS = 28, N = 42,225) | | | HEALTH CARE GAP (SURGICAL PROJECTS = 32, N = 126,936)[b] | | |
| Number of all surgical episodes, No. | 33763 | 3798 | 4664 | 78126 | 28559 | 20251 |
| **Patient demographics** | | | | | | |
| Female, No. (%) | 12424 (37) | 1888 (50) | 2237 (48) | 38919 (50) | 22439 (79) | 12834 (63) |
| Median age, years (IQR, [range]) | 23 (15-33, [1 day old-105]) | 25 (16-34, [2 day old-90]) | 23 (18-30, [3 day old-94]) | 28 (19-37, [1 day old-102]) | 26 (20-34, [1 day old-98]) | 25 (16-35, [1 day old-96]) |
| ASA, value (IQR, [range]) | 1 (1-2, [1-5]) | 2 (1-2, [1-5]) | 1 (1-2, [1-5]) | 1 (1-2, [1-5]) | 1 (1-2, [1-5]) | 1 (1-2, [1-5]) |
| Cause of hospitalisation, No. %: | | | | | | |
| • Trauma (intentional or unintentional) | 21968 (65) | 1384 (36) | 2366 (51) | 42454 (54) | 2850 (10) | 6303 (31) |
| • Obstetric | 6642 (20) | 1073 (28) | 1295 (28) | 20387 (26) | 18270 (64) | 7211 (36) |
| • Other[c] | 5153 (15) | 1341 (35) | 1003 (22) | 15285 (20) | 7439 (26) | 6737 (33) |
| **Surgical demographics** | | | | | | |
| Urgency, No. (%) | | | | | | |
| • Emergent | 14344 (42) | 2203 (58) | 2581 (55) | 37234 (48) | 19922 (70) | 9221 (46) |
| • Urgent | 18091 (54) | 1115 (29) | 1961 (42) | 34771 (45) | 4781 (17) | 8699 (43) |
| • Elective | 1328 (4) | 480 (13) | 122 (3) | 6121 (8) | 3856 (14) | 2331 (12) |
| Proportion of cases from initial presentation, n (%) | 20079 (59) | 3053 (80) | 3588 (77) | 51493 (66) | 25597 (90) | 14492 (72) |
| Median time in theatre, minutes (IQR, [range]) | 50 (30-70, [7-710]) | 50 (35-70, [15-356]) | 45 (35-65, [10-360]) | 60 (35-90, [10-870]) | 60 (50-80, [10-1140]) | 50 (30-70, [5-460]) |
| **Main categories of surgery, No. (%)[d]** | | | | | | |
| Minor surgery | 20670 (61) | 1688 (44) | 3019 (65) | 37419 (48) | 6798 (24) | 9906 (49) |

| | | | | | | |
|---|---|---|---|---|---|---|
| **Caesarean section** | 4758 (14) | 891 (23) | 884 (19) | 16138 (21) | 13336 (47) | 6259 (31) |
| **Visceral surgery** | 3709 (11) | 949 (25) | 555 (12) | 11109 (14) | 4856 (17) | 2922 (14) |
| **Orthopaedic surgery** | 3372 (10) | 90 (2) | 48 (1) | 9408 (12) | 151 (1) | 328 (2) |
| **Obstetric & gynaecological surgery (excl. caesarean section)** | 802 (2) | 122 (3) | 139 (3) | 3226 (4) | 3147 (11) | 756 (4) |
| **Specialties[e]** | 452 (1) | 58 (2) | 19 (0) | 826 (1) | 271 (1) | 80 (0) |
| **Intra-operative mortality, No. (%)** | | | | | | |
| **For all cases** | 102 (0·3) | 7 (0·2) | 16 (0·3) | 204 (0·3) | 31 (0·1) | 31 (0·2) |

[a] Percentages have been rounded to nearest full digit, and might not add up to 100%.

[b] South East Asia Region contributed such a small proportion to missions in “health care gap” settings (2 missions, 815 cases or <1%), that they have been excluded and instead described in the appendix, table 2.

[c] “Other” causes of hospitalisation include: tropical disease related, tumours, non-tumour related obstruction, and complications from traditional medical practices.

[d] The surgical procedures included in each grouping can be found in the appendix, table 4.

[e] Specialties encompass (total number of cases across whole dataset): Urology (726), vascular surgery (355), plastic and reconstructive surgery (144), ENT surgery (116), neurosurgery (115), surgery within thoracic cavity (108), maxillofacial surgery (61), and other forms of specialised surgical care that does not fall into the aforementioned categories (109).


For peer review only - http://bmjopen.bmj.com/site/about/guidelines.xhtml

**Missing data**

The cases excluded due to missing variables (5730, 3.2%) are predominantly from the early years. The three most common variables with missing data was ASA score (3232 missing), intra-operative mortality (2154), and time in theatre (1922) (see appendix, missing data table 1). The data with missing intra-operative mortality was exclusively from 2008, and were predominantly from two projects in the WHO Africa region where the bulk of the work was elective surgery for training purposes. Eight surgical projects were completely excluded (7 in health care gap settings, 1 that was in both natural disaster settings and health care gap settings, see appendix, missing data figure 1), all with a caseload of less than 100 operations and a short period of activity. The missing data were predominantly from projects with uncertified anaesthesia provider-led or physician anaesthesiologist expatriate led provision. This suggest the data were not missing completely at random and may risk introducing bias, although they comprised a small overall proportion of cases and available variables suggest the excluded cases were similar to the analysed dataset (see appendix, missing data table 2).

**DISCUSSION**

This is the largest observational study published from a humanitarian organisation describing the types of anaesthesia providers employed and the pattern of their work in a number of different settings. While not all humanitarian organisations (and MSF operational centres) operate in the same way as MSF-OCB, this study provides useful insights that may contribute towards their operational strategies.

Over 10 years of surgical activity by MSF-OCB, we found that anaesthesia provision was led by physician anaesthesiologists during 66% of all cases in HG and AC settings (bearing in mind physician anaesthesiologist-led does not mean physician anaesthesiologists administered the anaesthesia) with nurse anaesthetist-led provision accounting for 19% and uncertified anaesthesia provider-led provision accounting for 15% of cases. While there was variation in the surgical caseload between provider types (physician anaesthesiologists were more commonly attached to projects with trauma-related surgery, while nurse anaesthetists were more commonly the most senior anaesthetic provider in projects with high numbers of obstetric surgery), all providers led during surgery on both very sick (ASA grade 5) and very young patients (aged only a few days). In locations with uncertified anaesthesia provider-led anaesthesia, which was predominantly in the WHO Africa region, there was also a reduced presence of specialized surgical providers and expatriate involvement, despite the patient profile and surgical caseload being largely similar to that encountered in physician anaesthesiologist-led surgical projects in similar settings.

MSF tries to avoid employing uncertified anaesthesia providers, and they continue to evaluate means of mitigating this risk. However, a set of unique circumstances makes it unavoidable on occasion: 1) MSF, like many humanitarian organisations, operate predominantly in locations where there is a pre-existing anaesthesia workforce shortage,[15] and often in situations where this shortage may be exacerbated due to armed conflict or population displacement. 2) Expatriate staff are not always available, as MSF only deploy senior qualified anaesthesiologists as their expatriates, and it may not be possible for them to take time away from work at short notice. 3) Even if expatriate staff are available, in many


For peer review only - http://bmjopen.bmj.com/site/about/guidelines.xhtml

contexts they have become deliberate targets. This has led to more cautious deployment of expatriate personnel into volatile settings.[16]

In this study we report briefly on intra-operative mortality. Rates are comparable across the different lead providers and similar to other observational data from LMICs[17–21] and some humanitarian organisations (including other MSF operational centres),[4,22,23] while higher than other humanitarian organisations.[24,25] Such data must be interpreted cautiously as they should ideally be adjusted more fully for case-mix and severity. Further, most mortality related to surgery occurs in the days following surgery and not in theatre,[23,26,27] and these data are not available as part of the routine data we analysed. While a more appropriate and widely recognized measure of surgical outcomes is perioperative mortality, which is advocated by both the Lancet Commission on Global Surgery and the World Health Organisation.[28,29] we were unable to report this. Further research into surgical outcomes in the humanitarian setting, which includes perioperative mortality and the incidence of post-operative complications and how they might differ between different anaesthesia providers, would be useful to assist organisations in providing safe and efficient anaesthesia in resource limited situations.

**Limitations**

Data quality is a known issue when using surveillance data, and the occasionally unpredictable nature of working in humanitarian settings means there is a risk of further decline in quality. Due to the rigor in data monitoring centrally by MSF-OCB on a regular basis as described in the methods section, much has been done to minimize both missing data and improve the quality of the collected dataset. Our approach does have a particular risk of misclassification related to expatriate physician presence. Cases or projects could have been identified as ‘physician anaesthesiologist-led’, but the physician anaesthesiologist may not actually have been in the operating room for a variety of reasons including overseeing multiple theatres, or curfew and security concerns. Such misclassification could underrepresent the proportion of work where non-physicians were effectively sole providers. Our results therefore likely present a conservative estimate of the care provided by nurse anaesthetist and uncertified anaesthesia provider. Finally, it is important to note that some projects had started before the start of routine data collection in 2008. Projects with expatriate physician anaesthesiologists providing on-the-job training for uncertified anaesthesia providers in the period before 2008 will not be reflected in our dataset.

**CONCLUSION**

The majority of MSF anaesthesia care is done in teams where there are physician anaesthesiologists available. In conflict and healthcare gap settings, nurse anaesthetists and uncertified anaesthesia providers are also major providers, and all providers encounter both the extremely young and the extremely sick in these challenging contexts. This study show that the humanitarian sector has considerable experience with task sharing and shifting, and with further study of perioperative outcomes in these circumstances, lessons may be learned for more stable settings that could contribute to system strengthening. Despite their limitations routine data are key to monitoring the effectiveness of health systems, including humanitarian care, at scale and the MSF-OCB dataset is an important resource demonstrating that valuable data can be collected even in difficult circumstances. There is a need for wider engagement by the humanitarian community to continue to improve the collection and use of


For peer review only - http://bmjopen.bmj.com/site/about/guidelines.xhtml

valid surgical outcome data. This would promote learning on how to optimise the surgical and anaesthetic workforce and help to ensure safe surgical and anaesthetic care in the humanitarian sector, which could be valuable in many LMIC routine health settings.

## COMPETING INTERESTS

SK received funding from NIHR through their academic clinical fellowship scheme. ME received funding from a Wellcome Trust Senior Fellowship (#207522) as part of an unrelated research grant. All authors except from SK, ME, and HE are employed by MSF-OCB.

## AUTHOR CONTRIBUTIONS

SK helped conceive the study design, analyse the data, interpret the data, write the initial draft of the manuscript, and edit the manuscript. MT helped conceive the study design, collect the data, interpret the data, and edit the manuscript. ENB, LH, AM, RH, CStV, KS, AM, and SG helped collect the data and edit the manuscript. JK helped collect the data, interpret the data and edit the manuscript. HE and ME helped conceive the study design, interpret the data, and edit the manuscript.

## DATA SHARING STATEMENT

The data used in the study was provided by MT (SAGE Coordinator at MSF-Brussels at the time data was obtained), and contains de-identified case-level routine surgical surveillance data and programme-level data. All relevant data is available in the tables, figures and appendix.

## FUNDING

This research received no specific grant from any funding agency in the public, commercial or not-for-profit sectors.

## LEGEND OF FIGURES

- Figure 1: Flow diagram showing inclusion/exclusion of data and points of data linkage

- Figure 2: World maps showing number of (A) Surgical projects and (B) Surgical cases in each WHO region in settings of (1) conflict, (2) health care gaps, and (3) natural disasters

- Figure 3: Timelines showing duration and point in time all included surgical projects were active

## REFERENCES

1 Meara JG, Leather AJM, Hagander L, *et al.* Global Surgery 2030: Evidence and solutions for achieving health, welfare, and economic development. *Lancet* 2015;**386**:569–624. doi:10.1016/S0140-6736(15)60160-X
2 Craven RM. Managing anaesthetic provision for global disasters. *Br J Anaesth*


For peer review only - http://bmjopen.bmj.com/site/about/guidelines.xhtml

2017;**119**:i126–34. doi:10.1093/bja/aex353
3 White MC, Randall K, Avara E, *et al.* Clinical Outcome, Social Impact and Patient Expectation: a Purposive Sampling Pilot Evaluation of Patients in Benin Seven Years After Surgery. *World J Surg* 2018;**42**:1254–61. doi:10.1007/s00268-017-4296-9
4 Trudeau MON, Baron E, Hérard P, *et al.* Surgical care of pediatric patients in the humanitarian setting: The médecins sans frontières experience, 2012-2013. *JAMA Surg* 2015;**150**:1080–5. doi:10.1001/jamasurg.2015.1928
5 Ariyo P, Trelles M, Helmand R, *et al.* Providing Anesthesia Care in Resource-limited Settings: A 6-year Analysis of Anesthesia Services Provided at Médecins Sans Frontières Facilities. *Anesthesiology* 2016;**124**:561–9.
6 Chu KM, Ford N, Trelles M. Operative Mortality in Resource-Limited Settings: The Experience of Medecins Sans Frontières in 13 Countries. *Arch Surg* 2010;**145**:721–5.
7 Zitzman E, Berkley H, Jindal RM. Accountability in global surgery missions. *BMJ Glob Heal* 2018;**3**:e001025. doi:10.1136/bmjgh-2018-001025
8 Shrime MG, Sleemi A, Ravilla TD. Charitable platforms in global surgery:A systematic review of their effectiveness, cost-effectiveness, sustainability, and role training. *World J Surg* 2015;**39**:10–20. doi:10.1007/s00268-014-2516-0
9 Hendriks TCC, Botman M, Rahmee CNS, *et al.* Impact of short-term reconstructive surgical missions: A systematic review. *BMJ Glob Heal* 2019;**4**. doi:10.1136/bmjgh-2018-001176
10 Chu KM, Ford NP, Trelles M. Providing surgical care in Somalia: A model of task shifting. *Confl Health* 2011;**5**:12. doi:10.1186/1752-1505-5-12
11 Plecker E De, Zachariah R, Kumar AM V., *et al.* Emergency obstetric care in a rural district of Burundi: What are the surgical needs? *PLoS One* 2017;**12**:e0170882. doi:10.1371/journal.pone.0170882
12 World Health Organisation. Regional offices. https://www.who.int/about/who-we-are/regional-offices (accessed 4 Jul 2019).
13 Benchimol EI, Smeeth L, Guttmann A, *et al.* The REporting of studies Conducted using Observational Routinely-collected health Data (RECORD) statement. *PLoS Med* 2015;**12**:e1001885. doi:10.1371/journal.pmed.1001885
14 Foreign Medical Team Working Group. Classification and Minimum Standards for Foreign Medical Teams in Sudden Onset. Geneva: 2013.
15 Kempthorne P, Morriss WW, Mellin-Olsen J, *et al.* The WFSA Global Anesthesia Workforce Survey. *Anesth Analg* 2017;**125**:981–90. doi:10.1213/ANE.0000000000002258
16 ICRC. Health Care in Danger: A sixteen-country study. Geneva: 2011.
17 Kwari YD, Bello MR, Eni UE. Pattern of perioperative cardiac arrests at University of Maiduguri Teaching Hospital. *Niger J Med J Natl Assoc Resid Dr Niger* 2010;**19**:173–6.
18 Zoumenou E, Gbenou S, Assouto P, *et al.* Pediatric anesthesia in developing countries: experience in the two main university hospitals of Benin in West Africa. *Paediatr Anaesth* 2010;**20**:741–7.
19 Walker IA, Obua AD, Mouton F, *et al.* Paediatric surgery and anaesthesia in south-western Uganda: a cross-sectional survey. *Bull World Health Organ* 2010;**88**:897–906.
20 Rukewe A, Fatiregun A, Adebayo K. Anaesthesia for caesarean deliveries and maternal complications in a Nigerian teaching hospital. *African J Med Med Sci* 2014;**43**:5–10.
21 Rukewe A, Fatiregun A, Osunlaja TO. Cardiac arrest during anesthesia at a university Hospital in Nigeria. *Niger J Clin Pract* 2014;**17**:28–31.
22 Wichlas F, Tsitsilonis S, Guanti MD, *et al.* Orthopedic trauma surgeon in Sierra Leone: how to keep one's head over water. *Arch Orthop Trauma Surg* 2019;**0**:0. doi:10.1007/s00402-019-03171-1
23 Davies JF, Lenglet A, Van Wijhe M, *et al.* Perioperative mortality: Analysis of 3 years of operative data across 7 general surgical projects of Medecins Sans Frontieres in Democratic Republic of Congo, Central African Republic, and South Sudan. *Surgery* 2016;**159**:1269–78. doi:10.1016/j.surg.2015.12.022
24 Fisher QA, Nichols D, Stewart FC, *et al.* Assessing Pediatric Anesthesia Practices for Volunteer Medical Services Abroad. *Anesthesiol J Am Soc Anesthesiol* 2001;**95**:1315–


For peer review only - http://bmjopen.bmj.com/site/about/guidelines.xhtml

22.

25 White MC, Barki BJ, Lerma SA, *et al.* A prospective observational study of anesthesia- related adverse events and postoperative complications occurring during a surgical mission in Madagascar. *Anesth Analg* 2018;**127**:506–12.

26 Biccard BM, Madiba TE, Kluyts H-L, *et al.* Perioperative patient outcomes in the African Surgical Outcomes Study: a 7-day prospective observational cohort study. *Lancet* 2018;**391**:1589–98. doi:10.1016/S0140-6736(18)30001-1

27 Ng-Kamstra JS, Arya S, Greenberg SLM, *et al.* Perioperative mortality rates in low-income and middle-income countries: a systematic review and meta-analysis. *BMJ Glob Heal* 2018;**3**:e000810. doi:10.1136/bmjgh-2018-000810

28 Holmer H, Bekele A, Hagander L, *et al.* Evaluating the collection, comparability and findings of six global surgery indicators. *Br J Surg* 2019;:e138–50. doi:10.1002/bjs.11061

29 World Health Organisation. 2018 Global reference list of 100 core health indicators (plus health-related SDGs). Geneva: 2018.

For peer review only

**Figure 1: Flow diagram showing inclusion/exclusion of data and points of data linkage**

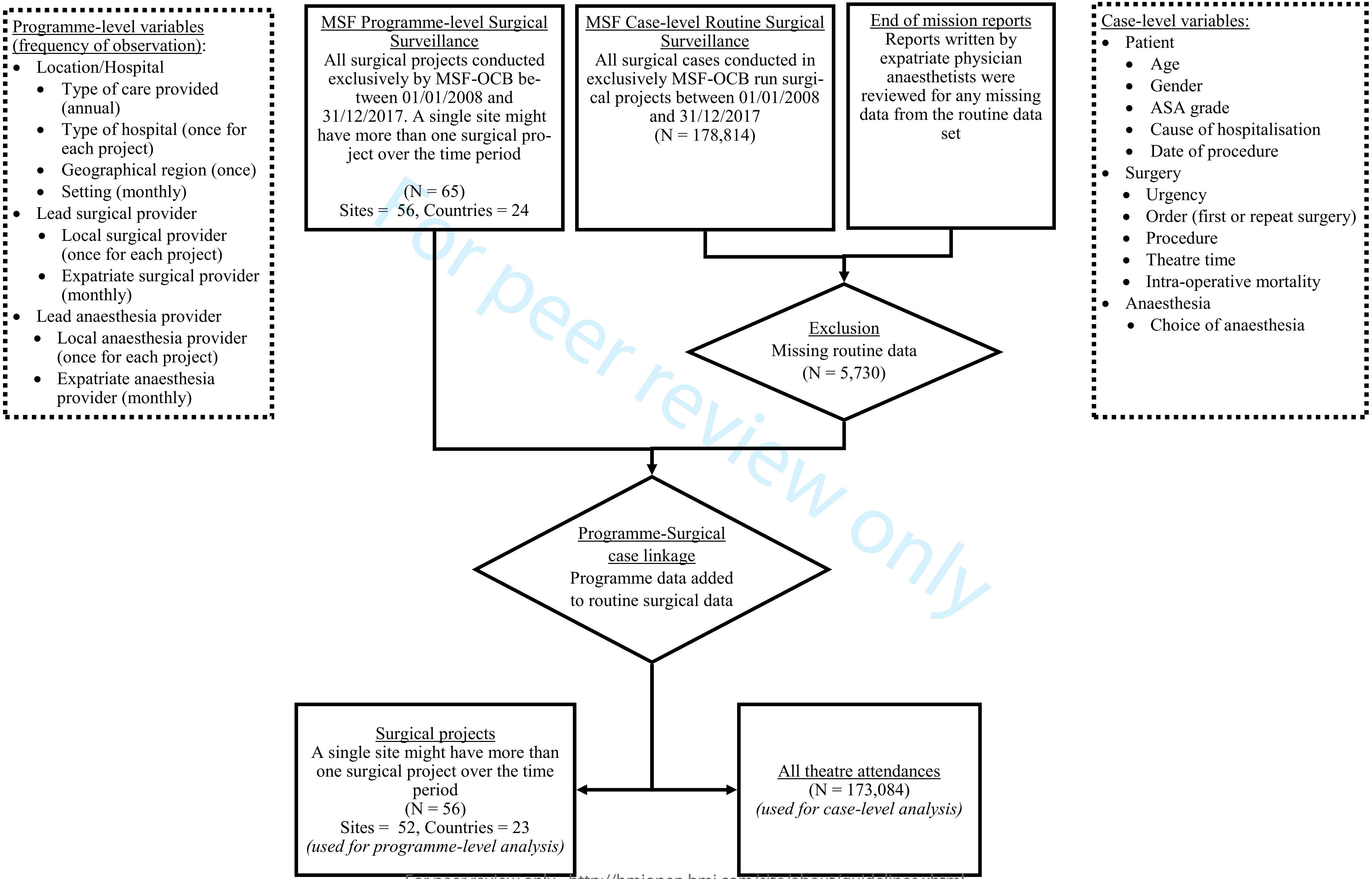


For peer review only

Figure 2: World maps showing number of (A) Surgical projects and (B) Surgical cases in each WHO region in settings of (1) conflict, (2) health care gaps, and (3) natural disasters

**A**

| WHO regions | Conflict setting | Health care gap setting | Natural disaster setting |
|---|---|---|---|
| Africa region | 21 | 21 | 0 |
| Americas region | 0 | 3 | 3 |
| Eastern Mediterranean region | 7 | 8 | 0 |
| South−East Asia region | 0 | 2 | 1 |
| Western Pacific region | 0 | 0 | 1 |

**1** **2** **3**

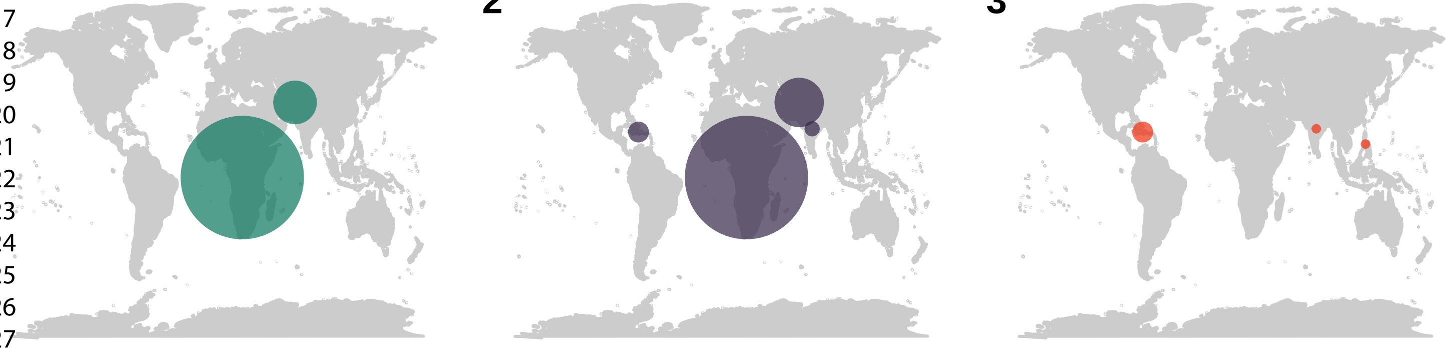

**B**

| WHO regions | Conflict setting | Health care gap setting | Natural disaster setting |
|---|---|---|---|
| Africa region | 16087 | 65551 | 0 |
| Americas region | 0 | 33319 | 2730 |
| Eastern Mediterranean region | 26138 | 28066 | 0 |
| South−East Asia region | 0 | 815 | 89 |
| Western Pacific region | 0 | 0 | 289 |

**1** **2** **3**

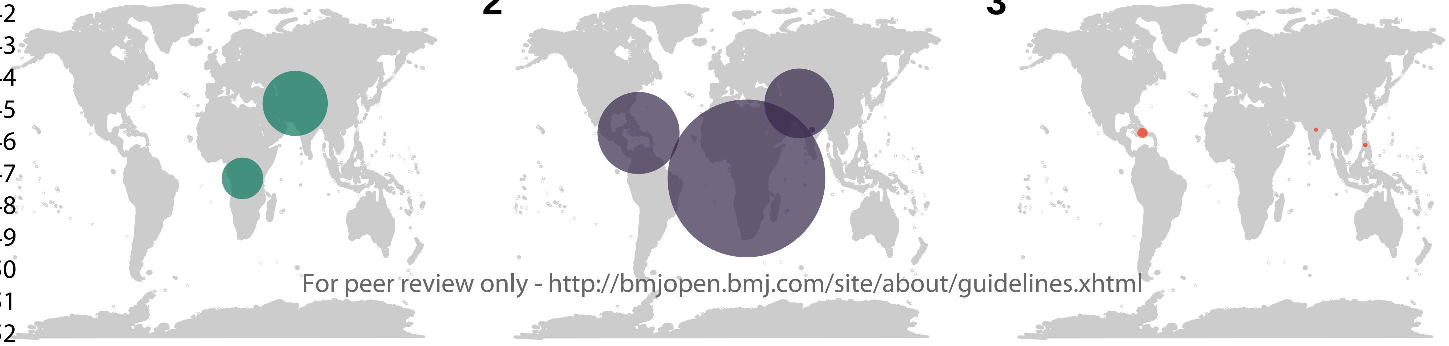

For peer review only - http://bmjopen.bmj.com/site/about/guidelines.xhtml

Figure 3: Timelines showing duration and point in time all included surgical projects were active

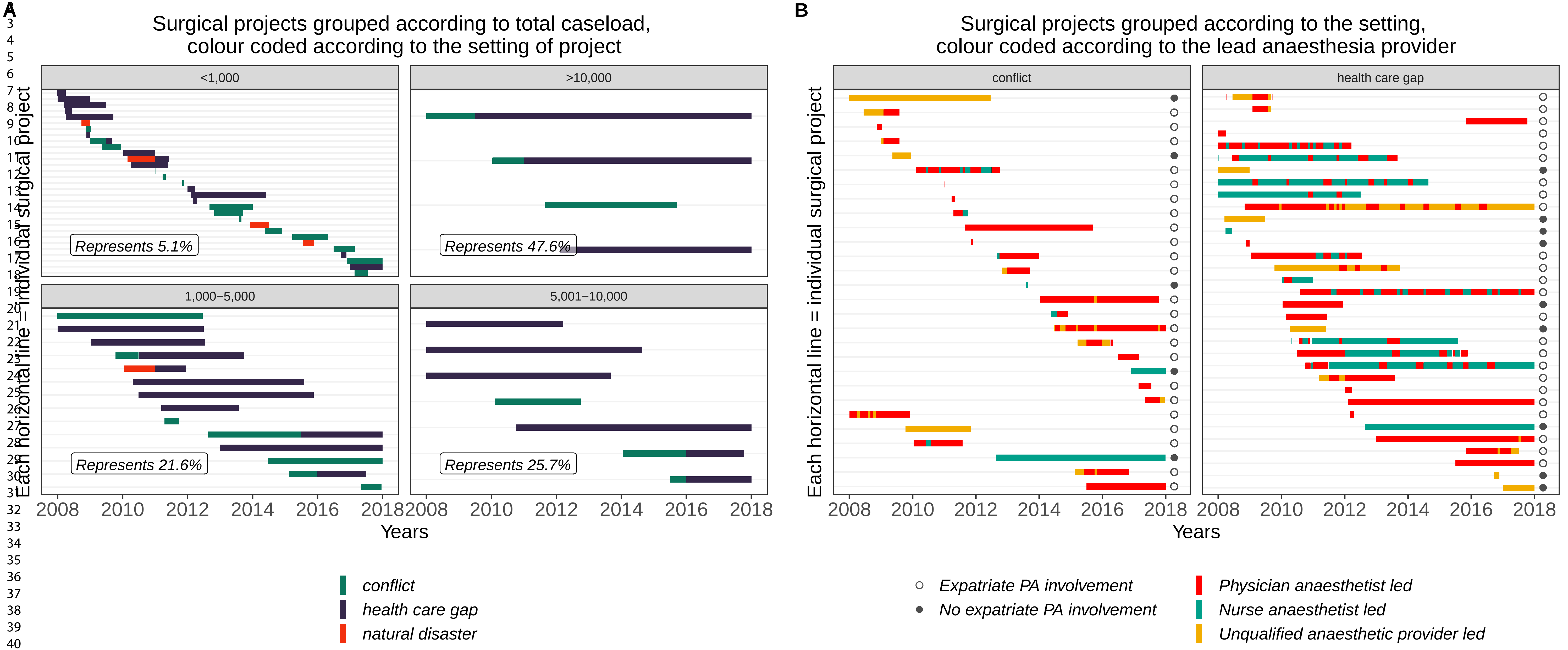




*Excludes periods where projects are run in collaboration with other organisations or local government. Additionally, only data from 2008 till 2017 are included. Therefore, periods with expatriate physician anaesthetist (PA) involvement before then are not reflected here.

# Appendix

Table of Contents

1. Table 1: Variables used in the study
2. Table 2: surgical projects in health care gap settings in the WHO SEA region (2 in total)
3. Table 3: Surgical projects in natural disaster settings (5 in total)
4. Table 4: Surgical groupings as used in main table 2 (case-level data)
5. Missing data

    Table 1: Number of missing values within each variable

    Figure 1: Proportion of missing data in each surgical project

    Table 2: overview of available data

For peer review only

# 1. Table 1: Variables used in the study

<table>
<tr><th>Key characteristic group</th><th>Name of variable</th><th>Type of data</th><th>Description of data</th><th>Source</th></tr>
<tr><td rowspan="5">Patient</td><td>Age</td><td>Continuous</td><td>For patients below the age of 2 (typed in original data collection sheet as days and months), the age has been converted for analysis to a fraction of a year.</td><td>Case-level routine data</td></tr>
<tr><td>Gender</td><td>Categorical (binary)</td><td>Either male or female</td><td>Case-level routine data</td></tr>
<tr><td>Date of procedure</td><td>Continuous</td><td>Date operation took place</td><td>Case-level routine data</td></tr>
<tr><td>ASA grade</td><td>Ordinal</td><td>American Society of Anaesthesiologists physical status classification system (ASA). Discrete numeric scale between 1 and 5 (1 = normal healthy patient, 5 = moribund patient expected not to survive without surgery) of the patient’s physical health prior to surgery.</td><td>Case-level routine data</td></tr>
<tr><td>Cause of hospitalisation</td><td>Categorical (nominal)</td><td>3 letter code used as defined by MSF-OCB operational departmental guideline. A total of 24 codes available, and grouped into 4 distinct categories:<br>- Accidental trauma<br>- Violent trauma<br>- Obstetric<br>- Other (including but not limited to tropical disease, tumours, obstruction)</td><td>Case-level routine data</td></tr>
<tr><td rowspan="4">Surgery</td><td>Urgency</td><td>Categorical (ordinal)</td><td>3 values available, relating to how soon the surgical procedure has to occur:<br>- “Urgent” (labelled “emergent” in manuscript) = requiring immediate surgery<br>- “Delayed” (labelled “urgent” in manuscript) = requiring surgery during current hospital admission<br>- “Planned” (labelled “elective” in manuscript) = elective surgery</td><td>Case-level routine data</td></tr>
<tr><td>Order</td><td>Categorical (ordinal)</td><td>3 codes available, relating to whether patient has had surgery before during admission:<br>- “First” = first time entering theatre<br>- “Unplanned” = unplanned return to theatre<br>- “Re-intervention” = planned return to theatre</td><td>Case-level routine data</td></tr>
<tr><td>Procedure – Main group</td><td>Categorical (nominal)</td><td>2 letter code used as defined by MSF-OCB operational department guidelines. A total of 36 procedures codes available, and grouped into the main categories of surgery. See appendix table 4 for full breakdown.</td><td>Case-level routine data</td></tr>
<tr><td>Lead surgical provider</td><td>Categorical (nominal)</td><td>A merged variable, based on the presence of local and expatriate providers as outlined below. MD signifies a physician without surgical qualifications but with surgical skills.
<table>
<tr><td></td><td>Local provider</td><td>Expatriate provider</td></tr>
<tr><td rowspan="4">General & specialist surgeon</td><td>None</td><td>General & Specialist</td></tr>
<tr><td>MD</td><td>General & Specialist</td></tr>
<tr><td>General</td><td>Specialist</td></tr>
<tr><td>General & Specialist</td><td>Any</td></tr>
<tr><td rowspan="2">Specialist only</td><td>None</td><td>Specialist</td></tr>
<tr><td>MD</td><td>Specialist</td></tr>
</table></td><td>Programme-level routine data (MT)</td></tr>
</table>

<table>
<tr><td></td><td></td><td></td><td><table><tr><td></td><td>Specialist</td><td>Specialist or none</td></tr><tr><td rowspan="3">General only</td><td>None</td><td>General</td></tr><tr><td>MD</td><td>General</td></tr><tr><td>General</td><td>General or none</td></tr><tr><td>MD</td><td>MD</td><td>None</td></tr></table></td><td></td></tr>
<tr><td></td><td>Theatre time (min)</td><td>Continuous</td><td>Time in minutes the patient was occupying theatre. This included anaesthetic and surgical time, as well as any recovery of patient, which occurred in theatre.</td><td>Case-level routine data</td></tr>
<tr><td></td><td>Intra-operative mortality</td><td>Categorical (binary)</td><td>Whether the patient was dead or alive by the time they left recovery. For the purpose of the study, this is considered as intraoperative mortality.</td><td>Case-level routine data</td></tr>
<tr><td rowspan="2">Anaesthesia</td><td>Choice of anaesthesia</td><td>Categorical (nominal)</td><td>List of 7 codes of anaesthesia that can be provided. Only a single code can be used for a surgical procedure:<br>- Local anaesthesia<br>- Regional anaesthesia<br>- Spinal anaesthesia<br>- General anaesthesia without intubation or muscle relaxant<br>- General anaesthesia with intubation and/or muscle relaxant<br>- Combined anaesthesia (if more than one code need to be used, e.g. spinal anaesthesia + general anaesthesia)<br>- Other anaesthesia, e.g. sedation</td><td>Case-level routine data</td></tr>
<tr><td>Lead anaesthesia provider</td><td>Categorical (nominal)</td><td>A merged variable, based on the presence of local and expatriate providers as outlined below.<table><tr><td></td><td>Local provider</td><td>Expatriate provider</td></tr><tr><td rowspan="4">Physician anaesthesiologist (PA) led</td><td>PA</td><td>none</td></tr><tr><td>NA</td><td>PA</td></tr><tr><td>UA</td><td>PA</td></tr><tr><td>None</td><td>PA</td></tr><tr><td rowspan="3">Nurse anaesthetist (NA) led</td><td>NA</td><td>none</td></tr><tr><td>UA</td><td>NA</td></tr><tr><td>None</td><td>NA</td></tr><tr><td>Uncertified anaesthetic provider (UA) led</td><td>UA</td><td>None</td></tr></table></td><td>Programme-level routine data (MT)</td></tr>
<tr><td>Setting</td><td>Type of care provided by hospital</td><td>Categorical (nominal)</td><td>Overall type of provision provided during surgical project, based on MSF-OCB brief. 9 separate categories grouped into 5:<br>- Emergency only<br>- Capacity to perform both emergency and elective surgery<br>- Maternity care only<br>- Trauma care only<br>- Other specific care provision (wound care, trauma and surgical care, obstetric fistula care, and surgical care of typhoid related complications)</td><td>Programme-level routine data (MT)</td></tr>
</table>

| | | | | |
|---|---|---|---|---|
| | WHO region | Categorical (nominal) | The location of each mission was labelled according to the region codes used by the World Health Organisation:<br>- AFR<br>- EMR<br>- SEAR<br>- AMR<br>- WPR | WHO |
| | Setting | Categorical (binary) | 3 variables:<br>- Conflict<br>- Natural disaster<br>- Health care gaps | Programme-level routine data (MT) |
| | Hospital level | Ordinal (Categorical) | 4 distinct categories as per MSF-OCB surgical policy guidelines:<br>- Sole remit hospital = hospital that provides care for a specific purpose (i.e. not necessarily a quaternary referral hospital, but a surgical setup for a specific indication). Examples include: Trauma centre, Maternity centre, Fistula repair camp.<br>- Referral hospital = provincial hospital, considered tertiary referral hospital.<br>- District hospital = can manage most, but will refer complex cases on to referral hospitals.<br>- Health centres = small rural health centres with capacity to perform basic surgical operations | Programme-level routine data (MT) |
| | Site ID | Character | Anonymous unique code for each site | produced in R 3.6 |

For peer review only

**2. Table 2: surgical projects in health care gap settings in the WHO SEA region (2 in total)**

| | |
|---|---|
| **Type of anaesthesia provider** | Physician anaesthetist only |
| **Type of hospital** | - District hospital<br>- Health centre |
| **Type of care provided by surgical project (No. of projects)** | - Capacity to perform both emergency and elective surgery<br>- Filiariasis-related care |
| **Total duration, months** | 19 |
| **Type of surgical provider for entire duration** | - General & specialty surgeons<br>- General surgeon only |
| **Total number of all cases, No.** | 815 |
| **Main cause for hospitalisation, No. (%)** | - Other, 482 (59) |
| **Main category of surgery, No. (%)** | - Minor Surgery, 507 (62) |
| **Intra-operative mortality, No. (%)** | 2 (0.2) |

For peer review only

3. **Table 3: Surgical projects in natural disaster settings (5 in total)**

| | |
|---|---|
| **Type of anaesthesia provider** | Physician anaesthetist only |
| **Type of hospital** | - District<br>- Sole remit hospital |
| **Type of care provided by surgical project (No. of projects)** | - Capacity to perform both emergency and elective surgery (2)<br>- Trauma care only (1)<br>- Emergency only (2) |
| **Total duration, months** | 40 |
| **Presence of surgical provider, months** | |
| - **General and specialty surgeons** | 23 |
| - **General surgeon only** | 16 |
| - **Specialty surgeon only** | 1 |
| **Total number of all cases, No. (%)** | 3108 |
| **Main cause for hospitalisation, No. (%)** | - Other, 1144 (37) |
| **Main category of surgery, No. (%)** | - Minor surgery, 1608 (52) |
| **Intra-operative mortality, No. (%)** | 9 (0.3) |

For peer review only

**4. Table 4: Surgical groupings as used in main table 2 (case-level data)**

| Surgical grouping | Examples of types of surgery included |
|---|---|
| **Minor surgery** | • Simple wound treatment<br>• Insertion/removal of drain<br>• Burns dressing change<br>• Wound debridement<br>• Removal of foreign body<br>• Amputation of digits or toes<br>Incl. procedure codes with median operative time < 45min within the dataset:<br>• Curettage post delivery (GP)<br>• Reduction of fractures (OR)<br>• Removal of osteosynthesis (OX)<br>• Ophthalmic surgery (SO) |
| **Caesarean section** | • Caesarean section only |
| **Visceral surgery** | • Exploratory laparotomy<br>• Hernia repair<br>• Resection/repair solid organs (e.g. spleen/liver) or gut |
| **Orthopaedic surgery** | • External or internal fixation of fracture<br>• Surgery to any joint<br>• Limb amputation (excluding digits or toes)<br>• Curettage for osteomyelitis |
| **Obstetric & gynaecological surgery (excl. Caesarean section)** | • Management of ectopic pregnancy<br>• Obstetric fistula repair<br>• Hysterectomy |
| **Specialties** | • Urology<br>• Vascular surgery<br>• Plastic and reconstructive surgery<br>• Ear, nose and throat surgery<br>• Neurosurgery<br>• Thoracic surgery<br>• Maxillofacial surgery<br>• Other specialized surgery |

Modified from the original surgical groupings outlined in the “MSF-OCB Operating Department Data Collection Guidelines (2015)”.

For peer review only

### 5. Missing data

**Table 1: Number of missing values within each variable**

| Variable | Number missing |
|---|---|
| **ASA** | 3232 |
| **Intra-operative mortality** | 2154 |
| **Time in theatre** | 1922 |
| **Age** | 47 |
| **Main procedure** | 5 |
| **Choice of anaesthesia** | 2 |
| **Gender** | 0 |
| **Date of procedure** | 0 |
| **Cause of hospitalisation** | 0 |
| **Urgency** | 0 |
| **Order** | 0 |
| **Surgical provider** | 0 |
| **Anaesthesia provider** | 0 |
| **Who region** | 0 |
| **Setting** | 0 |
| **Type of hospital** | 0 |
| **Type of care provided** | 0 |
| **Site ID** | 0 |

For peer review only

Figure 1: Proportion of missing data in each surgical project

histogram showing all surgical projects (along x−axis), sorted according to setting,
showing proportion of missing cases with any missing data (red indicates data with at least one missing value)

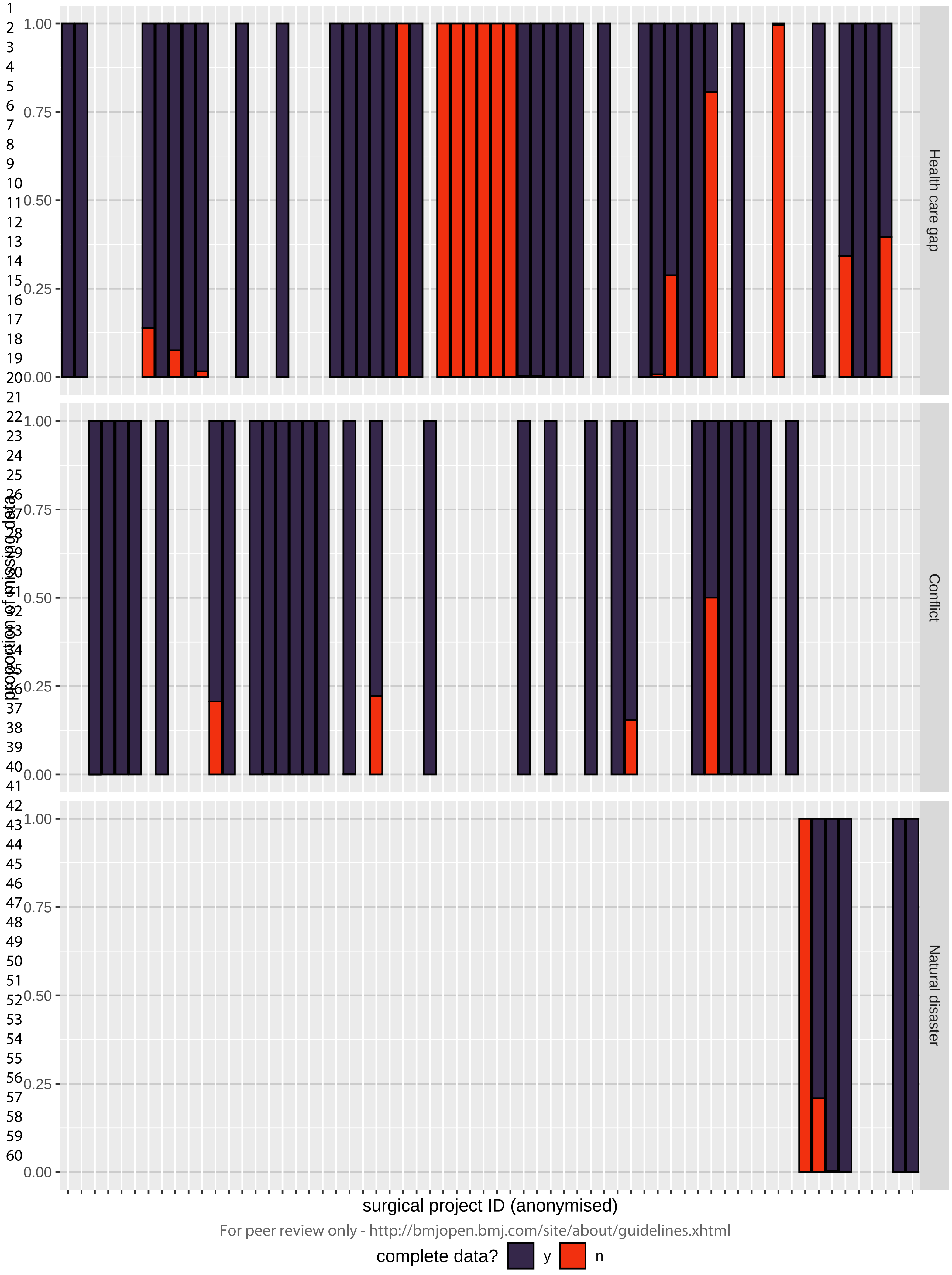

**Table 2: overview of available data**

| Variable | Value |
|---|---|
| **Median age, years (IQR)** | 26 (18-36) |
| **Female, no. (%)** | 2963 (52) |
| **Median ASA (IQR)** | 1 (1-2) |
| **Emergent surgery, no. (%)** | 2277 (40) |
| **Cause for hospitalisation, no. (%)** | |
| - **Obstetric** | 1188 |
| - **Any trauma** | 2074 |
| - **Other** | 2468 |
| **Main surgical procedure** | |
| - **Minor surgery** | 2854 |
| - **Caesarean section** | 746 |
| - **Visceral surgery** | 1408 |
| - **Obstetric and gynaecology** | 503 |
| - **Orthopaedics** | 177 |
| - **Other specialty surgery** | 37 |
| **Intraoperative mortality** | |
| - **Alive** | 3563 |
| - **Died** | 13 |
| **Median theatre time, minutes (IQR)** | 45 (35-60) |
| **Setting** | |
| - **Health care gap** | 3359 |
| - **Conflict** | 1443 |
| - **Natural disaster** | 928 |

For peer review only

**The use of different anaesthesia providers in humanitarian settings: Descriptive study of 173,084 episodes of surgical care provided by Médecins Sans Frontières over 10 years**

The RECORD statement – checklist of items, extended from the STROBE statement, that should be reported in observational studies using routinely collected health data.

| | Item No. | STROBE items | Location in manuscript where items are reported | RECORD items | Location in manuscript where items are reported |
|---|---|---|---|---|---|
| **Title and abstract** | | | | | |
| | 1 | (a) Indicate the study's design with a commonly used term in the title or the abstract<br><br>(b) Provide in the abstract an informative and balanced summary of what was done and what was found | Title<br>Page 1<br><br>Abstract<br>Page 4 | RECORD 1.1: The type of data used should be specified in the title or abstract. When possible, the name of the databases used should be included.<br><br>RECORD 1.2: If applicable, the geographic region and timeframe within which the study took place should be reported in the title or abstract.<br><br>RECORD 1.3: If linkage between databases was conducted for the study, this should be clearly stated in the title or abstract. | Abstract<br>Page 4<br><br>Abstract<br>Page 4<br><br>Abstract<br>Page 4 |
| **Introduction** | | | | | |
| Background rationale | 2 | Explain the scientific background and rationale for the investigation being reported | Introduction<br>Page 6 | | |
| Objectives | 3 | State specific objectives, including any prespecified hypotheses | Introduction<br>Page 6 | | |
| **Methods** | | | | | |
| Study Design | 4 | Present key elements of study design early in the paper | Methods<br>Page 6 | | |
| Setting | 5 | Describe the setting, locations, and relevant dates, including | Methods<br>Page 6-8 | | |

| | | | | | |
|---|---|---|---|---|---|
| | | periods of recruitment, exposure, follow-up, and data collection | | | |
| Participants | 6 | *(a) Cohort study* - Give the eligibility criteria, and the sources and methods of selection of participants. Describe methods of follow-up<br>*Case-control study* - Give the eligibility criteria, and the sources and methods of case ascertainment and control selection. Give the rationale for the choice of cases and controls<br>*Cross-sectional study* - Give the eligibility criteria, and the sources and methods of selection of participants<br><br>*(b) Cohort study* - For matched studies, give matching criteria and number of exposed and unexposed<br>*Case-control study* - For matched studies, give matching criteria and the number of controls per case | N/A<br>N/A<br>N/A<br>N/A<br>N/A | RECORD 6.1: The methods of study population selection (such as codes or algorithms used to identify subjects) should be listed in detail. If this is not possible, an explanation should be provided.<br><br>RECORD 6.2: Any validation studies of the codes or algorithms used to select the population should be referenced. If validation was conducted for this study and not published elsewhere, detailed methods and results should be provided.<br><br>RECORD 6.3: If the study involved linkage of databases, consider use of a flow diagram or other graphical display to demonstrate the data linkage process, including the number of individuals with linked data at each stage. | Methods Page 7<br><br>N/A<br><br>Inclusion flow diagram |
| Variables | 7 | Clearly define all outcomes, exposures, predictors, potential confounders, and effect modifiers. Give diagnostic criteria, if applicable. | Appendix Table 1 | RECORD 7.1: A complete list of codes and algorithms used to classify exposures, outcomes, confounders, and effect modifiers should be provided. If these cannot be reported, an explanation should be provided. | Appendix Table 1 |
| Data sources/ measurement | 8 | For each variable of interest, give sources of data and details of methods of assessment (measurement). | Appendix Table 1 | | |

For peer review only

| | | | | | |
|---|---|---|---|---|---|
| | | Describe comparability of assessment methods if there is more than one group | N/A | | |
| Bias | 9 | Describe any efforts to address potential sources of bias | Methods – bias<br>Page 7 | | |
| Study size | 10 | Explain how the study size was arrived at | Descriptive study – not performed | | |
| Quantitative variables | 11 | Explain how quantitative variables were handled in the analyses. If applicable, describe which groupings were chosen, and why | Appendix<br>Table 1 | | |
| Statistical methods | 12 | (a) Describe all statistical methods, including those used to control for confounding<br>(b) Describe any methods used to examine subgroups and interactions<br>(c) Explain how missing data were addressed<br>(d) *Cohort study* - If applicable, explain how loss to follow-up was addressed<br>*Case-control study* - If applicable, explain how matching of cases and controls was addressed<br>*Cross-sectional study* - If applicable, describe analytical methods taking account of sampling strategy<br>(e) Describe any sensitivity analyses | Descriptive study – not performed<br><br>Methods – bias<br>Page 7<br><br>Results (page 15) appendix (5. Missing data, table 1 & 2, Figure 1)<br><br>n/a<br><br>n/a<br><br>n/a<br><br>n/a | | |
| Data access and cleaning methods | | .. | | RECORD 12.1: Authors should describe the extent to which the investigators had access to the database | Methods<br>Page 7 |

For peer review only

| | | | | | |
|---|---|---|---|---|---|
| | | | | population used to create the study population.<br><br>RECORD 12.2: Authors should provide information on the data cleaning methods used in the study. | <br><br><br>Methods Page 6 |
| Linkage | | .. | | RECORD 12.3: State whether the study included person-level, institutional-level, or other data linkage across two or more databases. The methods of linkage and methods of linkage quality evaluation should be provided. | Methods (page 6) & inclusion flow diagram |
| **Results** | | | | | |
| Participants | 13 | (a) Report the numbers of individuals at each stage of the study (*e.g.*, numbers potentially eligible, examined for eligibility, confirmed eligible, included in the study, completing follow-up, and analysed)<br>(b) Give reasons for non-participation at each stage.<br>(c) Consider use of a flow diagram | inclusion/exclusion diagram<br><br><br><br><br><br><br>N/A<br><br>inclusion/exclusion diagram | RECORD 13.1: Describe in detail the selection of the persons included in the study (*i.e.,* study population selection) including filtering based on data quality, data availability and linkage. The selection of included persons can be described in the text and/or by means of the study flow diagram. | Methods (page 7) & inclusion/exclusio n diagram |
| Descriptive data | 14 | (a) Give characteristics of study participants (*e.g.*, demographic, clinical, social) and information on exposures and potential confounders<br>(b) Indicate the number of participants with missing data for each variable of interest<br>(c) *Cohort study* - summarise follow-up time (*e.g.*, average and total amount) | Results – table 1<br><br><br><br><br>Appendix (5. Missing data, table 1)<br><br><br>N/A | | |

For peer review only

| | | | | | |
|---|---|---|---|---|---|
| Outcome data | 15 | *Cohort study* - Report numbers of outcome events or summary measures over time<br>*Case-control study* - Report numbers in each exposure category, or summary measures of exposure<br>*Cross-sectional study* - Report numbers of outcome events or summary measures | N/A<br><br>N/A<br><br>Results<br>Page 8 | | |
| Main results | 16 | (a) Give unadjusted estimates and, if applicable, confounder-adjusted estimates and their precision (e.g., 95% confidence interval). Make clear which confounders were adjusted for and why they were included<br>(b) Report category boundaries when continuous variables were categorized<br>(c) If relevant, consider translating estimates of relative risk into absolute risk for a meaningful time period | Results<br>Page 8 onwards + table 1 (page 10) and 2 (page 13)<br><br>Results<br>table 1 (page 10) and 2 (page 13)<br><br>N/A | | |
| Other analyses | 17 | Report other analyses done—e.g., analyses of subgroups and interactions, and sensitivity analyses | N/A | | |
| **Discussion** | | | | | |
| Key results | 18 | Summarise key results with reference to study objectives | Discussion<br>Page 16 | | |
| Limitations | 19 | Discuss limitations of the study, taking into account sources of potential bias or imprecision. Discuss both direction and magnitude of any potential bias | Discussion<br>Page 16 | RECORD 19.1: Discuss the implications of using data that were not created or collected to answer the specific research question(s). Include discussion of misclassification bias, unmeasured confounding, missing | Discussion<br>Page 16 |

For peer review only

| | | | | | |
|---|---|---|---|---|---|
| | | | | data, and changing eligibility over time, as they pertain to the study being reported. | |
| Interpretation | 20 | Give a cautious overall interpretation of results considering objectives, limitations, multiplicity of analyses, results from similar studies, and other relevant evidence | Discussion Page 16 | | |
| Generalisability | 21 | Discuss the generalisability (external validity) of the study results | Discussion Page 16 | | |
| **Other Information** | | | | | |
| Funding | 22 | Give the source of funding and the role of the funders for the present study and, if applicable, for the original study on which the present article is based | Page 17 | | |
| Accessibility of protocol, raw data, and programming code | | .. | | RECORD 22.1: Authors should provide information on how to access any supplemental information such as the study protocol, raw data, or programming code. | Page 17 |

*Reference: Benchimol EI, Smeeth L, Guttmann A, Harron K, Moher D, Petersen I, Sørensen HT, von Elm E, Langan SM, the RECORD Working Committee. The REporting of studies Conducted using Observational Routinely-collected health Data (RECORD) Statement. *PLoS Medicine* 2015; in press.

*Checklist is protected under Creative Commons Attribution (CC BY) license.

For peer review only

BMJ Open

# Anaesthesia care providers employed in humanitarian settings by Médecins Sans Frontières: A retrospective observational study of 173,084 surgical cases over 10 years

| | |
|---|---|
| Journal: | *BMJ Open* |
| Manuscript ID | bmjopen-2019-034891.R2 |
| Article Type: | Original research |
| Date Submitted by the Author: | 15-Jan-2020 |
| Complete List of Authors: | Kudsk-Iversen, Soren; Oxford University Hospitals NHS Foundation Trust, Nuffield Department of Anaesthetics<br>Trelles, Miguel; Medecins Sans Frontieres<br>Ngowa Bakebaanitsa, Elie; Medecins Sans Frontieres; Masisi referral hospital, Masisi – MSF Democratic Republic of the Congo mission<br>Hagabimana, Longin; Medecins Sans Frontieres; Arche trauma hospital, Bujumbura – MSF Burundi mission<br>Momen, Abdul; Medecins Sans Frontieres; Khost maternity, Khost – MSF Afghanistan mission<br>Helmand, Rahmatullah; Medecins Sans Frontieres; Ahmad Shah Baba hospital, Kabul – MSF Afghanistan mission<br>Saint Victor, Carline; Medecins Sans Frontieres; Tabarre trauma hospital, Port-au-Prince – MSF Haiti mission<br>Shah, Khalid; Medecins Sans Frontieres; Timurgara district headquarter hospital – MSF Pakistan mission<br>Masu, Adolphe; Medecins Sans Frontieres; Castors maternity, Bangui – MSF Central African Republic mission<br>Kendell, Judith; Medecins Sans Frontieres<br>Edgcombe, Hilary; Oxford University Hospitals NHS Foundation Trust, Nuffield Department of Anaesthetics<br>English, Mike; KEMRI - Wellcome Trust Research Programme, Health Services Unit; University of Oxford Centre for Tropical Medicine and Global Health |
| <b>Primary Subject Heading</b>: | Anaesthesia |
| Secondary Subject Heading: | Global health |
| Keywords: | ANAESTHETICS, Human resource management < HEALTH SERVICES ADMINISTRATION & MANAGEMENT, Organisation of health services < HEALTH SERVICES ADMINISTRATION & MANAGEMENT |
| | |

SCHOLARONE™
Manuscripts

*I, the Submitting Author has the right to grant and does grant on behalf of all authors of the Work (as defined in the below author licence), an exclusive licence and/or a non-exclusive licence for contributions from authors who are: i) UK Crown employees; ii) where BMJ has agreed a CC-BY licence shall apply, and/or iii) in accordance with the terms applicable for US Federal Government officers or employees acting as part of their official duties; on a worldwide, perpetual, irrevocable, royalty-free basis to BMJ Publishing Group Ltd ("BMJ") its licensees and where the relevant Journal is co-owned by BMJ to the co-owners of the Journal, to publish the Work in this journal and any other BMJ products and to exploit all rights, as set out in our licence.*

*The Submitting Author accepts and understands that any supply made under these terms is made by BMJ to the Submitting Author unless you are acting as an employee on behalf of your employer or a postgraduate student of an affiliated institution which is paying any applicable article publishing charge ("APC") for Open Access articles. Where the Submitting Author wishes to make the Work available on an Open Access basis (and intends to pay the relevant APC), the terms of reuse of such Open Access shall be governed by a Creative Commons licence – details of these licences and which Creative Commons licence will apply to this Work are set out in our licence referred to above.*

*Other than as permitted in any relevant BMJ Author's Self Archiving Policies, I confirm this Work has not been accepted for publication elsewhere, is not being considered for publication elsewhere and does not duplicate material already published. I confirm all authors consent to publication of this Work and authorise the granting of this licence.*

For peer review only

# Anaesthesia care providers employed in humanitarian settings by Médecins Sans Frontières: A retrospective observational study of 173,084 surgical cases over 10 years

Søren Kudsk-Iversen, MBChB DTM&H FRCA
Address:
Stoke Mandeville Hospital, Mandeville Rd, Aylesbury HP21 8AL, UK

Affiliation:
(1) Nuffield Department of Anaesthetics, Oxford University Hospitals NHS Trust, Oxford, UK

*Corresponding author: sorenki@gmail.com, +44(0)7707838060

Miguel Trelles, PhD
Address:
Rue de l'Arbre Benit 46, 1050 Bruxelles, Belgique

Affiliation:
(1) Médecins sans Frontières
(2) Medical Department – Operational Centre Brussels

Elie Ngowa Bakebaanitsa, Diplôme de Anesthésie et Réanimation
Address:
l'Hôpital général de reference de masisi, zone de santé de masisi, division provinciale de santé du nord-kivu, Republique Democratique du Congo

Affiliation:
(1) Médecins sans Frontières
(2) Masisi referral hospital, Masisi – MSF Democratic Republic of the Congo mission

Longin Hagabimana, Diplôme de Licence en Anesthésie-Réanimation
Address:
L'Hôpital Arche Médical de Kigobe, Bujumbura, Burundi

Affiliation:
(1) Médecins sans Frontières
(2) Arche trauma hospital, Bujumbura – MSF Burundi mission

Abdul Momen, MD
Address:
Khost Maternity Hospital, Khost, Afghanistan

Affiliation:
(1) Médecins sans Frontières
(2) Khost maternity, Khost – MSF Afghanistan mission


For peer review only - http://bmjopen.bmj.com/site/about/guidelines.xhtml

Rahmatullah Helmand, MD

Address:

Jamhoriat Hospital, Chackin Street, New City, Kabul, Afganistan

Affiliation:

(1) Médecins sans Frontières
(2) Ahmad Shah Baba hospital, Kabul – MSF Afghanistan mission

Carline Saint Victor, Certificat en soins anesthésiques et réanimation

Address:

Maternité Isai Jeanty (Hopital Chancerelles), Boulevard Jean-Jacques Dessalines, Port-au-Prince, Haiti

Affiliation:

(1) Médecins sans Frontières
(2) Tabarre trauma hospital, Port-au-Prince – MSF Haiti mission

Khalid Shah, Post RN Dip (Anesthesia)

Address:

DHQ Hospital Timergara, Lower Dir, Khyber Pakhtunkhwa, Pakistan

Affiliations:

(1) Médecins sans Frontières
(2) Timurgara district headquarter hospital – MSF Pakistan mission

Adolphe Masu, MMed

Address:

Avenue Tilipiers number 30, Quartier les Volcans, Ville de Goma, North Kivu province, Republique Democratique du Congo

Affiliations:

(1) Médecins sans Frontières
(2) Arche trauma hospital, Bujumbura – MSF Burundi mission
(3) Khost maternity, Khost – MSF Afghanistan mission
(4) Castors maternity, Bangui – MSF Central African Republic mission

Judith Kendell, MBBS

Address:

Médecins sans Frontières, Rue de l’Arbre Bénit 46, 1050 Bruxelles, Belgique

Affiliation:

(1) Médecins sans Frontières
(2) Medical Department – Operational Centre Brussels

For peer review only

Hilary Edgcombe, BM BCh FRCA
Address:
Nuffield Department of Anaesthetics, Level 2, John Radcliffe Hospital, Headley Way OX3 9DU

Affiliation:
(1) Nuffield Department of Anaesthetics, Oxford University Hospitals NHS Trust, Oxford, UK

Mike English, FMedSci
Address:
Centre for Tropical Medicine and Global Health, University of Oxford, Old Road Campus, Roosevelt Drive, Oxford OX3 7FZ, UK

Affiliation:
(1) KEMRI-Wellcome Trust Research Programme, Nairobi, Kenya
(2) Centre for Tropical Medicine and Global Health, University of Oxford, Oxford, UK

**Key words:**

Global health
Humanitarian aid
Anaesthesia
Task-shifting and sharing

Word Count (excluding title page, abstract, references, tables, figures): 3371 words

For peer review only

**Abstract**

**Objective:**

To describe the extent to which different categories of anaesthesia provider are used in humanitarian surgical projects and to explore the volume and nature of their surgical workload.

**Design:**

Descriptive analysis using 10 years (2008-2017) of routine case-level data linked with routine program-level data from surgical projects run exclusively by MSF-Operational Centre Brussels (MSF-OCB).

**Setting:**

Projects were in contexts of natural disaster (ND, entire expatriate team deployed by MSF-OCB), active conflict (AC), and stable health care gaps (HG). In AC and HG settings MSF-OCB support pre-existing local facilities. Hospital facilities ranged from basic health centres with surgical capabilities to tertiary referral centres.

**Participants:**

The full dataset included 178,814 surgical cases. These were categorised by most senior anaesthetic provider for the project, according to qualification: Specialist physician anaesthesiologists, qualified nurse anaesthetists, and uncertified anaesthesia providers.

**Primary Outcome Measure:**

Volume and nature of surgical workload of different anaesthesia providers.

**Results:**

Full routine data were available for 173,084 cases (96.8%): 2,518 in ND, 42,225 in AC, 126,936 in HG. Anaesthesia was predominantly led by physician anaesthesiologists (100% in ND, 66% in AC and HG), then nurse anaesthetists (19% in AC and HG) or uncertified anaesthesia providers (15% in AC and HG). Across all settings and provider groups, patients were mostly healthy young adults (median age range 24-27 years), with predominantly females in HG contexts, and males in AC contexts. Overall intra-operative mortality was 0.2%.

**Conclusion**:

Our findings contribute to existing knowledge of the nature of anaesthetic provision in humanitarian settings, whilst demonstrating the value of high quality, routine data collection at scale in this sector. Further evaluation of perioperative outcomes associated with different models of humanitarian anaesthetic provision is required.




For peer review only - http://bmjopen.bmj.com/site/about/guidelines.xhtml

**Article Summary**

**Strengths and limitations of this study**

- This is the largest study detailing how anaesthetic task sharing and shifting is employed in the humanitarian sector

- Additionally, we believe this is the first study to describe the extent of the presence and caseload of uncertified anaesthetic providers in humanitarian surgical projects

- Due to the nature of the linked data, we were unable to connect anaesthetic provider with individual operations. Therefore, to limit the misclassification bias, we do not ascribe a provider to each case, but rather describe the most senior provider available in the surgical project (the 'anaesthetic lead')

For peer review only

## INTRODUCTION

Globally there is a large unmet surgical need. Low and middle-income countries (LMIC) are disproportionately affected by gaps in health care provision, with an estimated 90% of patients in these countries unable to access basic surgical care.[1] The burden is increased and access further reduced in crisis situations, caused by conflict or natural disasters.[2] To address these imbalances, Médecins Sans Frontières (MSF, also known as Doctors without Borders) provide humanitarian surgical assistance based on the needs of affected populations through one or more of their five operational centres, one of which is Operational Centre Brussels (MSF-OCB).

There is an increasing body of literature outlining the surgical needs of populations in humanitarian settings.[3–6] The recognition that the humanitarian sector is not immune from the need to demonstrate safe surgical care has led to calls for more robust outcome data and clearer accountability.[7–9] Only few studies, limited by small study size and limited external validity, have addressed the composition of the surgical workforce employed by humanitarian organisations.[10,11] Therefore, there is inadequate published data on whether different anaesthesia providers (e.g. physician, nurse, or other health care provider) are employed in different settings, and to what extent there is a physician expatriate presence within the team. In order to comment on outcomes and identify areas where practice can be improved, it is essential to know who provides the care and if there is any learning that can be derived from their practice.

The objective of this study is to describe the extent to which different categories of anaesthesia provider are used in humanitarian surgical projects and to explore the volume and nature of their surgical workload.

## METHODS

The study protocol was submitted to the Oxford Tropical Research Ethics Committee who granted ethical exemption. The study also fulfilled the exemption criteria set by the MSF Ethics Review Board for *a posteriori* analyses of routinely collected clinical data and thus did not require MSF ERB review. It was conducted with permission from Medical Director, MSF-OCB. This exemption did not allow country/site specific detail to be included, therefore we aggregate data within the World Health Organisation (WHO) regional groupings.[12] The findings are reported in accordance with RECORD, the extended STROBE statement on routinely collected data.[13]

### Study design

This was a descriptive study of routine data collected between January 2008 and December 2017. We excluded any incomplete data and data from surgical projects where MSF-OCB were collaborating with other MSF operational centres or local governments, as we were unable to account for workforce or resources made available by others than MSF-OCB.

We linked three sources of data (see figure 1). 1) Case-level routine surgical surveillance data were recorded by theatre staff in logbooks on-site, then transcribed onto an Excel spreadsheet, and finally transferred to Brussels on a monthly basis where they were reviewed and any missing or extraneous data was queried with the local teams. 2) Program-level data, available from MT (head of the Surgical, Anaesthesia, Gynaecology, and Emergency Medicine unit during this period) were reviewed. 3) End of deployment reports written by expatriate physician anaesthesiologists were reviewed to fill gaps in data from the case-level


For peer review only - http://bmjopen.bmj.com/site/about/guidelines.xhtml

data. Data were de-identified at point of data collection, and were only accessed by SK, MT and JK. Any data shared with the remaining co-authors were fully anonymized.

**Setting and anaesthesia providers**

Three different project setting types were identified: 1) regions recently affected by sudden onset natural disasters (ND) where MSF deployed an entire expatriate surgical team in accordance with WHO minimum requirements,[14] 2) active armed conflict (AC) situations and 3) stable situations where MSF supported a pre-existing local facility to address health-care gaps (HG), which existed for a variety of reasons, including the aftermath of natural disasters or armed conflict.

The setup and duration of surgical projects varied. Some projects were intended to operate only for a short period, either within existing local infrastructure or through fully self-contained surgical platforms. Other projects were set up to serve for a longer period or evolved over time into a fully functioning hospital with ability to provide complex care provision. The different hospital types are described in detail in the appendix (appendix, table 1). The setup was not dictated by the setting, and could change over the course of a project. During the 10 years studied, anaesthesia provision was led by one of the following: a) specialist physician anaesthesiologists, either local or expatriate (from both high and low income settings) doctors with qualifications in anaesthesia, b) nurse anaesthetists, either local or expatriate (predominantly from low income settings) nurses or other non-physician clinical cadres with formal training and qualification in anaesthesia in their country of origin, or c) uncertified anaesthesia providers, local nurses or allied health care professionals with a broad range of different levels of experience in anaesthesia provision but without a formal qualification who received on-the-job training only. The MSF-OCB anaesthesia referent assesses the provider requirement for each location based on expected workload, job description and staff availability. For example, if a project is expected to have a low workload, nurse anaesthetists are either recruited locally or, if they are senior providers, sent over as expatriates from MSF-OCB surgical projects in other countries. In situations where MSF-OCB are unable to source qualified staff for a surgical project, they may hire the existing local uncertified anaesthesia providers, who will all receive on-the-job training by MSF and supervision by expatriate physician anaesthesiologists for a trial period. These situations should result in uncertified anaesthesia providers working in settings with a low workload and with distant supervision available from a nearby hospital with MSF-OCB involvement where anaesthesia is led by an expatriate physician anaesthesiologist. All MSF surgical projects have standardised anaesthetic equipment and medications, as described elsewhere.[5]

**Variables and bias**

Different variables were retrieved from the three different data sources. From the routine case-level data (and end of deployment reports) we identified patient variables (including age and sex), surgical and anaesthetic variables (including type of surgery, type of anaesthesia), and geographic location of the cases done. From the program-level data we obtained additional surgical and anaesthetic variables (including provider level of training, presence of expatriate), and location variables (including project setting, type of hospital). A detailed description of all variables used is available in the appendix, table 1.



For peer review only - http://bmjopen.bmj.com/site/about/guidelines.xhtml

The use of routine surveillance data puts the study at risk of selection bias, which may risk under-reporting by some providers (e.g. expatriates visiting for short periods who may be unfamiliar with the data collection tool, or staff who for whatever reason choose not to document cases) or in busy settings (e.g. high workload or strained workforce). While we cannot account for surgical cases not recorded in the first place, we explored incomplete data that had been excluded to assess similarity to the included data.
Furthermore, it should be noted that provider data were available showing the most senior provider present for each project, not per case (and for expatriates, was updated monthly during a project). This puts the study at risk of misclassification bias regarding the anaesthesia providers in favour of the most senior team member regardless of their presence in theatre. Additionally, it would be easy to overrepresent the case-level involvement of physician anaesthesiologists (especially when they are present as expatriates, as they might be more restricted in their movement and have additional non-clinical commitments). We therefore present data according to the most senior provider present on the project in a given month (the anaesthetic 'lead'). We also note which projects had a visiting expatriate physician anaesthesiologist present.

**Statistical analysis**
Data were collected and linked in Excel (2016) and data cleaning and analysis was performed in R 3.6. Continuous data were assessed for normality, and no parametric data were identified. For non-parametric continuous and numeric ordinal data, median, interquartile (IQR) and full range were reported. For categorical variables, the raw counts were reported. We stratified our analysis according to the settings identified, as they might influence the extent and pattern by which different anaesthetic providers were deployed.
However, data from surgical projects in the WHO South East Asia region and in ND settings were described separately due to their small numbers and being separate from the dominant regions (see appendix, tables 2 and 3).

**Patient and public involvement**
There was no involvement of patients or the public in the development or execution of this study.

## RESULTS

**General findings**
Over the 10 years a total of 173,084 cases had full routine data collected (96.8% of all cases) across 23 countries and 52 different locations (see figure 1). The majority of cases occurred in HG settings, and in the WHO Africa region (see figure 2). Surgical projects in settings of ND represented 3,108 cases (less than 2% of the total number of operations over the time period) and a total duration of 40 project-months over 5 sites; anaesthesia care in the ND setting was exclusively led by physician anaesthesiologists (see appendix, table 3).

Overall the shortest surgical project lasted a month, and the longest lasted beyond the 10 years covered by this study (see figure 3). Surgical projects in HG settings stayed open for longer (median 866 days, IQR 360.25-1900 days) than projects in AC and ND settings (287.5, 173-498.25 days and 210, 122-308 days, respectively). The workload within each


For peer review only - http://bmjopen.bmj.com/site/about/guidelines.xhtml

project varied widely, with 31 projects accounting for 5.1% of all cases, and four projects accounting for 47.6% (see figure 3A).

Of the four biggest projects, anaesthesia for two projects was exclusively physician anaesthesiologist-led (one in the WHO Eastern Mediterranean region in an AC setting, the other in the WHO Americas region in a HG setting). The third project was predominantly physician anaesthesiologist-led (in the WHO Eastern Mediterranean region) progressing from an initial AC to become a stable HG setting. The last was predominantly uncertified anaesthesia provider-led with a periodic presence of expatriate physician anaesthesiologists (in the WHO Africa region, starting in AC and then becoming a stable HG setting). Data for these four major projects followed a similar pattern of distribution (in terms of case and program-level data) to the remaining dataset of all other projects, and have therefore been included in the findings below.

**Program-level provider findings**

Most surgical projects (23/28 in AC, 25/32 in HG, and all 5 in ND) included a period of anaesthesia provision led by physician anaesthesiologists (see figure 3B and table 1A). Anaesthesia in any setting with sole trauma care was mostly led by physician anaesthesiologists (see table 1A). If anaesthesia provision in a project was not fully physician anaesthesiologist-led, the pattern of their presence in most cases involved short periods (usually around 3 months) over the course of the surgical project, mostly towards the start of the project (see figure 3B). Overall, a physician anaesthesiologist was identified as present for 737 (49%) project-months in AC and HG (see table 1B). However, in these settings more than 66% of cases overall were conducted during periods where physician anaesthesiologist were present in the projects (80% of cases in AC and 60% of cases in HG).

When there was not a physician anaesthesiologist attached to a project, anaesthesia was most commonly led by nurse anaesthetists in the HG setting and most commonly led by uncertified anaesthesia providers in the AC setting.

For peer review only



For peer review only - http://bmjopen.bmj.com/site/about/guidelines.xhtml

**Table 1A: Programme-level descriptive table according to number of surgical projects in different settings**

| | Physician anaesthesiologist led | Nurse anaesthetist led | Uncertified anaesthetic provider led | Physician anaesthesiologist led | Nurse anaesthetist led | Uncertified anaesthetic provider led |
|---|---|---|---|---|---|---|
| | **CONFLICT (SURGICAL PROJECTS = 28)** | | | **HEALTH CARE GAP (SURGICAL PROJECTS = 32)[a]** | | |
| **Number of surgical projects involved in at any point[b]** | 23 | 14 | 12 | 25 | 17 | 12 |
| **Type of hospital[c] in surgical project, No. surgical projects involved in at any point[d]** | | | | | | |
| **Sole remit hospital** | 5 | 2 | 1 | 13 | 7 | 1 |
| **Referral hospital** | 6 | 5 | 2 | 7 | 5 | 2 |
| **District hospital** | 11 | 4 | 8 | 7 | 4 | 7 |
| **Health centre** | 1 | 3 | 1 | 0 | 1 | 2 |
| **Type of surgical care performed in project, No. surgical projects involved in at any point[d]** | | | | | | |
| **Emergency only** | 9 | 8 | 5 | 3 | 4 | 4 |
| **Capacity to perform both emergency and elective surgery** | 8 | 4 | 6 | 11 | 5 | 6 |
| **Maternity care only** | 1 | 1 | 1 | 9 | 6 | 1 |
| **Trauma care only** | 4 | 1 | 0 | 2 | 0 | 0 |


For peer review only - http://bmjopen.bmj.com/site/about/guidelines.xhtml

| | | | | | | | | |
|---|---|---|---|---|---|---|---|---|
| **Other specific care provision[e]** | 1[1] | 0 | 0 | | 2[2,3] | 1[3] | 1[4] | |

**Table 1B: Programme-level descriptive table according to months of activity in different settings**

| | Physician anaesthesiologist led | Nurse anaesthetist led | Uncertified anaesthetic provider led | All conflict missions | Physician anaesthesiologist led | Nurse anaesthetist led | Uncertified anaesthetic provider led | All health care gap missions |
|---|---|---|---|---|---|---|---|---|
| | **CONFLICT (SURGICAL PROJECTS = 28)** | | | | **HEALTH CARE GAP (SURGICAL PROJECTS = 32)[a]** | | | |
| **Number of months active in any mission** | 235 | 75 | 94 | **404** | 502 | 429 | 160 | **1091** |
| **Surgical provider, No. months present (% of cohort)** | | | | | | | | |
| **General and specialty surgeon** | 100 (43) | 13 (17) | 0 (0) | **113 (28)** | 260 (52) | 105 (24) | 3 (2) | **368 (34)** |
| **General surgeon only** | 96 (41) | 58 (77) | 20 (20) | **174 (43)** | 115 (23) | 133 (31) | 38 (24) | **286 (26)** |
| **Specialty surgeon only** | 31 (13) | 2 (3) | 4 (4) | **37 (9)** | 103 (21) | 162 (38) | 7 (4) | **272 (25)** |
| **MD[f]** | 8 (3) | 2 (3) | 70 (74) | **80 (20)** | 24 (5) | 29 (7) | 112 (70) | **165 (15)** |

[a] South East Asia Region contributed such a small proportion to missions in "health care gap" settings (2 missions, 815 cases or <1%), that they have been excluded and instead described in appendix, table 2.

[b] Surgical projects can have anaesthesia provision by multiple different providers during the period they are open. Therefore, the rows might add up to more than the total number of projects in each setting.

[c] Definitions of hospitals found in appendix, table 1.

[d] Two surgical projects changed from being able to provide both emergency and elective surgery, to providing solely maternity care. As such, they are counted twice under "type of hospital" and "type of surgical care".

[e] Specific care provision are surgical projects with a specific care remit. This includes [1]wound care, [2]trauma and surgical care, [3]obstetric fistula care, and [4]surgical care of typhoid related complications.

[f] MD = local physician with surgical skills but without a formal surgical qualification.



For peer review only - http://bmjopen.bmj.com/site/about/guidelines.xhtml

**Case-level provider findings**

Case-mix was similar across all lead providers with respect to age (mostly young adults) and underlying health (mostly ASA 1) (see table 2). All providers did predominantly non-elective work with trauma surgery more commonly done in physician led projects in both AC and HG and caesarean sections more commonly done in nurse anaesthesia projects, especially in HG settings. The intra-operative mortality was 0.3% and 0.3% in physician anaesthesiologist-led project-months, 0.2% and 0.1% in nurse anaesthetist-led project-months, and 0.3% and 0.2% in uncertified anaesthesia provider-led project-months in AC and HG settings, respectively. All lead providers made use of the two most common types of anaesthesia: spinal injection alone and general anaesthesia (GA) without intubation or muscle relaxant, which for the most part was ketamine-based. This was done in broadly similar proportions when comparing surgical categories in different settings (as an example, spinal injection and GA without protected airway for caesarean section was 61-70% and 22-36%, respectively in AC, and 78-86% and 6-14%, respectively in HG).

For peer review only


For peer review only - http://bmjopen.bmj.com/site/about/guidelines.xhtml

**Table 2: Case-level descriptive table grouped according to setting[a]**

| | Physician anaesthesiologist led | Nurse anaesthetist led | Uncertified anaesthetic provider led | Physician anaesthesiologist led | Nurse anaesthetist led | Uncertified anaesthetic provider led |
|---|---|---|---|---|---|---|
| | CONFLICT (SURGICAL PROJECTS = 28, N = 42,225) | | | HEALTH CARE GAP (SURGICAL PROJECTS = 32, N = 126,936)[b] | | |
| **Number of all surgical episodes, No.** | 33763 | 3798 | 4664 | 78126 | 28559 | 20251 |
| **Patient demographics** | | | | | | |
| **Female, No. (%)** | 12424 (37) | 1888 (50) | 2237 (48) | 38919 (50) | 22439 (79) | 12834 (63) |
| **Median age, years (IQR, [range])** | 23 (15-33, [1 day old-105]) | 25 (16-34, [2 day old-90]) | 23 (18-30, [3 day old-94]) | 28 (19-37, [1 day old-102]) | 26 (20-34, [1 day old-98]) | 25 (16-35, [1 day old-96]) |
| **ASA, value (IQR, [range])** | 1 (1-2, [1-5]) | 2 (1-2, [1-5]) | 1 (1-2, [1-5]) | 1 (1-2, [1-5]) | 1 (1-2, [1-5]) | 1 (1-2, [1-5]) |
| **Cause of hospitalisation, No. %:** | | | | | | |
| • **Trauma (intentional or unintentional)** | 21968 (65) | 1384 (36) | 2366 (51) | 42454 (54) | 2850 (10) | 6303 (31) |
| • **Obstetric** | 6642 (20) | 1073 (28) | 1295 (28) | 20387 (26) | 18270 (64) | 7211 (36) |
| • **Other[c]** | 5153 (15) | 1341 (35) | 1003 (22) | 15285 (20) | 7439 (26) | 6737 (33) |
| **Surgical demographics** | | | | | | |
| **Urgency, No. (%)** | | | | | | |
| • **Emergent** | 14344 (42) | 2203 (58) | 2581 (55) | 37234 (48) | 19922 (70) | 9221 (46) |
| • **Urgent** | 18091 (54) | 1115 (29) | 1961 (42) | 34771 (45) | 4781 (17) | 8699 (43) |
| • **Elective** | 1328 (4) | 480 (13) | 122 (3) | 6121 (8) | 3856 (14) | 2331 (12) |
| **Proportion of cases from initial presentation, n (%)** | 20079 (59) | 3053 (80) | 3588 (77) | 51493 (66) | 25597 (90) | 14492 (72) |
| **Median time in theatre, minutes (IQR, [range])** | 50 (30-70, [7-710]) | 50 (35-70, [15-356]) | 45 (35-65, [10-360]) | 60 (35-90, [10-870]) | 60 (50-80, [10-1140]) | 50 (30-70, [5-460]) |
| **Main categories of surgery, No. (%)[d]** | | | | | | |
| **Minor surgery** | 20670 (61) | 1688 (44) | 3019 (65) | 37419 (48) | 6798 (24) | 9906 (49) |


For peer review only - http://bmjopen.bmj.com/site/about/guidelines.xhtml

| | | | | | | |
|---|---|---|---|---|---|---|
| **Caesarean section** | 4758 (14) | 891 (23) | 884 (19) | 16138 (21) | 13336 (47) | 6259 (31) |
| **Visceral surgery** | 3709 (11) | 949 (25) | 555 (12) | 11109 (14) | 4856 (17) | 2922 (14) |
| **Orthopaedic surgery** | 3372 (10) | 90 (2) | 48 (1) | 9408 (12) | 151 (1) | 328 (2) |
| **Obstetric & gynaecological surgery (excl. caesarean section)** | 802 (2) | 122 (3) | 139 (3) | 3226 (4) | 3147 (11) | 756 (4) |
| **Specialties[e]** | 452 (1) | 58 (2) | 19 (0) | 826 (1) | 271 (1) | 80 (0) |
| **Intra-operative mortality, No. (%)** | | | | | | |
| **For all cases** | 102 (0·3) | 7 (0·2) | 16 (0·3) | 204 (0·3) | 31 (0·1) | 31 (0·2) |

[a] Percentages have been rounded to nearest full digit, and might not add up to 100%.

[b] South East Asia Region contributed such a small proportion to missions in “health care gap” settings (2 missions, 815 cases or <1%), that they have been excluded and instead described in the appendix, table 2.

[c] “Other” causes of hospitalisation include: tropical disease related, tumours, non-tumour related obstruction, and complications from traditional medical practices.

[d] The surgical procedures included in each grouping can be found in the appendix, table 4.

[e] Specialties encompass (total number of cases across whole dataset): Urology (726), vascular surgery (355), plastic and reconstructive surgery (144), ENT surgery (116), neurosurgery (115), surgery within thoracic cavity (108), maxillofacial surgery (61), and other forms of specialised surgical care that does not fall into the aforementioned categories (109).



For peer review only - http://bmjopen.bmj.com/site/about/guidelines.xhtml

**Missing data**

The cases excluded due to missing variables (5730, 3.2%) are predominantly from the early years. The three most common variables with missing data was ASA score (3232 missing), intra-operative mortality (2154), and time in theatre (1922) (see appendix, missing data table 1). The data with missing intra-operative mortality was exclusively from 2008, and were predominantly from two projects in the WHO Africa region where the bulk of the work was elective surgery for training purposes. Eight surgical projects were completely excluded (7 in health care gap settings, 1 that was in both natural disaster settings and health care gap settings, see appendix, missing data figure 1), all with a caseload of less than 100 operations and a short period of activity. The missing data were predominantly from projects with uncertified anaesthesia provider-led or physician anaesthesiologist expatriate led provision. This suggest the data were not missing completely at random and may risk introducing bias, although they comprised a small overall proportion of cases and available variables suggest the excluded cases were similar to the analysed dataset (see appendix, missing data table 2).

**DISCUSSION**

This is the largest observational study published from a humanitarian organisation describing the types of anaesthesia providers employed and the pattern of their work in a number of different settings. While not all humanitarian organisations (and MSF operational centres) operate in the same way as MSF-OCB, this study provides useful insights that may contribute towards their operational strategies.

Over 10 years of surgical activity by MSF-OCB, we found that anaesthesia provision was led by physician anaesthesiologists during 66% of all cases in HG and AC settings (bearing in mind physician anaesthesiologist-led does not mean physician anaesthesiologists administered the anaesthesia) with nurse anaesthetist-led provision accounting for 19% and uncertified anaesthesia provider-led provision accounting for 15% of cases. There was some variation in the surgical caseload between provider types: Physician anaesthesiologists were more commonly attached to projects with trauma-related surgery, while nurse anaesthetists were more commonly the most senior anaesthetic provider in projects with high numbers of obstetric surgery. All providers led during surgery on both very sick (ASA grade 5) and very young patients (aged only a few days), although majority of cases were minor surgery, which are less risky even in patients with a higher ASA class. In locations with uncertified anaesthesia provider-led anaesthesia, which was predominantly in the WHO Africa region, there was also a reduced presence of specialized surgical providers and expatriate involvement, despite the patient profile and surgical caseload being largely similar to that encountered in physician anaesthesiologist-led surgical projects in similar settings.

MSF tries to avoid employing uncertified anaesthesia providers, and they continue to evaluate means of mitigating this risk. However, a set of unique circumstances makes it unavoidable on occasion: 1) MSF, like many humanitarian organisations, operate predominantly in locations where there is a pre-existing anaesthesia workforce shortage,[15] and often in situations where this shortage may be exacerbated due to armed conflict or population displacement. 2) Expatriate staff are not always available, as MSF only deploy senior qualified anaesthesiologists as their expatriates, and it may not be possible for them to take time away from work at short notice. 3) Even if expatriate staff are available, in many



For peer review only - http://bmjopen.bmj.com/site/about/guidelines.xhtml

contexts they have become deliberate targets. This has led to more cautious deployment of expatriate personnel into volatile settings.[16]
In this study we report briefly on intra-operative mortality. Rates are comparable across the different lead providers and similar to other observational data from LMICs[17–21] and some humanitarian organisations (including other MSF operational centres),[4,22,23] while higher than other humanitarian organisations.[24,25] Such data must be interpreted cautiously as they should ideally be adjusted more fully for case-mix and severity. Further, most mortality related to surgery occurs in the days following surgery and not in theatre,[23,26,27] and these data are not available as part of the routine data we analysed. While a more appropriate and widely recognized measure of surgical outcomes is perioperative mortality, which is advocated by both the Lancet Commission on Global Surgery and the World Health Organisation.[28,29] we were unable to report this. Further research into surgical outcomes in the humanitarian setting, which includes perioperative mortality and the incidence of post-operative complications and how they might differ between different anaesthesia providers, would be useful to assist organisations in providing safe and efficient anaesthesia in resource limited situations.

**Limitations**

Data quality is a known issue when using surveillance data, and the occasionally unpredictable nature of working in humanitarian settings means there is a risk of further decline in quality. Due to the rigor in data monitoring centrally by MSF-OCB on a regular basis as described in the methods section, much has been done to minimize both missing data and improve the quality of the collected dataset. Our approach does have a particular risk of misclassification related to expatriate physician presence. Cases or projects could have been identified as ‘physician anaesthesiologist-led’, but the physician anaesthesiologist may not actually have been in the operating room for a variety of reasons including overseeing multiple theatres, or curfew and security concerns. Such misclassification could underrepresent the proportion of work where non-physicians were effectively sole providers. Our results therefore likely present a conservative estimate of the care provided by nurse anaesthetist and uncertified anaesthesia provider. Finally, it is important to note that some projects had started before the start of routine data collection in 2008. Projects with expatriate physician anaesthesiologists providing on-the-job training for uncertified anaesthesia providers in the period before 2008 will not be reflected in our dataset.

**CONCLUSION**

The majority of MSF anaesthesia care is done in teams where there are physician anaesthesiologists available. In conflict and healthcare gap settings, nurse anaesthetists and uncertified anaesthesia providers can be used as major providers. This study shows that the humanitarian sector has considerable experience with task sharing and shifting but further study of perioperative outcomes in these circumstances is needed to draw conclusions about how safe and practical it would be to apply to other settings. Despite their limitations routine data are key to monitoring the effectiveness of health systems, including humanitarian care, at scale and the MSF-OCB dataset is an important resource demonstrating that valuable data can be collected even in difficult circumstances. There is a need for wider engagement by the humanitarian community to continue to improve the collection and use of valid surgical

For peer review only

outcome data. This would promote learning on how to optimise the surgical and anaesthetic workforce and help to ensure safe surgical and anaesthetic care in the humanitarian sector.

## COMPETING INTERESTS

All authors except from SK, ME, and HE are employed by MSF-OCB.

## AUTHOR CONTRIBUTIONS

SK helped conceive the study design, analyse the data, interpret the data, write the initial draft of the manuscript, and edit the manuscript. MT helped conceive the study design, collect the data, interpret the data, and edit the manuscript. ENB, LH, AM, RH, CSV, KS, and AM helped collect the data and edit the manuscript. JK helped collect the data, interpret the data and edit the manuscript. HE and ME helped conceive the study design, interpret the data, and edit the manuscript.

## DATA SHARING STATEMENT

The data used in the study was provided by MT (SAGE Coordinator at MSF-Brussels at the time data was obtained), and contains de-identified case-level routine surgical surveillance data and programme-level data. All relevant data is available in the tables, figures and appendix.

## FUNDING

SK received funding from NIHR through their academic clinical fellowship scheme. ME received funding from a Wellcome Trust Senior Fellowship (#207522) as part of an unrelated research grant.

## LEGEND OF FIGURES

- Figure 1: Flow diagram showing inclusion/exclusion of data and points of data linkage

- Figure 2: World maps showing number of (A) Surgical projects and (B) Surgical cases in each WHO region in settings of (1) conflict, (2) health care gaps, and (3) natural disasters

- Figure 3: Timelines showing duration and point in time all included surgical projects were active

## REFERENCES

1 Meara JG, Leather AJM, Hagander L, *et al.* Global Surgery 2030: Evidence and solutions for achieving health, welfare, and economic development. *Lancet* 2015;**386**:569–624. doi:10.1016/S0140-6736(15)60160-X
2 Craven RM. Managing anaesthetic provision for global disasters. *Br J Anaesth* 2017;**119**:i126–34. doi:10.1093/bja/aex353
3 White MC, Randall K, Avara E, *et al.* Clinical Outcome, Social Impact and Patient


For peer review only - http://bmjopen.bmj.com/site/about/guidelines.xhtml

Expectation: a Purposive Sampling Pilot Evaluation of Patients in Benin Seven Years After Surgery. *World J Surg* 2018;**42**:1254–61. doi:10.1007/s00268-017-4296-9

4 Trudeau MON, Baron E, Hérard P, *et al.* Surgical care of pediatric patients in the humanitarian setting: The médecins sans frontières experience, 2012-2013. *JAMA Surg* 2015;**150**:1080–5. doi:10.1001/jamasurg.2015.1928

5 Ariyo P, Trelles M, Helmand R, *et al.* Providing Anesthesia Care in Resource-limited Settings: A 6-year Analysis of Anesthesia Services Provided at Médecins Sans Frontières Facilities. *Anesthesiology* 2016;**124**:561–9.

6 Chu KM, Ford N, Trelles M. Operative Mortality in Resource-Limited Settings: The Experience of Medecins Sans Frontières in 13 Countries. *Arch Surg* 2010;**145**:721–5.

7 Zitzman E, Berkley H, Jindal RM. Accountability in global surgery missions. *BMJ Glob Heal* 2018;**3**:e001025. doi:10.1136/bmjgh-2018-001025

8 Shrime MG, Sleemi A, Ravilla TD. Charitable platforms in global surgery:A systematic review of their effectiveness, cost-effectiveness, sustainability, and role training. *World J Surg* 2015;**39**:10–20. doi:10.1007/s00268-014-2516-0

9 Hendriks TCC, Botman M, Rahmee CNS, *et al.* Impact of short-term reconstructive surgical missions: A systematic review. *BMJ Glob Heal* 2019;**4**. doi:10.1136/bmjgh-2018-001176

10 Chu KM, Ford NP, Trelles M. Providing surgical care in Somalia: A model of task shifting. *Confl Health* 2011;**5**:12. doi:10.1186/1752-1505-5-12

11 Plecker E De, Zachariah R, Kumar AM V., *et al.* Emergency obstetric care in a rural district of Burundi: What are the surgical needs? *PLoS One* 2017;**12**:e0170882. doi:10.1371/journal.pone.0170882

12 World Health Organisation. Regional offices. https://www.who.int/about/who-we-are/regional-offices (accessed 4 Jul 2019).

13 Benchimol EI, Smeeth L, Guttmann A, *et al.* The REporting of studies Conducted using Observational Routinely-collected health Data (RECORD) statement. *PLoS Med* 2015;**12**:e1001885. doi:10.1371/journal.pmed.1001885

14 Foreign Medical Team Working Group. Classification and Minimum Standards for Foreign Medical Teams in Sudden Onset. Geneva: 2013.

15 Kempthorne P, Morriss WW, Mellin-Olsen J, *et al.* The WFSA Global Anesthesia Workforce Survey. *Anesth Analg* 2017;**125**:981–90. doi:10.1213/ANE.0000000000002258

16 ICRC. Health Care in Danger: A sixteen-country study. Geneva: 2011.

17 Kwari YD, Bello MR, Eni UE. Pattern of perioperative cardiac arrests at University of Maiduguri Teaching Hospital. *Niger J Med J Natl Assoc Resid Dr Niger* 2010;**19**:173–6.

18 Zoumenou E, Gbenou S, Assouto P, *et al.* Pediatric anesthesia in developing countries: experience in the two main university hospitals of Benin in West Africa. *Paediatr Anaesth* 2010;**20**:741–7.

19 Walker IA, Obua AD, Mouton F, *et al.* Paediatric surgery and anaesthesia in south-western Uganda: a cross-sectional survey. *Bull World Health Organ* 2010;**88**:897–906.

20 Rukewe A, Fatiregun A, Adebayo K. Anaesthesia for caesarean deliveries and maternal complications in a Nigerian teaching hospital. *African J Med Med Sci* 2014;**43**:5–10.

21 Rukewe A, Fatiregun A, Osunlaja TO. Cardiac arrest during anesthesia at a university Hospital in Nigeria. *Niger J Clin Pract* 2014;**17**:28–31.

22 Wichlas F, Tsitsilonis S, Guanti MD, *et al.* Orthopedic trauma surgeon in Sierra Leone: how to keep one's head over water. *Arch Orthop Trauma Surg* 2019;**0**:0. doi:10.1007/s00402-019-03171-1

23 Davies JF, Lenglet A, Van Wijhe M, *et al.* Perioperative mortality: Analysis of 3 years of operative data across 7 general surgical projects of Medecins Sans Frontieres in Democratic Republic of Congo, Central African Republic, and South Sudan. *Surgery* 2016;**159**:1269–78. doi:10.1016/j.surg.2015.12.022

24 Fisher QA, Nichols D, Stewart FC, *et al.* Assessing Pediatric Anesthesia Practices for Volunteer Medical Services Abroad. *Anesthesiol J Am Soc Anesthesiol* 2001;**95**:1315–22.

25 White MC, Barki BJ, Lerma SA, *et al.* A prospective observational study of



For peer review only - http://bmjopen.bmj.com/site/about/guidelines.xhtml

anesthesia- related adverse events and postoperative complications occurring during a surgical mission in Madagascar. *Anesth Analg* 2018;**127**:506–12.

26 Biccard BM, Madiba TE, Kluyts H-L, *et al.* Perioperative patient outcomes in the African Surgical Outcomes Study: a 7-day prospective observational cohort study. *Lancet* 2018;**391**:1589–98. doi:10.1016/S0140-6736(18)30001-1

27 Ng-Kamstra JS, Arya S, Greenberg SLM, *et al.* Perioperative mortality rates in low-income and middle-income countries: a systematic review and meta-analysis. *BMJ Glob Heal* 2018;**3**:e000810. doi:10.1136/bmjgh-2018-000810

28 Holmer H, Bekele A, Hagander L, *et al.* Evaluating the collection, comparability and findings of six global surgery indicators. *Br J Surg* 2019;:e138–50. doi:10.1002/bjs.11061

29 World Health Organisation. 2018 Global reference list of 100 core health indicators (plus health-related SDGs). Geneva: 2018.

For peer review only



For peer review only - http://bmjopen.bmj.com/site/about/guidelines.xhtml

**Figure 1: Flow diagram showing inclusion/exclusion of data and points of data linkage**

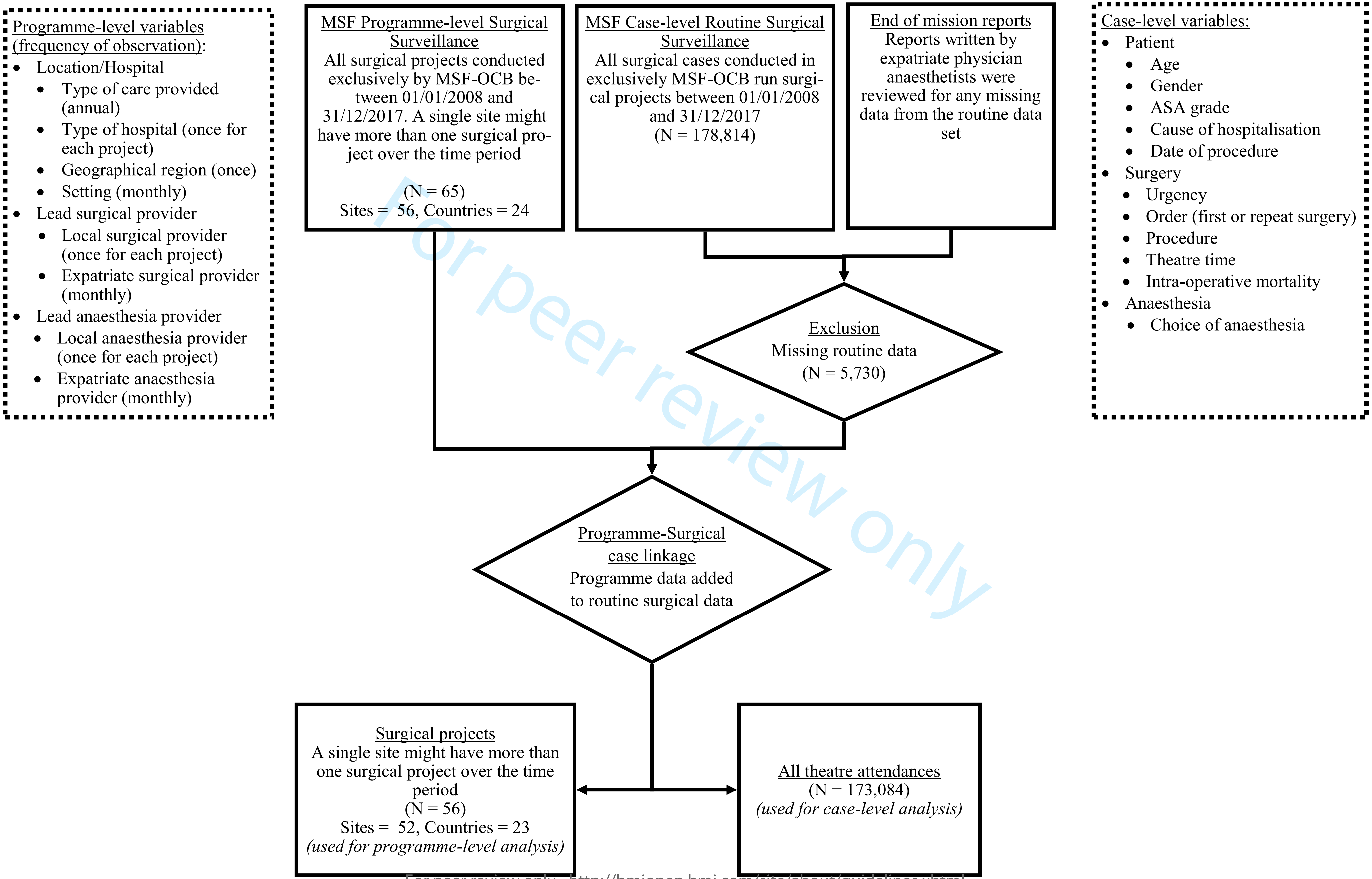


For peer review only

For peer review only - http://bmjopen.bmj.com/site/about/guidelines.xhtml

Figure 2: World maps showing number of (A) Surgical projects and (B) Surgical cases in each WHO region in settings of (1) conflict, (2) health care gaps, and (3) natural disasters

**A**

| WHO regions | Conflict setting | Health care gap setting | Natural disaster setting |
|---|---|---|---|
| Africa region | 21 | 21 | 0 |
| Americas region | 0 | 3 | 3 |
| Eastern Mediterranean region | 7 | 8 | 0 |
| South−East Asia region | 0 | 2 | 1 |
| Western Pacific region | 0 | 0 | 1 |

1 2 3

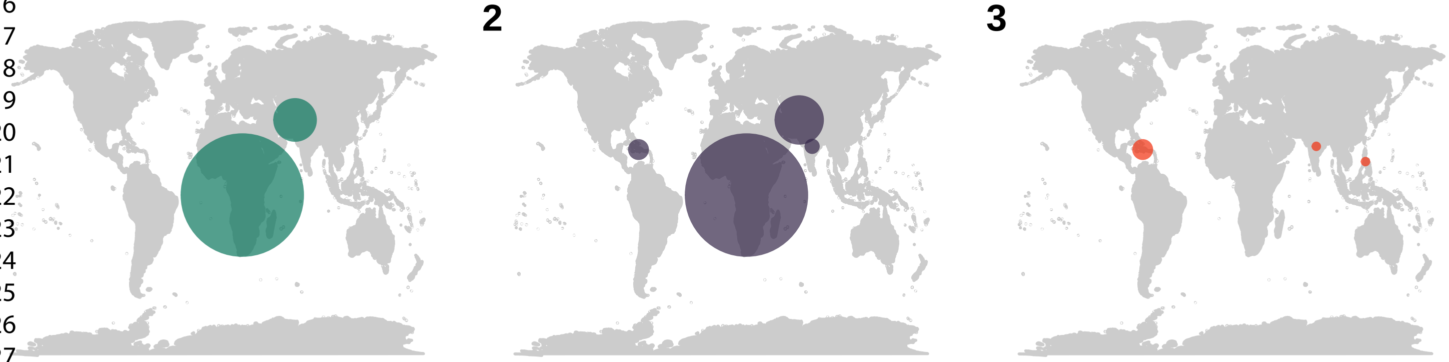

**B**

| WHO regions | Conflict setting | Health care gap setting | Natural disaster setting |
|---|---|---|---|
| Africa region | 16087 | 65551 | 0 |
| Americas region | 0 | 33319 | 2730 |
| Eastern Mediterranean region | 26138 | 28066 | 0 |
| South−East Asia region | 0 | 815 | 89 |
| Western Pacific region | 0 | 0 | 289 |

1 2 3

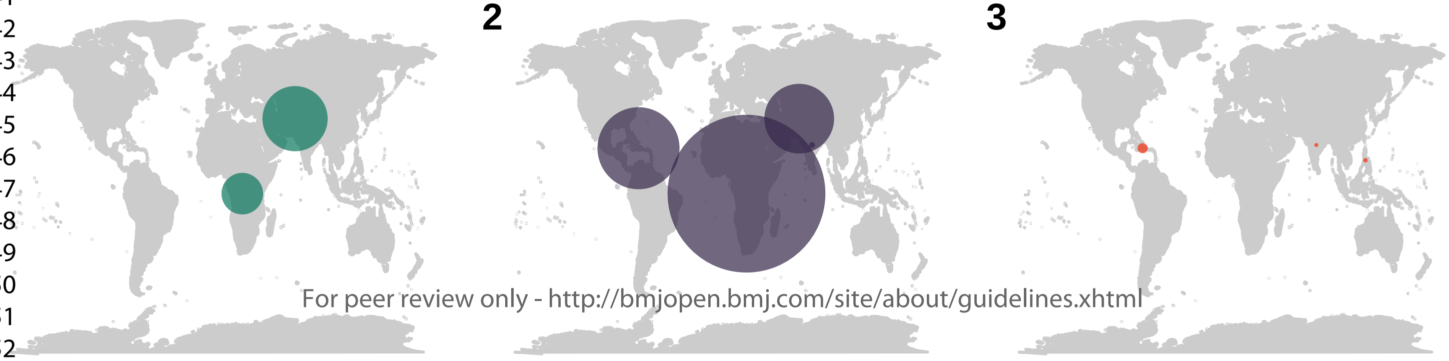

For peer review only - http://bmjopen.bmj.com/site/about/guidelines.xhtml

Figure 3: Timelines showing duration and point in time all included surgical projects were active

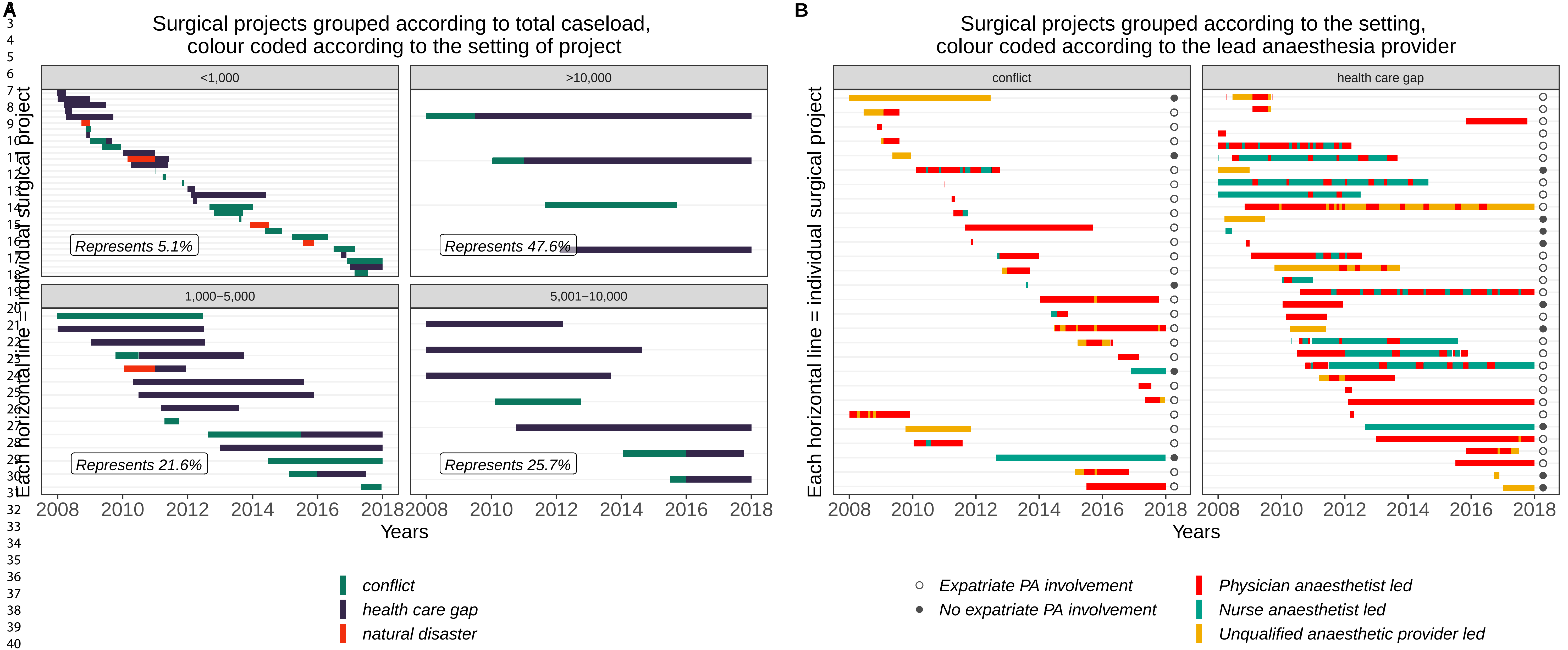




*Excludes periods where projects are run in collaboration with other organisations or local government. Additionally, only data from 2008 till 2017 are included. Therefore, periods with expatriate physician anaesthetist (PA) involvement before then are not reflected here.

# Anaesthesia care providers employed in humanitarian settings by Médecins Sans Frontières: A retrospective observational study of 173,084 surgical cases over 10 years

## APPENDIX

For peer review only

Table of Contents

Appendix Table 1: Variables used in the study

Appendix Table 2: surgical projects in health care gap settings in the WHO SEA region (2 in total)

Appendix Table 3: Surgical projects in natural disaster settings (5 in total)

Appendix Table 4: Surgical groupings as used in main table 2 (case-level data)

Missing data Table 1: Number of missing values within each variable

Missing data Figure 2: Proportion of missing data in each surgical project

Missing data Table 2: overview of available data

## Appendix Table 1: Variables used in the study

<table>
<tr><th>Key characteristic group</th><th>Name of variable</th><th>Type of data</th><th>Description of data</th><th>Source</th></tr>
<tr><td rowspan="5">Patient</td><td>Age</td><td>Continuous</td><td>For patients below the age of 2 (typed in original data collection sheet as days and months), the age has been converted for analysis to a fraction of a year.</td><td>Case-level routine data</td></tr>
<tr><td>Gender</td><td>Categorical (binary)</td><td>Either male or female</td><td>Case-level routine data</td></tr>
<tr><td>Date of procedure</td><td>Continuous</td><td>Date operation took place</td><td>Case-level routine data</td></tr>
<tr><td>ASA grade</td><td>Ordinal</td><td>American Society of Anaesthesiologists physical status classification system (ASA). Discrete numeric scale between 1 and 5 (1 = normal healthy patient, 5 = moribund patient expected not to survive without surgery) of the patient’s physical health prior to surgery.</td><td>Case-level routine data</td></tr>
<tr><td>Cause of hospitalisation</td><td>Categorical (nominal)</td><td>3 letter code used as defined by MSF-OCB operational departmental guideline. A total of 24 codes available, and grouped into 4 distinct categories:<br>- Accidental trauma<br>- Violent trauma<br>- Obstetric<br>- Other (including but not limited to tropical disease, tumours, obstruction)</td><td>Case-level routine data</td></tr>
<tr><td rowspan="4">Surgery</td><td>Urgency</td><td>Categorical (ordinal)</td><td>3 values available, relating to how soon the surgical procedure has to occur:<br>- “Urgent” (labelled “emergent” in manuscript) = requiring immediate surgery<br>- “Delayed” (labelled “urgent” in manuscript) = requiring surgery during current hospital admission<br>- “Planned” (labelled “elective” in manuscript) = elective surgery</td><td>Case-level routine data</td></tr>
<tr><td>Order</td><td>Categorical (ordinal)</td><td>3 codes available, relating to whether patient has had surgery before during admission:<br>- “First” = first time entering theatre<br>- “Unplanned” = unplanned return to theatre<br>- “Re-intervention” = planned return to theatre</td><td>Case-level routine data</td></tr>
<tr><td>Procedure – Main group</td><td>Categorical (nominal)</td><td>2 letter code used as defined by MSF-OCB operational department guidelines. A total of 36 procedures codes available, and grouped into the main categories of surgery. See appendix table 4 for full breakdown.</td><td>Case-level routine data</td></tr>
<tr><td>Lead surgical provider</td><td>Categorical (nominal)</td><td>A merged variable, based on the presence of local and expatriate providers as outlined below. MD signifies a physician without surgical qualifications but with surgical skills.
<table>
<tr><th></th><th>Local provider</th><th>Expatriate provider</th></tr>
<tr><td rowspan="4">General & specialist surgeon</td><td>None</td><td>General & Specialist</td></tr>
<tr><td>MD</td><td>General & Specialist</td></tr>
<tr><td>General</td><td>Specialist</td></tr>
<tr><td>General & Specialist</td><td>Any</td></tr>
<tr><td rowspan="2">Specialist only</td><td>None</td><td>Specialist</td></tr>
<tr><td>MD</td><td>Specialist</td></tr>
</table></td><td>Programme-level routine data (MT)</td></tr>
</table>

<table>
<tr><td></td><td></td><td></td><td>
<table>
<tr><td></td><td>Specialist</td><td>Specialist or none</td></tr>
<tr><td rowspan="3">General only</td><td>None</td><td>General</td></tr>
<tr><td>MD</td><td>General</td></tr>
<tr><td>General</td><td>General or none</td></tr>
<tr><td>MD</td><td>MD</td><td>None</td></tr>
</table>
</td><td></td></tr>
<tr><td></td><td>Theatre time (min)</td><td>Continuous</td><td>Time in minutes the patient was occupying theatre. This included anaesthetic and surgical time, as well as any recovery of patient, which occurred in theatre.</td><td>Case-level routine data</td></tr>
<tr><td></td><td>Intra-operative mortality</td><td>Categorical (binary)</td><td>Whether the patient was dead or alive by the time they left recovery. For the purpose of the study, this is considered as intraoperative mortality.</td><td>Case-level routine data</td></tr>
<tr><td rowspan="2">Anaesthesia</td><td>Choice of anaesthesia</td><td>Categorical (nominal)</td><td>List of 7 codes of anaesthesia that can be provided. Only a single code can be used for a surgical procedure:<br>- Local anaesthesia<br>- Regional anaesthesia<br>- Spinal anaesthesia<br>- General anaesthesia without intubation or muscle relaxant<br>- General anaesthesia with intubation and/or muscle relaxant<br>- Combined anaesthesia (if more than one code need to be used, e.g. spinal anaesthesia + general anaesthesia)<br>- Other anaesthesia, e.g. sedation</td><td>Case-level routine data</td></tr>
<tr><td>Lead anaesthesia provider</td><td>Categorical (nominal)</td><td>A merged variable, based on the presence of local and expatriate providers as outlined below.
<table>
<tr><td></td><td>Local provider</td><td>Expatriate provider</td></tr>
<tr><td rowspan="4">Physician anaesthesiologist (PA) led</td><td>PA</td><td>none</td></tr>
<tr><td>NA</td><td>PA</td></tr>
<tr><td>UA</td><td>PA</td></tr>
<tr><td>None</td><td>PA</td></tr>
<tr><td rowspan="3">Nurse anaesthetist (NA) led</td><td>NA</td><td>none</td></tr>
<tr><td>UA</td><td>NA</td></tr>
<tr><td>None</td><td>NA</td></tr>
<tr><td>Uncertified anaesthetic provider (UA) led</td><td>UA</td><td>None</td></tr>
</table>
</td><td>Programme-level routine data (MT)</td></tr>
<tr><td>Setting</td><td>Type of care provided by hospital</td><td>Categorical (nominal)</td><td>Overall type of provision provided during surgical project, based on MSF-OCB brief. 9 separate categories grouped into 5:<br>- Emergency only<br>- Capacity to perform both emergency and elective surgery<br>- Maternity care only<br>- Trauma care only<br>- Other specific care provision (wound care, trauma and surgical care, obstetric fistula care, and surgical care of typhoid related complications)</td><td>Programme-level routine data (MT)</td></tr>
</table>

| | | | | |
|---|---|---|---|---|
| | WHO region | Categorical (nominal) | The location of each mission was labelled according to the region codes used by the World Health Organisation:<br>- AFR<br>- EMR<br>- SEAR<br>- AMR<br>- WPR | WHO |
| | Setting | Categorical (binary) | 3 variables:<br>- Conflict<br>- Natural disaster<br>- Health care gaps | Programme-level routine data (MT) |
| | Hospital level | Ordinal (Categorical) | 4 distinct categories as per MSF-OCB surgical policy guidelines:<br>- Sole remit hospital = hospital that provides care for a specific purpose (i.e. not necessarily a quaternary referral hospital, but a surgical setup for a specific indication). Examples include: Trauma centre, Maternity centre, Fistula repair camp.<br>- Referral hospital = provincial hospital, considered tertiary referral hospital.<br>- District hospital = can manage most, but will refer complex cases on to referral hospitals.<br>- Health centres = small rural health centres with capacity to perform basic surgical operations | Programme-level routine data (MT) |
| | Site ID | Character | Anonymous unique code for each site | produced in R 3.6 |

For peer review only

**Appendix Table 2: surgical projects in health care gap settings in the WHO SEA region (2 in total)**

| | |
|---|---|
| **Type of anaesthesia provider** | Physician anaesthetist only |
| **Type of hospital** | - District hospital<br>- Health centre |
| **Type of care provided by surgical project (No. of projects)** | - Capacity to perform both emergency and elective surgery<br>- Filiariasis-related care |
| **Total duration, months** | 19 |
| **Type of surgical provider for entire duration** | - General & specialty surgeons<br>- General surgeon only |
| **Total number of all cases, No.** | 815 |
| **Main cause for hospitalisation, No. (%)** | - Other, 482 (59) |
| **Main category of surgery, No. (%)** | - Minor Surgery, 507 (62) |
| **Intra-operative mortality, No. (%)** | 2 (0.2) |

For peer review only

**Appendix Table 3: Surgical projects in natural disaster settings (5 in total)**

| | |
|---|---|
| **Type of anaesthesia provider** | Physician anaesthetist only |
| **Type of hospital** | - District<br>- Sole remit hospital |
| **Type of care provided by surgical project (No. of projects)** | - Capacity to perform both emergency and elective surgery (2)<br>- Trauma care only (1)<br>- Emergency only (2) |
| **Total duration, months** | 40 |
| **Presence of surgical provider, months** | |
| - **General and specialty surgeons** | 23 |
| - **General surgeon only** | 16 |
| - **Specialty surgeon only** | 1 |
| **Total number of all cases, No. (%)** | 3108 |
| **Main cause for hospitalisation, No. (%)** | - Other, 1144 (37) |
| **Main category of surgery, No. (%)** | - Minor surgery, 1608 (52) |
| **Intra-operative mortality, No. (%)** | 9 (0.3) |

For peer review only

**Appendix Table 4: Surgical groupings as used in main table 2 (case-level data)**

| Surgical grouping | Examples of types of surgery included |
|---|---|
| **Minor surgery** | • Simple wound treatment<br>• Insertion/removal of drain<br>• Burns dressing change<br>• Wound debridement<br>• Removal of foreign body<br>• Amputation of digits or toes<br>Incl. procedure codes with median operative time < 45min within the dataset:<br>• Curettage post delivery (GP)<br>• Reduction of fractures (OR)<br>• Removal of osteosynthesis (OX)<br>• Ophthalmic surgery (SO) |
| **Caesarean section** | • Caesarean section only |
| **Visceral surgery** | • Exploratory laparotomy<br>• Hernia repair<br>• Resection/repair solid organs (e.g. spleen/liver) or gut |
| **Orthopaedic surgery** | • External or internal fixation of fracture<br>• Surgery to any joint<br>• Limb amputation (excluding digits or toes)<br>• Curettage for osteomyelitis |
| **Obstetric & gynaecological surgery (excl. Caesarean section)** | • Management of ectopic pregnancy<br>• Obstetric fistula repair<br>• Hysterectomy |
| **Specialties** | • Urology<br>• Vascular surgery<br>• Plastic and reconstructive surgery<br>• Ear, nose and throat surgery<br>• Neurosurgery<br>• Thoracic surgery<br>• Maxillofacial surgery<br>• Other specialized surgery |

Modified from the original surgical groupings outlined in the "MSF-OCB Operating Department Data Collection Guidelines (2015)".

For peer review only - http://bmjopen.bmj.com/site/about/guidelines.xhtml

**Missing data Table 1: Number of missing values within each variable**

| Variable | Number missing |
|---|---|
| **ASA** | 3232 |
| **Intra-operative mortality** | 2154 |
| **Time in theatre** | 1922 |
| **Age** | 47 |
| **Main procedure** | 5 |
| **Choice of anaesthesia** | 2 |
| **Gender** | 0 |
| **Date of procedure** | 0 |
| **Cause of hospitalisation** | 0 |
| **Urgency** | 0 |
| **Order** | 0 |
| **Surgical provider** | 0 |
| **Anaesthesia provider** | 0 |
| **Who region** | 0 |
| **Setting** | 0 |
| **Type of hospital** | 0 |
| **Type of care provided** | 0 |
| **Site ID** | 0 |

For peer review only

Missing Data Figure 1: Proportion of missing data in each surgical project

histogram showing all surgical projects (along x−axis), sorted according to setting,
showing proportion of missing cases with any missing data (red indicates data with at least one missing value)

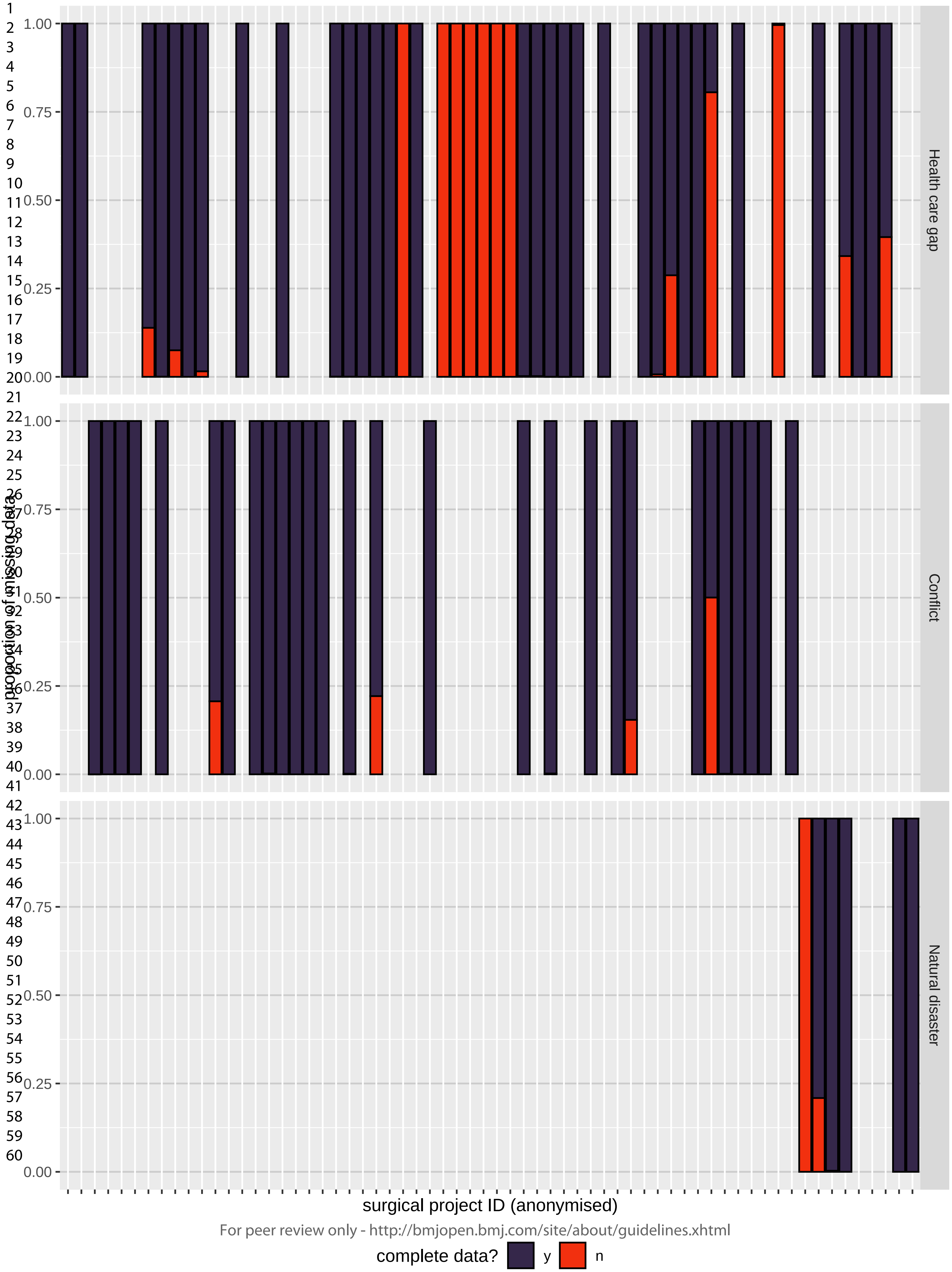

**Missing data Table 2: overview of available data**

| Variable | Value |
|---|---|
| **Median age, years (IQR)** | 26 (18-36) |
| **Female, no. (%)** | 2963 (52) |
| **Median ASA (IQR)** | 1 (1-2) |
| **Emergent surgery, no. (%)** | 2277 (40) |
| **Cause for hospitalisation, no. (%)** | |
| - **Obstetric** | 1188 |
| - **Any trauma** | 2074 |
| - **Other** | 2468 |
| **Main surgical procedure** | |
| - **Minor surgery** | 2854 |
| - **Caesarean section** | 746 |
| - **Visceral surgery** | 1408 |
| - **Obstetric and gynaecology** | 503 |
| - **Orthopaedics** | 177 |
| - **Other specialty surgery** | 37 |
| **Intraoperative mortality** | |
| - **Alive** | 3563 |
| - **Died** | 13 |
| **Median theatre time, minutes (IQR)** | 45 (35-60) |
| **Setting** | |
| - **Health care gap** | 3359 |
| - **Conflict** | 1443 |
| - **Natural disaster** | 928 |

For peer review only

**The use of different anaesthesia providers in humanitarian settings: Descriptive study of 173,084 episodes of surgical care provided by Médecins Sans Frontières over 10 years**

The RECORD statement – checklist of items, extended from the STROBE statement, that should be reported in observational studies using routinely collected health data.

| | Item No. | STROBE items | Location in manuscript where items are reported | RECORD items | Location in manuscript where items are reported |
|---|---|---|---|---|---|
| **Title and abstract** | | | | | |
| | 1 | (a) Indicate the study's design with a commonly used term in the title or the abstract<br><br>(b) Provide in the abstract an informative and balanced summary of what was done and what was found | Title<br>Page 1<br><br>Abstract<br>Page 4 | RECORD 1.1: The type of data used should be specified in the title or abstract. When possible, the name of the databases used should be included.<br><br>RECORD 1.2: If applicable, the geographic region and timeframe within which the study took place should be reported in the title or abstract.<br><br>RECORD 1.3: If linkage between databases was conducted for the study, this should be clearly stated in the title or abstract. | Abstract<br>Page 4<br><br>Abstract<br>Page 4<br><br>Abstract<br>Page 4 |
| **Introduction** | | | | | |
| Background rationale | 2 | Explain the scientific background and rationale for the investigation being reported | Introduction<br>Page 6 | | |
| Objectives | 3 | State specific objectives, including any prespecified hypotheses | Introduction<br>Page 6 | | |
| **Methods** | | | | | |
| Study Design | 4 | Present key elements of study design early in the paper | Methods<br>Page 6 | | |
| Setting | 5 | Describe the setting, locations, and relevant dates, including | Methods<br>Page 6-8 | | |


For peer review only

| | | | | | |
|---|---|---|---|---|---|
| | | periods of recruitment, exposure, follow-up, and data collection | | | |
| Participants | 6 | *(a) Cohort study* - Give the eligibility criteria, and the sources and methods of selection of participants. Describe methods of follow-up<br>*Case-control study* - Give the eligibility criteria, and the sources and methods of case ascertainment and control selection. Give the rationale for the choice of cases and controls<br>*Cross-sectional study* - Give the eligibility criteria, and the sources and methods of selection of participants<br><br>*(b) Cohort study* - For matched studies, give matching criteria and number of exposed and unexposed<br>*Case-control study* - For matched studies, give matching criteria and the number of controls per case | N/A<br>N/A<br>N/A<br>N/A<br>N/A | RECORD 6.1: The methods of study population selection (such as codes or algorithms used to identify subjects) should be listed in detail. If this is not possible, an explanation should be provided.<br><br>RECORD 6.2: Any validation studies of the codes or algorithms used to select the population should be referenced. If validation was conducted for this study and not published elsewhere, detailed methods and results should be provided.<br><br>RECORD 6.3: If the study involved linkage of databases, consider use of a flow diagram or other graphical display to demonstrate the data linkage process, including the number of individuals with linked data at each stage. | Methods Page 7<br><br>N/A<br><br>Inclusion flow diagram |
| Variables | 7 | Clearly define all outcomes, exposures, predictors, potential confounders, and effect modifiers. Give diagnostic criteria, if applicable. | Appendix Table 1 | RECORD 7.1: A complete list of codes and algorithms used to classify exposures, outcomes, confounders, and effect modifiers should be provided. If these cannot be reported, an explanation should be provided. | Appendix Table 1 |
| Data sources/ measurement | 8 | For each variable of interest, give sources of data and details of methods of assessment (measurement). | Appendix Table 1 | | |

For peer review only

| | | | | | |
|---|---|---|---|---|---|
| | | Describe comparability of assessment methods if there is more than one group | N/A | | |
| Bias | 9 | Describe any efforts to address potential sources of bias | Methods – bias<br>Page 7 | | |
| Study size | 10 | Explain how the study size was arrived at | Descriptive study – not performed | | |
| Quantitative variables | 11 | Explain how quantitative variables were handled in the analyses. If applicable, describe which groupings were chosen, and why | Appendix<br>Table 1 | | |
| Statistical methods | 12 | (a) Describe all statistical methods, including those used to control for confounding<br>(b) Describe any methods used to examine subgroups and interactions<br>(c) Explain how missing data were addressed<br>(d) *Cohort study* - If applicable, explain how loss to follow-up was addressed<br>*Case-control study* - If applicable, explain how matching of cases and controls was addressed<br>*Cross-sectional study* - If applicable, describe analytical methods taking account of sampling strategy<br>(e) Describe any sensitivity analyses | Descriptive study – not performed<br>Methods – bias<br>Page 7<br>Results (page 15) appendix (5. Missing data, table 1 & 2, Figure 1)<br>n/a<br>n/a<br>n/a<br>n/a | | |
| Data access and cleaning methods | | .. | | RECORD 12.1: Authors should describe the extent to which the investigators had access to the database | Methods<br>Page 7 |

For peer review only

| | | | | | |
|---|---|---|---|---|---|
| | | | | population used to create the study population.<br><br>RECORD 12.2: Authors should provide information on the data cleaning methods used in the study. | Methods Page 6 |
| Linkage | | .. | | RECORD 12.3: State whether the study included person-level, institutional-level, or other data linkage across two or more databases. The methods of linkage and methods of linkage quality evaluation should be provided. | Methods (page 6) & inclusion flow diagram |
| **Results** | | | | | |
| Participants | 13 | (a) Report the numbers of individuals at each stage of the study (*e.g.*, numbers potentially eligible, examined for eligibility, confirmed eligible, included in the study, completing follow-up, and analysed)<br>(b) Give reasons for non-participation at each stage.<br>(c) Consider use of a flow diagram | inclusion/exclusion diagram<br><br>N/A<br><br>inclusion/exclusion diagram | RECORD 13.1: Describe in detail the selection of the persons included in the study (*i.e.,* study population selection) including filtering based on data quality, data availability and linkage. The selection of included persons can be described in the text and/or by means of the study flow diagram. | Methods (page 7) & inclusion/exclusio n diagram |
| Descriptive data | 14 | (a) Give characteristics of study participants (*e.g.*, demographic, clinical, social) and information on exposures and potential confounders<br>(b) Indicate the number of participants with missing data for each variable of interest<br>(c) *Cohort study* - summarise follow-up time (*e.g.*, average and total amount) | Results – table 1<br><br>Appendix (5. Missing data, table 1)<br><br>N/A | | |

For peer review only

| | | | | | |
|---|---|---|---|---|---|
| Outcome data | 15 | *Cohort study* - Report numbers of outcome events or summary measures over time<br>*Case-control study* - Report numbers in each exposure category, or summary measures of exposure<br>*Cross-sectional study* - Report numbers of outcome events or summary measures | N/A<br>N/A<br>Results Page 8 | | |
| Main results | 16 | (a) Give unadjusted estimates and, if applicable, confounder-adjusted estimates and their precision (e.g., 95% confidence interval). Make clear which confounders were adjusted for and why they were included<br>(b) Report category boundaries when continuous variables were categorized<br>(c) If relevant, consider translating estimates of relative risk into absolute risk for a meaningful time period | Results Page 8 onwards + table 1 (page 10) and 2 (page 13)<br>Results table 1 (page 10) and 2 (page 13)<br>N/A | | |
| Other analyses | 17 | Report other analyses done—e.g., analyses of subgroups and interactions, and sensitivity analyses | N/A | | |
| **Discussion** | | | | | |
| Key results | 18 | Summarise key results with reference to study objectives | Discussion Page 16 | | |
| Limitations | 19 | Discuss limitations of the study, taking into account sources of potential bias or imprecision. Discuss both direction and magnitude of any potential bias | Discussion Page 16 | RECORD 19.1: Discuss the implications of using data that were not created or collected to answer the specific research question(s). Include discussion of misclassification bias, unmeasured confounding, missing | Discussion Page 16 |

For peer review only

| | | | | | |
|---|---|---|---|---|---|
| | | | | data, and changing eligibility over time, as they pertain to the study being reported. | |
| Interpretation | 20 | Give a cautious overall interpretation of results considering objectives, limitations, multiplicity of analyses, results from similar studies, and other relevant evidence | Discussion Page 16 | | |
| Generalisability | 21 | Discuss the generalisability (external validity) of the study results | Discussion Page 16 | | |
| **Other Information** | | | | | |
| Funding | 22 | Give the source of funding and the role of the funders for the present study and, if applicable, for the original study on which the present article is based | Page 17 | | |
| Accessibility of protocol, raw data, and programming code | | .. | | RECORD 22.1: Authors should provide information on how to access any supplemental information such as the study protocol, raw data, or programming code. | Page 17 |

*Reference: Benchimol EI, Smeeth L, Guttmann A, Harron K, Moher D, Petersen I, Sørensen HT, von Elm E, Langan SM, the RECORD Working Committee. The REporting of studies Conducted using Observational Routinely-collected health Data (RECORD) Statement. *PLoS Medicine* 2015; in press.

*Checklist is protected under Creative Commons Attribution (CC BY) license.

For peer review only
